# Supplementary material for: Inference of the HIV-1 VRC01 Antibody Lineage Unmutated Common Ancestor Reveals Alternative Pathways to Overcome a Key Glycan Barrier
Source: Immunity. 2018 Dec 18;49(6):1162–1174.e8. doi: 10.1016/j.immuni.2018.10.015 (PMC6303191; doi:10.1016/j.immuni.2018.10.015)
Supplement: Document S1. Figures S1–S6 and Tables S1–S3 [file mmc1.pdf]

Immunity, Volume *49*

Supplemental Information

Inference of the HIV-1 VRC01 Antibody Lineage Unmutated Common Ancestor Reveals Alternative Pathways to Overcome a Key Glycan Barrier

Mattia Bonsignori, Eric Scott, Kevin Wiehe, David Easterhoff, S. Munir Alam, Kwan-Ki Hwang, Melissa Cooper, Shi-Mao Xia, Ruijun Zhang, David C. Montefiori, Rory Henderson, Xiaoyan Nie, Garnett Kelsoe, M. Anthony Moody, Xuejun Chen, M. Gordon Joyce, Peter D. Kwong, Mark Connors, John R. Mascola, Andrew T. McGuire, Leonidas Stamatatos, Max Medina-Ramírez, Rogier W. Sanders, Kevin O. Saunders, Thomas B. Kepler, and Barton F. Haynes

Table S1. IgH and IgL chain sequences of GL VRC01-class mAbs produced in this study. Related to Figure 2.

VRC01 VJ.Rev	VH/JH reverted	QVQLVQSGAEVKKPGASVKVSCKASGYTFTGYYMHWVRQAPGQGLEWMGWINPNSGGTNYAQKFQGRVTMTRDTSISTAYMELSRLRSDDTAVYYCARGKNSDYNWDFQHWGQGTLVTVSS
	VL/JL reverted	EIVLTQSPATLSLSPGERATLSCRASQSVSSYLAWYQQKPGQAPRLLIYDASNRATGIPARFSGSGSGTDFTLTISSLEPEDFAVYYCQQYEFFGQGTKLEIK
VRC01 V.Rev	VH reverted	QVQLVQSGAEVKKPGASVKVSCKASGYTFTGYYMHWVRQAPGQGLEWMGWINPNSGGTNYAQKFQGRVTMTRDTSISTAYMELSRLRSDDTAVYYCARGKNCDYNWDFEHWGRGTPVIVSS
	VL reverted	EIVLTQSPGTLSLSPGERATLSCRASQSVSSSYLAWYQQKPGQAPRLLIYGASSRATGIPDRFSGSGSGTDFTLTISRLEPEDFAVYYCQQYEFFGQGTKVQVD
VRC03 V.Rev	VH reverted	QVQLVQSGAEVKKPGASVKVSCKASGYTFTGYYMHWVRQAPGQGLEWMGWINPNSGGTNYAQKFQGRVTMTRDTSISTAYMELSRLRSDDTAVYYCARRGSCDYCGDFPWQYWGQGTVVVVSS
	VL reverted	EIVLTQSPGTLSLSPGERATLSCRASQSVSSSYLAWYQQKPGQAPRLLIYGASSRATGIPDRFSGSGSGTDFTLTISRLEPEDFAVYYCQQFEFFGLGSELEVH
3BNC60 V.Rev	VH reverted	QVQLVQSGAEVKKPGASVKVSCKASGYTFTGYYMHWVRQAPGQGLEWMGWINPNSGGTNYAQKFQGRVTMTRDTSISTAYMELSRLRSDDTAVYYCARQRSDFWDFDVWGSGTQVTVSS
	VL reverted	DIQMTQSPSSLSASVGDRVTITCQASQDISNYLNWYQQKPGKAPKLLIYDASNLETGVPSRFSGSGSGTDFTFTISSLQPEDIATYYCQVYEFIVPGTRLDLK
3BNC117 V.Rev	VH reverted	QVQLVQSGAEVKKPGASVKVSCKASGYTFTGYYMHWVRQAPGQGLEWMGWINPNSGGTNYAQKFQGRVTMTRDTSISTAYMELSRLRSDDTAVYYCARQRSDYWDFDVWGSGTQVTVSS
	VL reverted	DIQMTQSPSSLSASVGDRVTITCQASQDISNYLNWYQQKPGKAPKLLIYDASNLETGVPSRFSGSGSGTDFTFTISSLQPEDIATYYCQVYEFVVPGTRLDLK
12A12 V.Rev	VH reverted	QVQLVQSGAEVKKPGASVKVSCKASGYTFTGYYMHWVRQAPGQGLEWMGWINPNSGGTNYAQKFQGRVTMTRDTSISTAYMELSRLRSDDTAVYYCARDGSGDDTSWHLDPWGQGTLVIVSS
	VL reverted	DIQMTQSPSSLSASVGDRVTITCQASQDISNYLNWYQQKPGKAPKLLIYDASNLETGVPSRFSGSGSGTDFTFTISSLQPEDIATYYCAVLEFFGPGTKVEIK
VRC20 V.Rev	VH reverted	QVQLVQSGAEVKKPGASVKVSCKASGYTFTGYYMHWVRQAPGQGLEWMGWINPNSGGTNYAQKFQGRVTMTRDTSISTAYMELSRLRSDDTAVYYCARRMRSQDREWDFQHWGQGTRIIVSS
	VL reverted	QSALTQPASVSGSPGQSITISCTGTSSDVGGYNYVSWYQQHPGKAPKLMIYEVSNRPSGVSNRFSGSKSGNTASLTISGLQAEDEADYYCNAFEFFGGGTKLTVL
VRC23 V.Rev	VH reverted	QVQLVQSGAEVKKPGASVKVSCKASGYTFTGYYMHWVRQAPGQGLEWMGWINPNSGGTNYAQKFQGRVTMTRDTSISTAYMELSRLRSDDTAVYYCARGVRRDASWWLQFWGQGTLVTVSS
	VL reverted	EIVMTQSPATLSVSPGERATLSCRASQSVSSNLAWYQQKPGQAPRLLIYGASTRATGIPARFSGSGSGTEFTLTISSLQSEDFAVYYCQQYETFGQGTKVEIK
VRC18b V.Rev	VH reverted	QVQLVQSGAEVKKPGASVKVSCKASGYTFTGYYMHWVRQAPGQGLEWMGWINPNSGGTNYAQKFQGRVTMTRDTSISTAYMELSRLRSDDTAVYYCARFAGYEWSFIWGQGTLVIVSS
	VL reverted	EIVLTQSPGTLSLSPGERATLSCRASQSVSSSYLAWYQQKPGQAPRLLIYGASSRATGIPDRFSGSGSGTDFTLTISRLEPEDFAVYYCQILEFFGRGTRVEMN

Table S2. Neutralization breadth and potency (IC_{50}, μg/ml) of VRC01 lineage antibodies. Related to Figure 5.

		IC50 ug/ml															
Clade	Ab ID	BJOX2000	CE1176	X1632	X2278	398F1	25710	CNE8	TRO11	246F3	CE0217	CH119	CNE55	MLV-SVA	Positives	% neut	GeoMean
3+6	VRC03g	>50	>50	0.023	0.023	0.113	0.234	7.333	0.064	34.983	10.777	1.695	0.311	>50	10	83.3	0.51
3+6	VRC03	>50	>50	0.036	0.023	0.163	0.146	47.416	0.162	>50	>50	5.942	0.847	>50	8	66.7	0.41
3+6	VRC03b	>50	>50	0.168	0.023	0.783	1.071	3.167	2.699	28.487	>50	>50	3.316	>50	8	66.7	1.13
3+6	DH651.9	>50	>50	0.411	0.023	0.674	0.787	2.6	2.175	>50	>50	>50	10.797	>50	7	58.3	0.84
3+6	VRC03f	>50	>50	0.023	0.023	0.067	0.099	4.685	0.036	41.524	31.667	1.48	0.092	>50	10	83.3	0.40
3+6	VRC03e	n/a	n/a	n/a	n/a	n/a	n/a	n/a	n/a	n/a	n/a	n/a	n/a	n/a	n/a	n/a	n/a
3+6	VRC03i	>50	3.606	5.292	0.023	1.231	0.092	42.641	2.605	>50	>50	19.746	6.551	>50	9	75.0	2.08
3+6	VRC03h	>50	6.837	0.023	0.023	0.028	0.027	12.122	0.023	>50	7.799	1.22	0.105	>50	10	83.3	0.24
3+6	VRC03d	>50	>50	0.028	0.023	0.07	0.085	18.503	0.044	>50	25.903	1.824	1.297	>50	9	75.0	0.39
3+6	VRC06b	>50	49.799	1.531	0.023	45.013	0.087	>50	2.992	>50	>50	9.031	>50	>50	7	58.3	2.11
3+6	VRC06d	>50	>50	15.649	0.376	>50	21.1	43.15	8.436	>50	>50	>50	>50	>50	5	41.7	8.53
3+6	VRC06g	>50	>50	28.897	2.767	>50	30.834	26.925	20.072	>50	>50	>50	>50	>50	5	41.7	16.79
3+6	VRC06e	>50	>50	37.006	0.436	>50	17.704	34.013	6.907	>50	>50	>50	>50	>50	5	41.7	9.23
3+6	VRC06f	>50	>50	23.891	0.466	>50	15.085	25.7	11.089	47.14	>50	>50	>50	>50	6	50.0	11.45
3+6	VRC06	>50	>50	36.969	1.118	>50	11.193	>50	>50	>50	>50	>50	>50	>50	3	25.0	7.73
3+6	DH651.8	>50	>50	>50	44.239	>50	>50	>50	>50	>50	>50	>50	>50	>50	1	8.3	44.24
3+6	VRC06c	>50	>50	18.239	0.506	>50	12.371	26.828	8.851	>50	>50	>50	44.475	>50	6	50.0	10.32
8	VRC08e	31.58	1.773	>50	0.137	2.064	0.344	1.932	0.37	1.336	0.394	1.365	1.061	>50	11	91.7	1.10
8	VRC08c	>50	11.651	>50	0.275	18.772	1.312	0.819	1.041	2.704	5.74	>50	0.927	>50	9	75.0	2.15
8	VRC08d	40.754	0.835	>50	0.088	0.57	0.21	0.438	0.255	0.371	0.227	36.206	0.315	>50	11	91.7	0.74
8	VRC08	0.413	0.416	16.408	0.028	0.359	0.023	0.248	0.088	0.316	0.049	0.044	0.032	>50	12	100.0	0.16
1+7	DH651.3	>50	4.223	>50	0.512	1.185	2.514	7.767	1.261	1.193	0.636	3.681	1.197	>50	10	83.3	1.71
1+7	VRC02	>50	2.971	0.082	0.188	0.336	0.726	0.749	0.67	0.524	0.778	2.474	0.687	>50	11	91.7	0.60
1+7	DH651.4	>50	3.316	0.209	0.284	0.451	1.557	1	1.079	0.546	0.566	3.54	0.558	>50	11	91.7	0.80
1+7	VRC01c	>50	2.24	0.093	0.09	0.24	0.663	0.284	0.53	0.494	0.746	1.372	0.408	>50	11	91.7	0.43
1+7	DH651.2	>50	7.528	0.2	0.397	0.558	1.525	1.314	1.35	0.997	0.851	4.168	0.941	>50	11	91.7	1.11
1+7	VRC01g	>50	2.795	0.137	0.211	0.218	0.754	1.561	0.684	0.361	0.29	0.754	0.545	>50	11	91.7	0.51
1+7	VRC01b	>50	10.813	0.886	0.528	1.254	2.617	2.254	2.305	1.309	2.227	12.337	2.879	>50	11	91.7	2.29
1+7	VRC01	>50	1.261	0.035	0.069	0.121	0.326	0.144	0.287	0.205	0.288	0.562	0.359	>50	11	91.7	0.22
1+7	DH651.1	>50	6.771	0.279	0.66	0.547	2.295	2.997	2.071	1.014	0.83	3.754	0.831	>50	11	91.7	1.34
1+7	VRC01i	>50	>50	>50	0.842	0.561	>50	45.556	>50	3.823	4.352	>50	1.361	>50	6	50.0	2.81
1+7	VRC01h	>50	1.709	0.133	0.164	0.2	0.585	0.238	0.386	0.391	0.409	1.482	0.382	>50	11	91.7	0.39
1+7	VRC01f	>50	2.396	0.104	0.119	0.236	0.515	0.341	0.408	0.373	0.289	1.331	0.484	>50	11	91.7	0.39
1+7	VRC01e	19.175	2.365	0.079	0.126	0.118	0.54	0.744	0.445	0.261	0.209	0.468	0.251	>50	12	100.0	0.45
1+7	VRC01j	n/a	n/a	n/a	n/a	n/a	n/a	n/a	n/a	n/a	n/a	n/a	n/a	n/a	n/a	n/a	n/a
1+7	VRC01d	40.241	2.325	0.157	0.138	0.161	0.541	0.772	0.535	0.302	0.22	0.478	0.365	>50	12	100.0	0.55
1+7	VRC07e	>50	2.921	0.156	0.099	0.225	0.681	0.554	5.526	1.576	0.204	0.996	0.373	>50	11	91.7	0.58
1+7	VRC07f	>50	9.689	0.634	0.09	0.402	1.835	1.402	>50	2.145	0.25	1.131	0.634	>50	10	83.3	0.86
1+7	VRC07d	>50	1.801	0.142	0.051	0.189	0.507	0.487	1.998	0.581	0.097	0.177	0.149	>50	11	91.7	0.30
1+7	NIH45-46	>50	2.687	0.086	0.06	0.337	0.554	0.33	9.089	0.761	0.135	0.206	0.139	>50	11	91.7	0.38
1+7	DH651.7	>50	>50	>50	0.201	2.34	>50	>50	>50	36.307	25.933	>50	34.339	>50	5	41.7	6.86
1+7	DH651.5	>50	11.672	2.521	0.243	0.521	1.101	1.592	6.515	0.806	0.336	3.101	1.009	>50	11	91.7	1.39
1+7	DH651.6	>50	35.161	1.027	0.262	0.309	2.688	3.805	>50	4.583	0.733	5.672	1.838	>50	10	83.3	2.00
1+7	VRC07c	>50	0.945	0.023	0.088	0.222	0.351	0.275	0.51	0.469	0.32	0.399	0.178	>50	11	91.7	0.25
1+7	VRC07b	>50	1.276	0.023	0.063	0.259	0.376	0.243	0.574	0.428	0.398	0.442	0.223	>50	11	91.7	0.26
neg ctrl	Ab82	>50	>50	>50	>50	>50	>50	>50	>50	>50	>50	>50	>50	>50	0	0.0	n/a
post ctrl	CH01+CH31	8.303	0.191	0.058	0.062	0.122	0.375	0.498	0.061	0.145	0.058	1.152	0.062	>50	12	100.0	0.20

Table S3. Neutralization breadth and potency (IC_{80}, μg/ml) of VRC01 lineage antibodies. Related to Figure 5.

		IC80 ug/ml															
**Clade**	**Ab ID**	**BJOX2000**	**CE1176**	**X1632**	**X2278**	**398F1**	**25710**	**CNE8**	**TRO11**	**246F3**	**CE0217**	**CH119**	**CNE55**	**MLV-SVA**	**Positives**	**% neut**	**GeoMean**
3+6	VRC03g	>50	>50	0.111	0.023	0.505	0.711	25.185	0.338	>50	>50	10.55	1.597	>50	8	66.7	0.78
3+6	VRC03	>50	>50	0.164	0.023	0.61	0.562	>50	0.653	>50	>50	>50	3.114	>50	6	50.0	0.37
3+6	VRC03b	>50	>50	1.467	0.075	3.282	7.338	20.092	19.492	>50	>50	>50	20.876	>50	7	58.3	4.16
3+6	DH651.9	>50	>50	2.77	0.061	2.406	3.176	8.376	11.439	>50	>50	>50	48.574	>50	7	58.3	3.47
3+6	VRC03f	>50	>50	0.04	0.023	0.301	0.401	20.35	0.149	>50	>50	7.991	0.44	>50	8	66.7	0.43
3+6	VRC03e	n/a	n/a	n/a	n/a	n/a	n/a	n/a	n/a	n/a	n/a	n/a	n/a	n/a	n/a	n/a	n/a
3+6	VRC03i	>50	14.432	>50	0.076	7.9	0.436	>50	11.269	>50	>50	>50	42.864	>50	6	50.0	3.50
3+6	VRC03h	>50	24.802	0.083	0.023	0.133	0.129	44.493	0.08	>50	>50	26.99	0.291	>50	9	75.0	0.66
3+6	VRC03d	>50	>50	0.222	0.023	0.635	0.308	>50	0.181	>50	>50	>50	8.891	>50	6	50.0	0.34
3+6	VRC06b	>50	>50	>50	0.029	>50	0.345	>50	15.846	>50	>50	>50	>50	>50	3	25.0	0.54
3+6	VRC06d	>50	>50	>50	1.989	>50	>50	>50	49.759	>50	>50	>50	>50	>50	2	16.7	9.95
3+6	VRC06g	>50	>50	>50	10.896	>50	>50	>50	>50	>50	>50	>50	>50	>50	1	8.3	10.90
3+6	VRC06e	>50	>50	>50	1.511	>50	>50	>50	31.173	>50	>50	>50	>50	>50	2	16.7	6.86
3+6	VRC06f	>50	>50	>50	2.598	>50	>50	>50	47.161	>50	>50	>50	>50	>50	2	16.7	11.07
3+6	VRC06	>50	>50	>50	3.648	>50	45.597	>50	>50	>50	>50	>50	>50	>50	2	16.7	12.90
3+6	DH651.8	>50	>50	>50	>50	>50	>50	>50	>50	>50	>50	>50	>50	>50	0	0.0	n/a
3+6	VRC06c	>50	>50	>50	2.056	>50	47.322	>50	33.803	>50	>50	>50	>50	>50	3	25.0	14.87
8	VRC08e	>50	6.805	>50	0.376	10.916	1.024	6.434	1.801	4.843	1.631	7.469	3.875	>50	10	83.3	3.08
8	VRC08c	>50	>50	>50	0.693	>50	6.51	3.59	2.816	14.352	>50	>50	3.353	>50	6	50.0	3.60
8	VRC08d	>50	3.75	>50	0.273	2.254	0.804	1.596	0.85	1.145	0.798	>50	1.012	>50	9	75.0	1.10
8	VRC08	1.049	1.156	>50	0.096	1.685	0.207	0.581	0.256	0.902	0.201	0.182	0.274	>50	11	91.7	0.41
1+7	DH651.3	>50	>50	>50	1.672	4.264	9.26	43.453	4.234	3.26	2.686	10.942	2.647	>50	9	75.0	5.26
1+7	VRC02	>50	15.474	0.64	0.658	1.109	2.568	2.106	1.969	1.511	2.707	9.287	2.032	>50	11	91.7	2.20
1+7	DH651.4	>50	18.768	3.156	0.956	1.441	6.861	3.829	3.014	1.615	1.874	10.139	1.522	>50	11	91.7	3.15
1+7	VRC01c	>50	8.63	1.286	0.358	0.785	2.242	1.147	1.507	1.318	1.731	3.811	1.205	>50	11	91.7	1.55
1+7	DH651.2	>50	23.543	6.633	1.192	1.853	5.132	4.428	3.774	2.331	2.867	15.198	2.671	>50	11	91.7	4.24
1+7	VRC01g	>50	7.576	0.814	0.487	0.771	2.142	3.868	1.513	0.977	0.9	2.229	1.287	>50	11	91.7	1.48
1+7	VRC01b	>50	>50	26.282	1.933	4.466	9.419	9.171	6.368	3.594	7.456	36.239	7.342	>50	10	83.3	7.85
1+7	VRC01	>50	3.879	0.246	0.211	0.46	1.155	0.55	0.918	0.62	0.825	1.602	0.995	>50	11	91.7	0.75
1+7	DH651.1	>50	21.744	2.94	1.465	1.616	6.133	9.539	5.275	2.779	2.425	13.632	2.395	>50	11	91.7	4.34
1+7	VRC01i	>50	>50	>50	2.808	2.266	>50	>50	>50	1.385	12.322	>50	4.967	>50	5	41.7	3.52
1+7	VRC01h	>50	8.512	1.502	0.413	0.744	2.096	1.449	1.402	1.297	0.991	4.259	1.179	>50	11	91.7	1.53
1+7	VRC01f	>50	8.889	1.16	0.437	0.809	1.817	1.731	1.488	1.017	1.307	3.831	1.129	>50	11	91.7	1.52
1+7	VRC01e	>50	6.56	0.62	0.32	0.346	1.624	2.091	1.23	0.633	0.61	1.215	0.744	>50	11	91.7	0.96
1+7	VRC01j	n/a	n/a	n/a	n/a	n/a	n/a	n/a	n/a	n/a	n/a	n/a	n/a	n/a	n/a	n/a	n/a
1+7	VRC01d	>50	6.549	1.746	0.407	0.658	2.053	2.214	1.224	0.897	0.632	1.517	0.89	>50	11	91.7	1.26
1+7	VRC07e	>50	18.219	28.882	0.343	1.128	3.272	1.947	>50	5.99	1.184	3.037	1.076	>50	10	83.3	2.80
1+7	VRC07f	>50	>50	>50	0.247	1.218	5.74	10.47	>50	6.805	1.256	3.27	2.349	>50	8	66.7	2.42
1+7	VRC07d	>50	4.614	15.461	0.138	0.598	1.538	1.288	7.608	2.01	0.386	0.442	0.496	>50	11	91.7	1.28
1+7	NIH45-46	>50	6.846	31.99	0.212	0.914	2.65	1.571	>50	1.902	0.46	0.535	0.346	>50	10	83.3	1.40
1+7	DH651.7	>50	11.315	>50	0.956	13.828	>50	>50	>50	>50	>50	>50	>50	>50	3	25.0	5.31
1+7	DH651.5	>50	>50	>50	0.833	2.031	3.752	6.059	39.12	2.314	2.015	8.357	2.995	>50	9	75.0	3.83
1+7	DH651.6	>50	>50	>50	0.691	1.444	11.09	37.341	>50	19.969	2.423	17.832	6.56	>50	8	66.7	6.25
1+7	VRC07c	>50	3.804	0.14	0.262	0.964	1.331	0.795	1.445	1.371	0.62	1.175	0.652	>50	11	91.7	0.83
1+7	VRC07b	>50	4.815	0.131	0.215	0.873	1.12	0.978	2.066	1.252	1.458	1.319	0.801	>50	11	91.7	0.94
neg ctrl	Ab82	>50	>50	>50	>50	>50	>50	>50	>50	>50	>50	>50	>50	>50	0	0.0	n/a
post ctrl	CH01+CH31	>50	1.01	0.202	0.32	0.345	1.274	1.633	0.69	0.403	0.226	4.137	0.38	>50	11	91.7	0.61

a

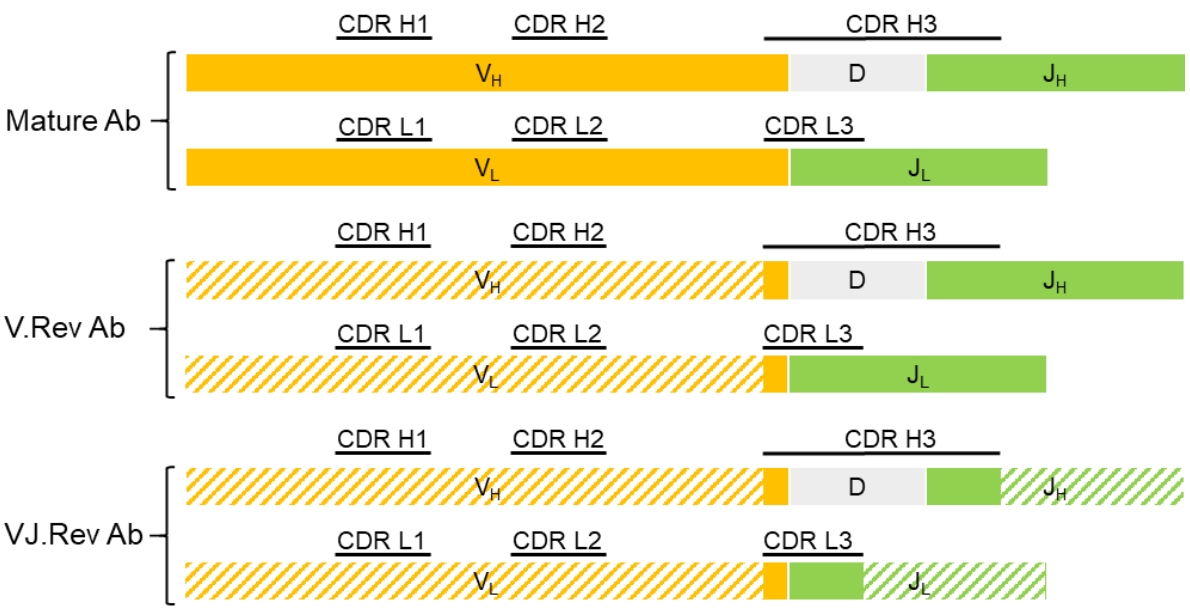

b

VRC01 bnAb V_HDJ_H rearrangement

```
--------------FR1-------------_CDR1_------FR2-----______CDR2______-----------FR3------
QVQLVQSGGQMKKPGESMRISCRASGYEFIDCTLNWIRLAPGKRPEWMGWLKPRGGAVNYARPLQGRVTMTRDVYSDTAFLELRSL
                             •  •             •  • • ••••••••••• •   • • ••

------------____CDR3____----FR4----
TVDDTAVYFCTRGKNCDYNWDFEHWGRGTPVIVSS
               ••••
```

VRC01 bnAb V_LJ_L rearrangement

```
----------FR1----------___CDR1__------FR2------__CDR2_--------------FR3---------------
EIVLTQSPGTLSLSPGETAIISCRTSQYGSLAWYQQRPGQAPRLVIYSGSTRAAGIPDRFSGSRWGPDYNLTISNLESGDFGVYYC
  •                       ••••

CDR3_-----FR4-----
QQYEFFGQGTKVQVDIKR
  •••
```

Figure S1. Schematic representation of the reversions introduced in "germline-reverted" antibodies related to the nomenclature used in this paper. Related to Figure 1. (a) The scheme shows the V(D)J rearrangements on the mature antibodies in solid color. Previously published "germline-reverted" antibodies comprise different designs in which parts of the V(D)J sequences were reverted to the unmutated sequence of the gene segment of reference (called V.Rev and VJ.Rev in this paper). The reverted parts are shown with patterned lines. (b) The V(D)J rearrangement sequences of the mature VRC01 bnAb heavy (top) and light (bottom) chains is color coded using the same scheme of panel a. Amino acids shown in black are encoded by n-nucleotide insertions. Contact sites with gp120 as described in Wu et al. 2010 are shown with solid circles as in Figure 1.

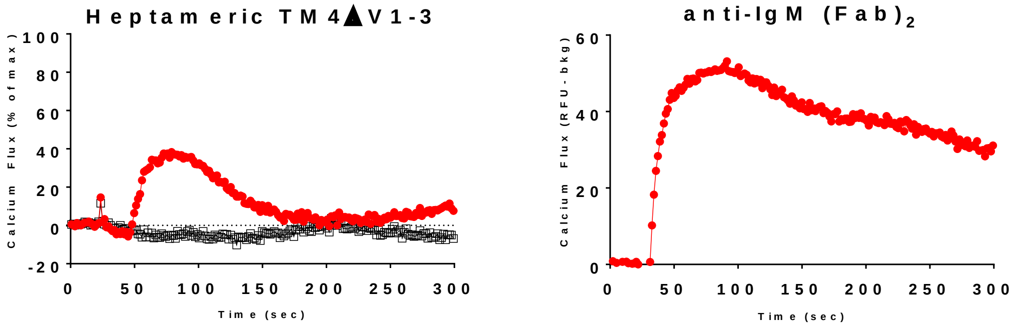

Figure S2. Effect of multimerization on TM4ΔV1-3 binding to VRC01 UCA. Related to Figure 2. Activation of Ramos B cell lines expressing the VRC01 UCA IgM BCR on cell surface was determined by measuring calcium flux upon binding. TM4ΔV1-3 core was heptamerized (left). Calcium flux is reported as % of maximum flux induced by anti-IgM $(Fab)_2$ (right). All data are representative of duplicate experiments.

a

mAb ID	CDR H3 Amino Acid Sequence	Length	Identity
VRC01 UCA	CAR--------GGYCSGGSCY-NWD---FQHW	16	
VRC01c-HuGL1	CAR---------IY-SGYDL---WW---FDPW	12	31%
VRC01c-HuGL2	CAK-----------ISG-----SYS---FDYW	9	19%
VRC01c-HuGL3	CAR----------------MY-NWNDVWFDPW	11	21%
VRC01c-HuGL4	CAR----ASRLGGY--------------FQHW	10	30%
VRC01c-HuGL5	CAK-------------------HHIRGWFDPW	8	5%
VRC01c-HuGL6	CAR--------VDY---GDYYGSWY---FDLW	14	29%
VRC01c-HuGL7	CAR-------SDGYNLG------WY---FDLW	12	29%
VRC01c-HuGL8	CAL-------SPYYDS--SGY-------FD-W	11	29%
VRC01c-HuGL9	CAR-----------DS------NWW---FDPW	8	25%
VRC01c-HuGL10	CAR--------RQYCSGGSCL--YL---FDPW	15	50%
VRC01c-HuGL11	CAS--------KVAAAGTLAK---DA--FDIW	15	18%
VRC01c-HuGL12	CAR-----------AAIAAAY------FRDPW	11	6%
VRC01c-HuGL13	CAR--------DKAVAGT----N-----FDYW	11	19%
VRC01c-HuGL14	CARRGLLGRGYSGYDRMGYYY-YYG---MDVW	24	17%
VRC01c-HuGL15	CAR--------PTEYS--SSW-YW----FDPW	13	25%
VRC01c-HuGL16	CARDHQ-----GH--S--SSW-SKR---FDYW	15	21%
VRC01c-HuGL17	CAR---------VIRS--SSS--WR---YDYW	12	19%
VRC01c-HuGL18	CAR--------VRYGSWTGYY-------FDYW	13	25%
VRC01c-HuGL19	CAR--------VPYDFWSGYY-VLS--HFDYW	17	18%
VRC01c-HuGL20	CAR--------LVGATGTS---------EDYW	11	13%
VRC01c-HuGL21	CAR--------EGRGYSTGAY-------FDYW	13	19%
VRC01c-HuGL22	CAR--------PPGPAVAGRY-NWW---FDPW	16	25%
VRC01c-HuGL23	CAR--------------GSRA-TWI---QLHW	10	25%
VRC01c-HuGL24	CAR--------VGEQLVL----N-DA--FDIW	13	19%
VRC01c-HuGL25	CAR--------DLTEVTT----------PPPW	10	0%

b

mAb ID	CDR H3 Amino Acid Sequence	Length	Identity
VRC01 UCA	CAR--GGYCSGGSCYNWDFQHW	16	
1539-B10_H	CAR--RVY------GNYDWAYW	10	19%
1539-B9_H	CAR--RRY-----NYDWEFVYW	11	25%
1540-E9_H	CAR--K--------TTMVFDYW	8	7%
1536-hvk-E	CAR--PGYGN----YGWYFDVW	12	31%
1539-A1_H	CAA--AYYNYDAESFDWYFDVW	16	19%
1539-B3_H	CAI---YYVYG----DWYFDVW	11	19%
1539-B5_H	CAV---YYGYG----DWYFDVW	11	25%
1539-C8_H	CAR-RGDYDEG----DWYFHVW	13	29%
1539-E6_H	CAR--HIFDS-----HWYFDVW	11	19%
1539-G2_H	CAR--P---STYDDYDWYFDVW	13	25%
1539-G5_H	CAK--D--MSG---TDWHLDVW	11	19%
1539-G6_H	CAR--PSYDYDH---DWYFDVW	13	19%
1538-1_H	CAR--YDNDVDG----WYFDVW	12	19%
1538-4_H	CAR--YDHDGPG----WYFDVW	12	19%
1538-12_H	CARYRNSYDDDG----WKFDIW	14	22%
1538-17_H	CAR--YDNDDDG----WSFDVW	12	19%
1538-19_H	CAR--YDNDGDG----WSFDVW	12	19%
1538-20_H	CAR--YDNDADG----WFFDVW	12	19%
1538-86_H	CAR--YDNDADG----WYFDVW	12	19%
1538-26_H	CAR--YNNEEDG----WYFDVW	12	19%
1538-65_H	CAR--YDSDGEG----WYFDVW	12	19%
1538-76_H	CAR--YDNDGEG----WYFDVW	12	19%
1538-67_H	CAR--VDYDYDGRDY-YAMDYW	15	19%
1538-69_H	CAR--DR--TGN---DWNFDVW	11	19%
1538-79_H	CAR--DR--TGN---DWYFDVW	11	19%
1538-91_H	CARELGLA--------W-FAYW	9	17%
1538-93_H	CAR--D---SSGSA--W-FAYW	10	31%

Figure S3. CDR H3 aa alignments of VRC01-class putative precursors isolated using immunogens optimized for binding to GL VRC01-class bnAbs. Related to Figure 2. CDR H3 sequences from (a) human naïve B cell-derived antibodies elicited by eOD-GT8 (Jardine et al., 2016a) or (b) induced by a step-wise immunization in V_H1-2*02/precursor VRC01 IgL knock-in mice (Tian et al., 2016) were independently aligned to VRC01 UCA using EMBOSS Water and the final alignment was manually assembled. The CAR motif preceding CDR H3 and the constant tryptophan at the end of CDR H3 are shown in gray. CDR H3 lengths are reported in aa their identity to VRC01 UCA was calculated on pairwise alignments.

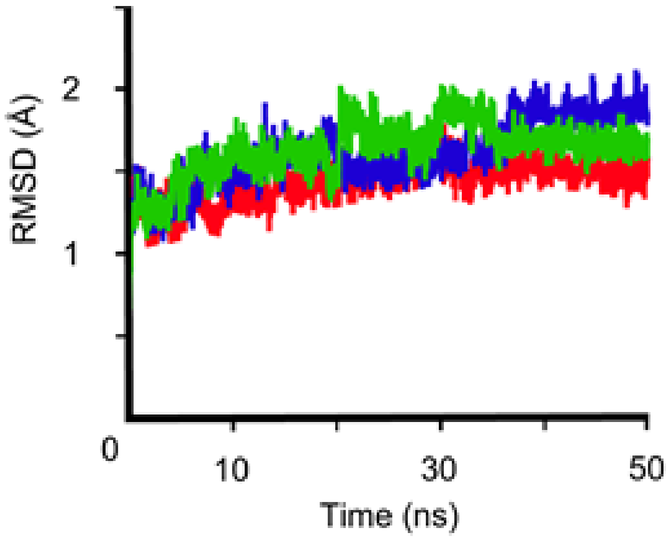

Figure S4. RMSD plot for each VRC08 CDR H3 loop during the molecular dynamics simulation. Related to Figure 6. Protein backbone RMSD of each JR-FL bound VRC08 Fab CDR H3 loop (each depicted individually in red, green, or blue) relative to the initial loop configuration during the 50 ns molecular dynamics simulation.

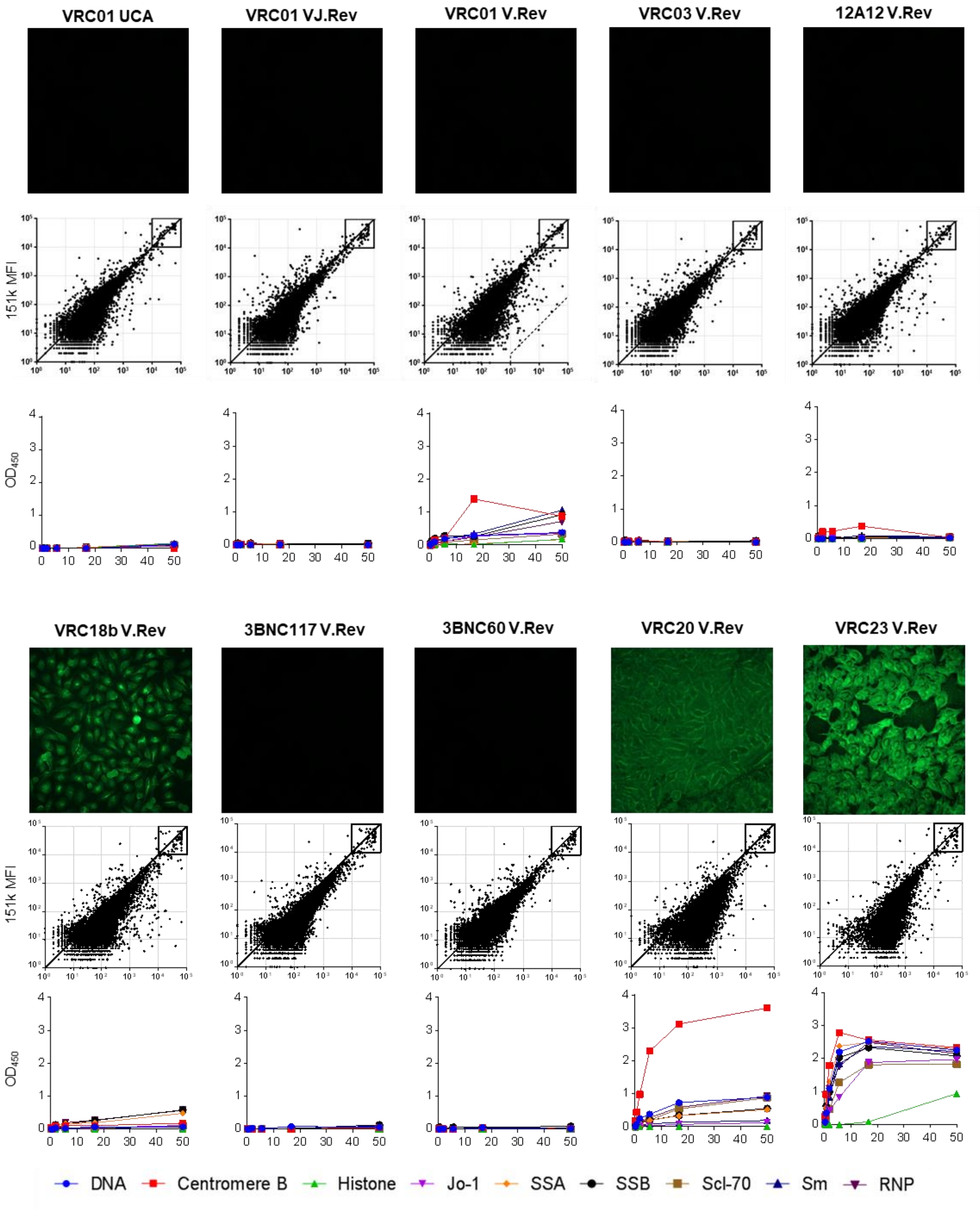

Figure S5. Auto- and polyreactivity of VRC01 UCA and VRC01-class germline-reverted antibodies. Related to Figure 7. Top: HEp-2 cell IFA staining. Middle: measurement of polyreactivity against 9,400 human antigens using the ProtoArray 5 microchip: VRC01 UCA and VRC01-class reverted mAb binding (x-axis) were compared to non-polyreactive control mAb 151k (y-axis). Significant polyreactivity is defined as 1 log more avid binding than the 151k mAb to more than 90% of the test proteins (Liu et al., 2015) and it is visualized by displacement towards the x-axis. Bottom: VRC01 UCA and VRC01-class reverted mAb binding to an ANA panel comprising 9 antigens, measured in ELISA. Results are representative of duplicate experiments and are shown background subtracted. Positivity is defined by two consecutive dilutions with OD_{450}>0.2.

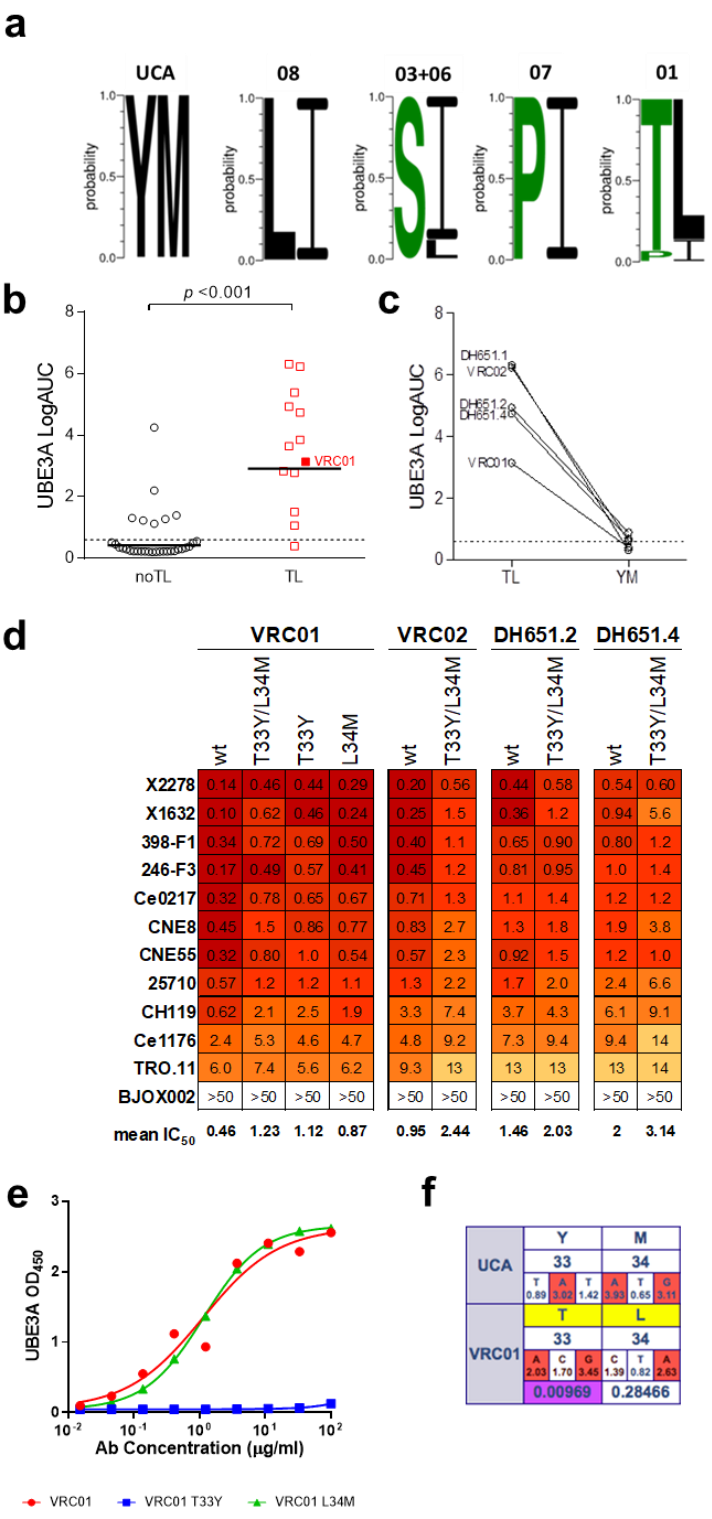

Figure S6. Reversion mutation T33Y in CDR H1 abrogates VRC01 UBE3A reactivity. Related to Figure 7. (a) Logo plot showing the frequency of mutations at positions 34 and 35 in the CDR H1 of mAbs in each clade of the VRC01 lineage. Subclades 01 and 07 are shown separately as they display distinct profiles. (b) UBE3A binding expressed as LogAUC (y-axis) of the observed VRC01 lineage mAbs is significantly associated with presence of the ^{33}TL^{34} motif. Mabs with TL mutations (n=13) are shown in clear red squares and the VRC01 bnAb is shown in solid red. Mabs without the TL mutation (n=31) are shown in clear black circles. Lines at geometric mean. Significance was evaluated with the Mann-Whitney U-test at the alpha 0.05 level. (c) Double reversion of TL to germline YM abrogates UBE3A binding of subclade 01 bnAbs VRC01, VRC02, DH651.1, DH651.2 and DH651.4. Dotted line at limit of detection. (d) Heat map analysis of neutralization of VRC01, VRC02, DH651.2 and DH651.4 bnAbs and respective single and/or double mutants against the 12-virus global panel. Neutralization potency IC_{50} is expressed in µg/ml and coloring ranges from white (>50µg/ml) to dark red (<0.023µg/ml). (e) Binding to UBE3A measured in ELISA by VRC01 bnAb (red) and single mutants VRC01 T33Y (blue) and VRC01 L34M (green). (f) Probability of the TL mutation in VRC01 bnAb CDR H1: ARMADiLLO output for VRC01 bnAb amino acids T33 and L34. Rows 1-3: VRC01 UCA aa, position and codon mutability score; Rows 4-6: VRC01 bnAb aa, position and codon mutability score; Row 7: probability of aa mutation. Colors: Blue: cold spot; Red: hot spot; Yellow: aa change; Magenta: improbable mutation (Wiehe et al., 2018).
