# Supplementary material for: Inference of the HIV-1 VRC01 Antibody Lineage Unmutated Common Ancestor Reveals Alternative Pathways to Overcome a Key Glycan Barrier
Source: Immunity. 2018 Dec 18;49(6):1162–1174.e8. doi: 10.1016/j.immuni.2018.10.015 (PMC6303191; doi:10.1016/j.immuni.2018.10.015)
Supplement: Document S2. Article plus Supplemental Information [file mmc4.pdf]

Immunity

Article

Inference of the HIV-1 VRC01 Antibody Lineage Unmutated Common Ancestor Reveals Alternative Pathways to Overcome a Key Glycan Barrier

Graphical Abstract

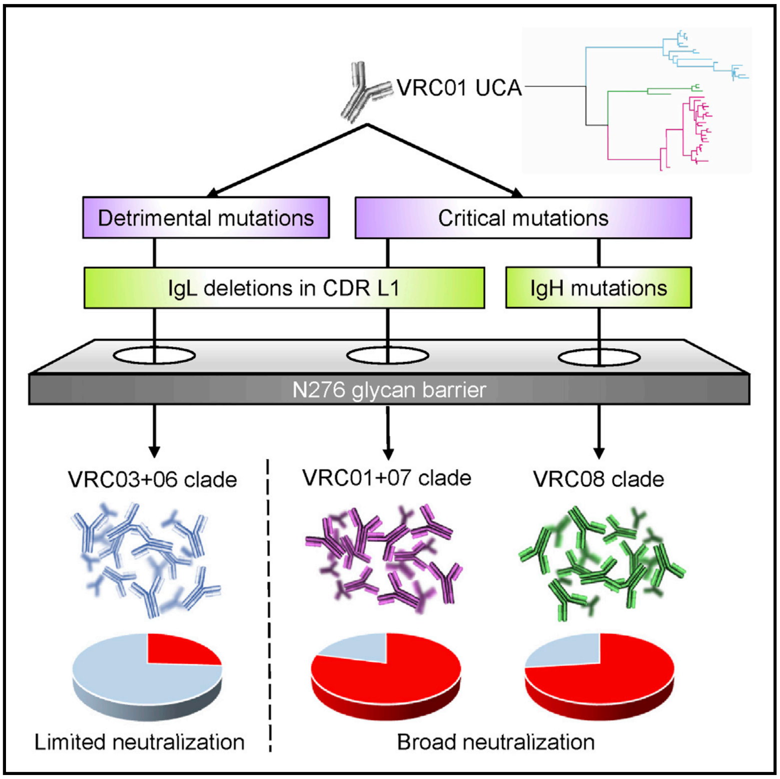

Highlights

- A high-probability VRC01 lineage UCA was inferred and CDRH3 evolution defined
- Env immunogens bind to VRC01 UCA with affinity sufficient to activate naive B cells
- Early mutations defined maturation pathways toward limited or broad neutralization
- Antibodies with long CDRH3s achieved neutralization breadth without shortening CDRL1s

Authors

Mattia Bonsignori, Eric Scott, Kevin Wiehe, ..., Kevin O. Saunders, Thomas B. Kepler, Barton F. Haynes

Correspondence

mattia.bonsignori@duke.edu (M.B.),
barton.haynes@duke.edu (B.F.H.)

In Brief

Understanding how HIV-1 VRC01-class broadly neutralizing antibodies overcome maturation barriers is key for vaccine development. Bonsignori et al. inferred the unmutated common ancestor of the VRC01 lineage, reconstructed the stages of lineage maturation, and identified multiple solutions adopted by evolving B cells to overcome the N276 glycan barrier and achieve broad neutralization.

Bonsignori et al., 2018, Immunity *49*, 1162–1174
December 18, 2018 © 2018 The Authors. Published by Elsevier Inc.
https://doi.org/10.1016/j.immuni.2018.10.015

CellPress

Immunity
Article

Inference of the HIV-1 VRC01 Antibody Lineage Unmutated Common Ancestor Reveals Alternative Pathways to Overcome a Key Glycan Barrier

Mattia Bonsignori,[1,2,15,17,*] Eric Scott,[1,15] Kevin Wiehe,[1,2,15] David Easterhoff,[1,2] S. Munir Alam,[1,2] Kwan-Ki Hwang,[1] Melissa Cooper,[1] Shi-Mao Xia,[1] Ruijun Zhang,[1,13] David C. Montefiori,[1,3] Rory Henderson,[1] Xiaoyan Nie,[4] Garnett Kelsoe,[1,4] M. Anthony Moody,[1,5] Xuejun Chen,[6] M. Gordon Joyce,[6,14] Peter D. Kwong,[6] Mark Connors,[7] John R. Mascola,[6] Andrew T. McGuire,[8,9] Leonidas Stamatatos,[8,9] Max Medina-Ramírez,[10] Rogier W. Sanders,[10,11] Kevin O. Saunders,[1,3] Thomas B. Kepler,[12,16] and Barton F. Haynes[1,2,4,16,*]

[1]Duke Human Vaccine Institute, Duke University, Durham, NC, USA
[2]Department of Medicine, Duke University, Durham, NC, USA
[3]Department of Surgery, Duke University, Durham, NC, USA
[4]Department of Immunology, Duke University, Durham, NC, USA
[5]Department of Pediatrics, Duke University, Durham, NC, USA
[6]Vaccine Research Center, National Institute of Allergy and Infectious Diseases, National Institutes of Health, Bethesda, MD, USA
[7]Laboratory of Immunoregulation, National Institute of Allergy and Infectious Diseases, National Institutes of Health, Bethesda, MD, USA
[8]Vaccine and Infectious Disease Division, Fred Hutchinson Cancer Research Center, Seattle, WA, USA
[9]Department of Global Health, University of Washington, Seattle, WA, USA
[10]Department of Medical Microbiology, Academic Medical Center, University of Amsterdam, Amsterdam, the Netherlands
[11]Department of Microbiology and Immunology, Weill Medical College of Cornell University, New York, NY, USA
[12]Department of Microbiology, Boston University School of Medicine, Boston, MA, USA
[13]Present address: Sanofi, Framingham, MA, USA
[14]Present address: U.S. Military HIV Research Program, Walter Reed Army Institute of Research, Silver Spring, MD, USA; Henry M. Jackson Foundation for the Advancement of Military Medicine, Inc., Bethesda, MD, USA
[15]These authors contributed equally
[16]Co-senior author
[17]Lead Contact
*Correspondence: mattia.bonsignori@duke.edu (M.B.), barton.haynes@duke.edu (B.F.H.)
https://doi.org/10.1016/j.immuni.2018.10.015

SUMMARY

Elicitation of VRC01-class broadly neutralizing antibodies (bnAbs) is an appealing approach for a preventative HIV-1 vaccine. Despite extensive investigations, strategies to induce VRC01-class bnAbs and overcome the barrier posed by the envelope N276 glycan have not been successful. Here, we inferred a high-probability unmutated common ancestor (UCA) of the VRC01 lineage and reconstructed the stages of lineage maturation. Env immunogens designed on reverted VRC01-class bnAbs bound to VRC01 UCA with affinity sufficient to activate naive B cells. Early mutations defined maturation pathways toward limited or broad neutralization, suggesting that focusing the immune response is likely required to steer B cell maturation toward the development of neutralization breadth. Finally, VRC01 lineage bnAbs with long CDR H3s overcame the HIV-1 N276 glycan barrier without shortening their CDR L1, revealing a solution for broad neutralization in which the heavy chain, not CDR L1, is the determinant to accommodate the N276 glycan.

INTRODUCTION

The development of a preventative HIV-1 vaccine is a global health priority. Among the known sites of vulnerability of the HIV-1 envelope glycoprotein (Env), the CD4-binding site (CD4bs) is an appealing target because the requirement for receptor engagement with CD4 limits its variability. VRC01 is a potent CD4bs broadly neutralizing antibody (bnAb) and is the prototype for the VRC01-class of bnAbs (Scheid et al., 2011; Wu et al., 2010, 2011, 2015; Zhou et al., 2013, 2015). VRC01-class bnAbs protect animals from experimental HIV-1 or SHIV challenge and can transiently reduce plasma viremia in both non-human primates and chronically HIV-1-infected individuals (Balazs et al., 2014; Barouch et al., 2013; Caskey et al., 2015; Lynch et al., 2015; Pegu et al., 2014; Pietzsch et al., 2012; Rudicell et al., 2014; Shingai et al., 2014). Despite an extraordinary accumulation of somatic mutations, VRC01-class bnAbs adopt similar structures and retain a similar angle of approach to engage the CD4bs (Diskin et al., 2011; Klein et al., 2013; Scharf et al., 2013; Scheid et al., 2011; Wu et al., 2011; Zhou et al., 2010, 2013, 2015). Moreover, V_H allele usage of VRC01-class bnAbs is restricted to V_H1-2*02, and light chains are restricted to unusually short complementarity-determining region (CDR) L3s of 5 amino acids (aa) (Jardine et al., 2013; West et al., 2012; Zhou et al., 2013). These predictable characteristics set boundaries useful to guide immunogen design, making the VRC01-bnAb class a particularly attractive target.

 © 2018 The Authors. Published by Elsevier Inc.
This is an open access article under the CC BY license (http://creativecommons.org/licenses/by/4.0/).

The first step to initiate bnAb lineage maturation is to engage naive B cells expressing the unmutated IgH and IgL precursor, referred to as the "unmutated common ancestor" (UCA) of the lineage (Haynes et al., 2012; Kepler et al., 2014). The high levels of somatic hypermutation (SHM) and of insertions and deletions (indels) among the observed members of the VRC01 clone have posed a significant challenge to accurately infer the VRC01 lineage UCA. To design immunogens that engage VRC01-class bnAb unmutated precursors, the field has instead used "germline-reverted" (GL) versions of individual VRC01-class bnAbs in which either the IgH and IgL V gene segments or both the V and J gene segments were reverted to the respective templated germline sequences. Here, we will refer to these GL mAbs as "V.Rev" and "VJ.Rev," respectively. Practically, the GL versions of VRC01-class bnAbs retain the somatically mutated CDR H3s of the mature bnAb of reference. However, the importance of an accurate inference of the CDR H3 is underscored by recent findings indicating that, among VRC01-class bnAb precursors, the CDR H3 plays a more predominant role in HIV-1 gp120 envelope glycoprotein (Env) recognition than once thought (Yacoob et al., 2016). Nonetheless, this approach has yielded the design of multiple immunogens that bind to GL VRC01-class monoclonal antibodies (mAbs). Gp120 Env outer domain proteins (i.e., eOD-GT6 and eOD-GT8) activate putative VRC01-class B cell precursors in transgenic mice and have been used to isolate putative VRC01 naive B cell precursors from HIV-1-uninfected human subjects (Havenar-Daughton et al., 2018; Jardine et al., 2013, 2015, 2016a; Sok et al., 2016; Tian et al., 2016). HIV-1 426c Env-derived core proteins, in which the variable loops 1, 2, and 3 were deleted (Dosenovic et al., 2015; McGuire et al., 2016), as well as a stabilized BG505 Env-derived SOSIP v4.1-GT1 trimer (referred to as "GT1 trimer" in this paper) (Medina-Ramírez et al., 2017) activate germline-reverted VRC01 B cells in knock-in mice.

For all these immunogens, removal of three glycans that partially occlude the CD4bs was necessary to confer binding to the GL VRC01-class mAbs (Jardine et al., 2013; McGuire et al., 2013; Medina-Ramírez et al., 2017). However, the glycan at position N276 in Env D Loop is present in ~95% of circulating HIV-1 strains and most VRC01-class bnAbs evolved to accommodate the N276 glycan by shortening the germline-encoded CDR L1. Vaccination strategies using progressively glycosylated immunogens have succeeded in eliciting serum antibody responses that can neutralize viruses lacking the N276 glycan site, but not those with the N276 glycan site present (Briney et al., 2016; Tian et al., 2016). Hence, the genetic determinants and biological roadblocks hindering the induction of VRC01-class bnAbs that can accommodate the glycan at N276 remain imprecisely defined, suggesting that a detailed VRC01 B cell lineage genealogy is necessary to inform the design of immunogens that will guide lineage maturation toward full neutralization breadth. By inferring the UCA of bnAb lineages with high probability, antibody maturation pathways can be defined that identify biological barriers to the maturation of these lineages toward neutralization breadth. In turn, these analyses can inform immunogen design and vaccination strategies to overcome such barriers (Bonsignori et al., 2016, 2017). The challenge of inferring the UCA and maturation intermediate antibodies (IA) of a lineage can be resolved with high probability with a sufficiently large set of clonally related natural heavy (IgH) and light (IgL) chain-paired sequences. Here, we assembled a 45 naturally paired IgH+IgL VRC01 lineage mAbs to reconstruct the VRC01 lineage genealogy, to infer the VRC01 UCA and to identify two different pathways and mechanisms whereby accommodation of the N276 glycan led to broad neutralization.

RESULTS

Inference of the VRC01 UCA

To infer the UCA of the VRC01 lineage, we used 45 naturally paired IgH+IgL VRC01 lineage mAbs isolated from memory B cells of NIH donor 45, from whom the VRC01 bnAb was isolated (Wu et al., 2010). Of these, 36 were previously described (Li et al., 2012; Scheid et al., 2011; Wu et al., 2010, 2015) and 9 (DH651.1 through DH651.9) were newly isolated.

The observed members of the clone displayed high levels of SHM and multiple indels, making the inference of the VRC01 UCA substantially more difficult than that of a more typical clone of the same size. The methods available for algorithmic inference of the UCA rely on simplifying assumptions in several components, including the inference of multiple sequence alignment and phylogenetic trees, among others. The lack of appropriate models for insertions and deletions (indels), in particular, made a purely algorithmically computed inference unreliable in the context of the VRC01 lineage. Hence, we combined multiple algorithmic methods and human judgment where it was practically impossible to encode the requisite information into a statistical model. This hybrid method used to infer the VRC01 UCA is described in the STAR Methods.

The IgH and IgL sequences of the new VRC01 UCA are shown in Figure 1A. The sum of minimum expected errors over all nucleotides (nt) in the VRC01 UCA V(D)J rearrangements was estimated to be 3.4 nt for IgH and 1.2 nt for IgL. Thus, within the limits associated with any kind of inference, the VRC01 UCA offers an accurate estimate of the unmutated CDR H3 and its evolution through SHM toward the 45 observed mAbs in the lineage.

VRC01 UCA CDR H3 and IgL Differ from Those of GL VRC01 Lineage Antibodies Used to Design Immunogens that Target VRC01-Class bnAb Precursors

We aligned VRC01 UCA to five GL VRC01 lineage mAbs (Figure 1B; Jardine et al., 2016a; Wu et al., 2010). The CDR H3 of the VRC01 UCA was 16 amino acid (aa) long, whereas the length of the V.Rev and VJ.Rev VRC01 lineage mAbs CDR H3s varies from 12 to 16 aa, which reflect indels in the mature mAbs used as reference (Figures 1C and S1). CDR H3 aa identities to VRC01 UCA ranged from 19% (VRC06 VJ.Rev) to 63% (NIH45-46 VJ.Rev) (Figure 1C). The CDR H3 of VRC01 UCA contained two cysteines: Cys98 and Cys100c. Since the arrangement of disulfide bonds can affect the CDR H3 conformation and the stability of VRC01 lineage mAbs (Jardine et al., 2015; Wu et al., 2015), we compared the positions of cysteines in the VRC01 UCA and the GL mAbs (Figure 1C). Cys98 is a signature of the VRC01 lineage (Wu et al., 2015) and was conserved in all GL mAbs except VRC01 VJ.Rev. Conversely, Cys100c was not preserved in any of the GL mAbs and it was replaced by an aspartic acid or, in NIH45-46 VJ.Rev, by a serine (Figure 1C). In the mature VRC01 and NIH45-46 bnAbs, Cys98 forms a disulfide bond with Cys32 (PDB: 3NGB, 3U7W) (Diskin et al., 2011; Wu et al., 2010), which

A

VRC01 UCA heavy chain

1	2	3	4	5	6	7	8	9	10	11	12	13	14	15	16	17	18	19	20	21	22	23	24	25
Q	V	Q	L	V	Q	S	G	A	E	V	K	K	P	G	A	S	V	K	V	S	C	K	A	S

26	27	28	29	30	31	32	33	34	35	36	37	38	39	40	41	42	43	44	45	46	47	48	49	50
G	Y	T	F	T	G	Y	Y	M	H	W	V	R	Q	A	P	G	Q	G	L	E	W	M	G	W

51	52	52A	53	54	55	56	57	58	59	60	61	62	63	64	65	66	67	68	69	70	71	72	73	74
I	N	P	N	S	G	G	T	N	Y	A	Q	K	F	Q	G	R	V	T	M	T	R	D	T	S

75	76	77	78	79	80	81	82	82A	82B	82C	83	84	85	86	87	88	89	90	91	92	93	94	95	96
I	S	T	A	Y	M	E	L	S	R	L	R	S	D	D	T	A	V	Y	Y	C	A	R	G	G

97	98	99	100	100A	100B	100C	100D	100E	100F	100G	100H	101	102	103	104	105	106	107	108	109	110	111	112	113
Y	C	S	G	G	S	C	Y	N	W	D	F	Q	H	W	G	Q	G	T	L	V	T	V	S	S

VRC01 UCA light chain

1	2	3	4	5	6	7	8	9	10	11	12	13	14	15	16	17	18	19	20	21	22	23	24	25
E	I	V	L	T	Q	S	P	G	T	L	S	L	S	P	G	E	R	A	T	L	S	C	R	A

26	27	27A	28	29	30	31	32	33	34	35	36	37	38	39	40	41	42	43	44	45	46	47	48	49
S	Q	S	V	S	S	S	Y	L	A	W	Y	Q	Q	K	P	G	Q	A	P	R	L	L	I	Y

50	51	52	53	54	55	56	57	58	59	60	61	62	63	64	65	66	67	68	69	70	71	72	73	74
G	A	S	S	R	A	T	G	I	P	D	R	F	S	G	S	G	S	G	T	D	F	T	L	T

75	76	77	78	79	80	81	82	83	84	85	86	87	88	89	90	91	92	93	94	95	96	97	98	99
I	S	R	L	E	P	E	D	F	A	V	Y	Y	C	Q	Q	Y	-	-	-	-	E	F	F	G

100	101	102	103	104	105	106	107
Q	G	T	K	L	E	I	K

B

```
HEAVY CHAINS        ---------------FR1-------------- _CDR1_------FR2------ ______CDR2_______-----
                    ....|....| ....|....| ....|....| ....|....| ....|....| .....|....| ....|....|
                             10        20        30        40        50  a       60         70
VRC01 UCA           QVQLVQSGAE VKKPGASVKV SCKASGYTFT GYYMHWVRQA PGQGLEWMGW INPNSGGTNYA QKFQGRVTMT
VRC01 V.Rev         .......... .......... .......... .......... .......... ........... ..........
VRC01 VJ.Rev        .......... .......... .......... .......... .......... ........... ..........
VRC03 VJ.Rev        .......... .......... .......... .......... .......... ........... ..........
VRC06 VJ.Rev        .......... .......... .......... .......... .......... ........... ..........
NIH45-46 VJ.Rev     .......... .......... .......... .......... .......... ........... ..........
Mature VRC01        ........GQ M....E.MRI ..R....E.I DCTLN.I.L. ..KRP..... LK.RG.AV... RPL.......
                                                   ●   ●              ●  ●  ● ●●●●●●●● ●●● ●   ●

                    -----------FR3--------------_______CDR3_______----FR4----
                    ....|....| .......| ....|....
                             80  abc         90
                                                                                 Ref.
VRC01 UCA           RDTSISTAYM ELSRLRSD DTAVYYCARG G-YCSGGSCYNWDFQHWGQGTLVTVSS   this paper
VRC01 V.Rev         .......... ........ .......... K-N.----D.....E...R..P.I...   Wu et al. Science 2010
VRC01 VJ.Rev        .......... ........ .......... K-NS----D..................   Jardine et al. Science 2016
VRC03 VJ.Rev        .......... ........ .........R .-S.DYCGDF--PW.............   Jardine et al. Science 2016
VRC06 VJ.Rev        .......... ........ .........R .SS.PHCGDF--H.DP...........   Jardine et al. Science 2016
NIH45-46 VJ.Rev     .......... ........ .......... .-..T..VS...Y.DL..R........   Jardine et al. Science 2016
Mature VRC01        ..VYSD..FL ..RS.TV. .....F.T.. K-N.----D.....E...R..P.I...   Wu et al. Science 2010
                    ● ●●                                   ●●●●


LIGHT CHAINS        -----------FR1-----------_____CDR1____-------FR2------__CDR2__-------FR3-----
                    ....|....| ....|....| ....|.....| ....|....| ....|....| ....|....| ....|....|
                             10        20        a  30        40        50        60         70
VRC01 UCA           EIVLTQSPGT LSLSPGERAT LSCRASQSVSS SYLAWYQQKP GQAPRLLIYG ASSRATGIPD RFSGSGSGTD
VRC01 VJ.Rev        ........A. .......... ........... -......... .........D ..N......A ..........
VRC01 VJ.Rev        ........A. .......... ........... -......... .........D ..N......A ..........
VRC03 VJ.Rev        .......... .......... ........... .......... .......... .......... ..........
VRC06 VJ.Rev        ........A. .......... ........... .......... .........D .......... ..........
NIH45-46 VJ.Rev     ...M....A. ..V....... ........... -N........ .......... ..T......A .........E
Mature VRC01        .......... .......T.I I...T..YG.- --......R. ......V..S G.T..A.... .....RW.P.
                      ●                         ●●●●

                    ------------------_CDR3_----FR4---
                    ....|....| ....|....|
                             80        90
                                                    Ref.
VRC01 UCA           FTLTISRLEP EDFAVYYCQQ YEFFGQGTKLEIK this paper
VRC01 VJ.Rev        ......S... .......... .........VQVD Wu et al. Science 2010
VRC01 VJ.Rev        ......S... .......... ............. Jardine et al. Science 2016
VRC03 VJ.Rev        .......... .......... F............ Jardine et al. Science 2016
VRC06 VJ.Rev        .......... .......... F............ Jardine et al. Science 2016
NIH45-46 VJ.Rev     ......S.QS .......... ............. Jardine et al. Science 2016
Mature VRC01        YN....N..S G..G...... .........VQVD Wu et al. Science 2010
                                          ●●●
```

C

	CDR H3 (95 | abcdefgh | 102)	CDR H3 Length	Indels	AA identity to VRC01 UCA	
**VRC01 UCA**	GG-YCSGGSCYNWDFQH	16			
**VRC01 V.Rev**	.K-N.----D.....E.	12	-4	8/16	50%
**VRC01 VJ.Rev**	.K-NS----D.......	12	-4	8/16	50%
**VRC03 VJ.Rev**	R.-S.DYCGDF--PW..	14	-2	4/16	25%
**VRC06 VJ.Rev**	R.SS.PHCGDF--H.DP	15	+1,-2	3/16	19%
**NIH45-46 VJ.Rev**	..-..T..VS...Y.DL	16	0	10/16	63%

Figure 1. VRC01 UCA IgH and IgL Amino Acid Sequences Differed from Those of VRC01 Lineage GL Antibodies

(A) VRC01 UCA IgH (top) and IgL (bottom) aa sequences numbered according to the Kabat system (Kabat et al., 1991).

(B) Ig heavy (top) and light (bottom) chain alignment of VRC01 UCA to VRC01 lineage GL mAbs. The sequence of the mature VRC01 bnAb is shown as reference and mature VRC01 residues involved in interactions with gp120 Env (Zhou et al., 2010) are indicated with closed circle.

(C) VRC01 UCA CDR H3 aa sequence (positions 95 through 102) aligned to CDR H3s of VRC01 lineage GL mAbs. Cysteines shown in green shade. Differences in CDR H3 length, indels, and aa identity to VRC01 UCA are shown on the right.

See also Figure S1.

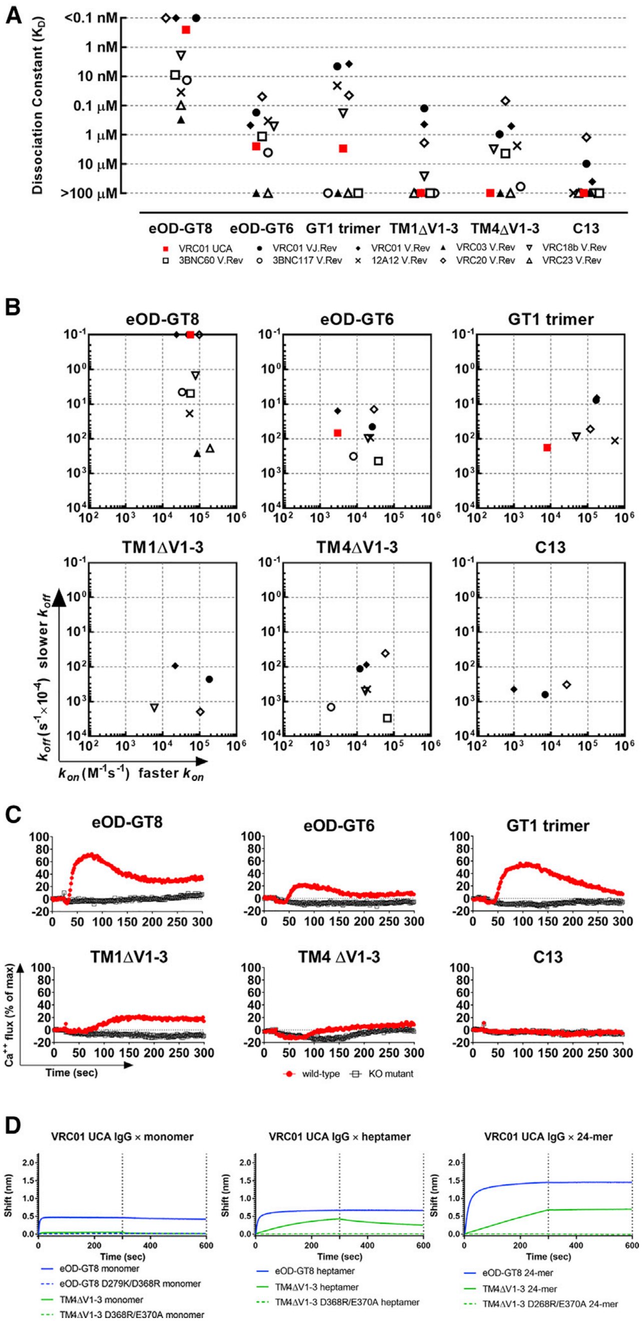

Figure 2. VRC01 UCA Bound to Immunogens Designed of GL VRC01-Class mAbs

(A) VRC01 UCA (red) and GL VRC01-class mAbs (black) dissociation constants against six immunogens. Each data point shows the average of at least duplicate experiments. mAbs that did not display measurable binding are shown with K_D > 100 μM.

(B) On-rates (x-axis) and off-rates (y-axis) for the same mAbs and immunogens. Only mAbs with K_D < 100 μM are shown.

(C) B cell activation mediated by immunogens (red) measured by calcium flux (y-axis) on Ramos B cells expressing VRC01 UCA IgM BCR over 300 s (x-axis). Immunogen mutants with either D279K/D368R or D268R/E370A double mutation are shown in black ("KO mutants"). For the GT1 trimer, BG505 SOSIP was used as KO mutant. Results are expressed as percentage of the maximum signal obtained with an anti-IgM F(ab)$_2$ antibody and are representative of at least duplicate experiments.

(D) Binding of monomeric (left), heptameric (middle), and icositetrameric (right) TM4ΔV1-3 core (green) and eOD-GT8 (blue) to VRC01 UCA IgG. KO mutants are shown with dotted lines. Results are representative of duplicate experiments.

See also Table S1, Figures S2 and S3.

is the result of a mutation in CDR H1 and therefore not present in the VRC01 VJ.Rev and NIH45-46 VJ.Rev mAbs. In VRC01 VJ.Rev, Cys98 has been intentionally mutated to Ser to remove the unpaired cysteine and stabilize the antibody (Jardine et al., 2015) whereas in NIH45-46 VJ.Rev, Cys98 remains unpaired (PDB: 4JDV and 5IGX) (Scharf et al., 2013). In VRC03 VJ.Rev and VRC06 VJ.Rev, Cys98 forms an intra-CDR H3 disulfide bond with Cys100a (PDB: 5JOF) (Davenport et al., 2016), which was introduced by the G100aC mutation in the mature VRC03 and VRC06 mAbs and, as in the mature mAbs (PDB: 3SE8, 5JXA, and 4JB9) (Davenport et al., 2016; Georgiev et al., 2013; Wu et al., 2011), stabilizes a β-turn at the apex of the CDR H3 loop.

This analysis demonstrated that the Cys98 and Cys100c arrangement of the VRC01 UCA differed from that of all the GL mAbs, which retained the somatically mutated CDR H3s of the mature bnAb of reference. Thus, the observed inter- and intra-CDR H3 disulfide bond shuffling from the VRC01 UCA to the mature VRC01 lineage bnAbs suggested an additional hurdle to the development of breadth.

The VRC01 UCA IgL Vk gene segment sequence differed from the GL mAbs due to variations in the Vk gene segments used as templates for each reversion. The original VRC01 bnAb Vk gene segment assignment was Vk3-11*01 and was later revised to Vk3-20*01 (Wu et al., 2010; Zhou et al., 2013). Our genealogy analysis supported this later assignment. VRC03 VJ.Rev and VRC06 VJ.Rev were reverted to Vk3-20*01 whereas VRC01 VJ.Rev and VRC01 V.Rev were reverted to Vk3-11*01. NIH45-46 VJ.Rev was instead reverted to Vk3-15*01 (Jardine et al., 2016a). The GL mAbs not templated on Vk3-20*01 lack Ser31 and, consequently, have shorter CDR L1s. Overall, the light chains of the GL mAbs were more similar to the VRC01 UCA than the heavy chains (Figure 1B).

These data highlighted the differences between the CDR H3s and light chains of VRC01 UCA and the VRC01 bnAb lineage-derived GL mAbs.

VRC01 UCA Binds to Germline-Targeting Env Forms

Immunogens intended to target VRC01-class bnAb precursors in humans have been designed using VRC01 class GL mAbs to optimize their reactivity and include eOD-GT6 and eOD-GT8 outer domain proteins (Jardine et al., 2013, 2016a), 426c gp120 Env-derived core proteins TM1ΔV1-3 (also reported as 426c degly3) and TM4ΔV1-3 cores (McGuire et al., 2013, 2016), C13 gp120 core (Tian et al., 2016), and the GT1 trimer (Medina-Ramírez et al., 2017). To assess the ability of these six Env immunogens to bind to VRC01 UCA, we measured the binding kinetics of VRC01 UCA and compared it to nine GL VRC01-class mAbs derived from VRC01-class bnAbs (isolated from multiple individuals) (Table S1). eOD-GT8 and eOD-GT6 bound to VRC01 UCA with apparent dissociation constants (K_D) of <0.1 nM and 2.4 μM, respectively (Figure 2A). eOD-GT8 had the most favorable apparent affinity for all the mAb tested (K_D range: <0.1 nM–0.3 μM) and displayed strong affinity for

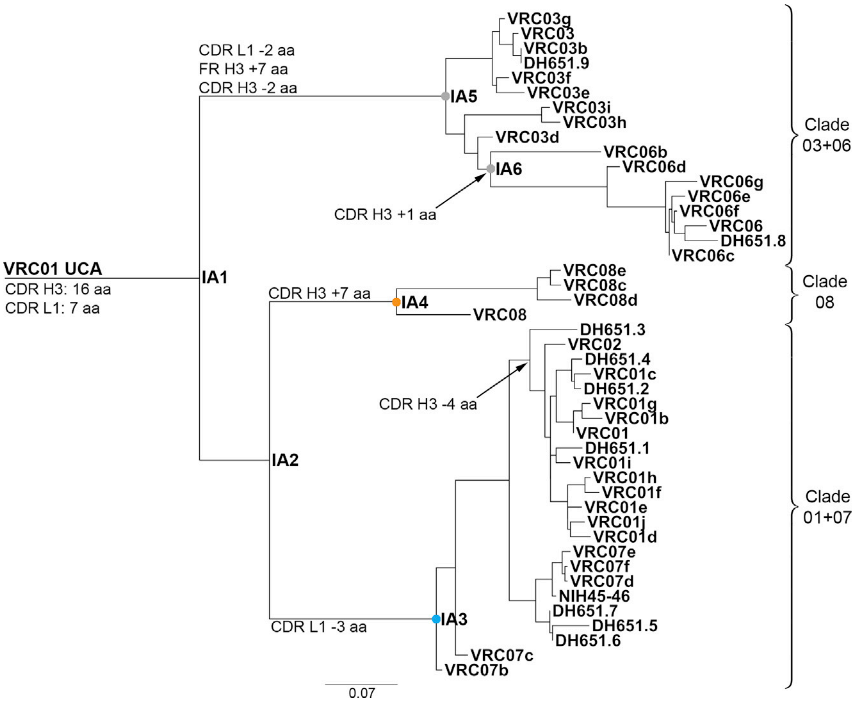

Figure 3. VRC01 Phylogeny from the VRC01 UCA Detailed the Maturation Pathways of VRC01 Lineage Antibodies

VRC01 phylogeny reconstructed from 45 mAbs with natural paired IgH and IgL sequences. Inferred maturation intermediate antibodies IA1 through IA6 are colored based on clade membership (right). Indels (aa) are mapped on the tree. Units of branch-length estimates are nt substitutions per site.

VRC01 UCA (K_D = 0.25 nM). eOD-GT6 bound with 10,000-fold weaker affinity (K_D = 2.5 μM). The GT1 trimer bound to 5 of 9 GL mAbs (apparent K_D range: 3.7 nM–0.18 μM) and also bound to VRC01 UCA, albeit with much weaker affinity than eOD-GT8 (apparent K_D = 3 μM). While the GT1 trimer apparent affinity to VRC01 UCA was comparable to that of eOD-GT6, it was likely overestimated due to the potential avidity effect of the trimeric configuration of GT1. TM1ΔV1-3, TM4ΔV1-3, and C13 core proteins bound to four, seven, and three GL mAbs, respectively, but did not bind to VRC01 UCA (Figure 2A).

Differences in eOD-GT8 apparent affinities were mainly driven by variations in off-rates (k_{off}), which spanned 3 orders of magnitude (k_{off} range: < 0.01–25.92 ms^{-1}) (Figure 2B). The fast on-rate (k_{on}) and slow k_{off} of eOD-GT8 for VRC01 UCA indicated that eOD-GT8 not only optimized the complementarity for the cognate antigen but also stabilized the complex. eOD-GT6 displayed slower k_{on} and faster k_{off} than eOD-GT8 for VRC01 UCA and bound to most of the GL mAbs better than to VRC01 UCA. The GT1 trimer on- and off-rates for VRC01 UCA were faster than eOD-GT6 (k_{on} = 8,000 $M^{-1}s^{-1}$ versus 3,000 $M^{-1}s^{-1}$; k_{off} = 0.018 s^{-1} versus 0.068 s^{-1}, respectively), suggesting better GT1 trimer complementarity for VRC01 UCA but a less stable GT1:VRC01 UCA complex than eOD-GT6, despite the more favorable avidity effect of its trimeric conformation (Figure 2B).

Since the first step to elicit bnAbs is to activate naive B cell precursors by engaging the IgM B cell receptor (BCR), we constructed a Ramos B cell line expressing transmembrane VRC01 UCA IgM on the cell surface and measured the ability of the immunogens to mediate Ca^{2+} flux. All Env forms were biotinylated and tetramerized on streptavidin to optimize BCR cross-linking (Ota et al., 2012). eOD-GT8 displayed superior activation of VRC01 UCA IgM BCR-expressing B cells (peak % response of maximum αIgM binding: 72.3%), which was >3-fold higher than that of eOD-GT6 (peak response: 22.2%) (Figure 2C). As a multimer, the GT1 trimer activated B cells more efficiently than the tetrameric form of eOD-GT6 (peak response: 56.2%), possibly because of a combination of faster on-rate and higher degree of multimerization ("tetramer of trimers") (Figure 2C). The tetramerized TM1ΔV1-3 activated IgM BCR-expressing B cells (peak response: 22.4%) despite not binding to VRC01 UCA IgG in monomeric form, whereas C13 core proteins and tetrameric TM4ΔV1-3 did not induce Ca^{2+} flux (Figure 2C). Further multimerization improved eOD-GT8 binding to VRC01 UCA IgG and conferred upon TM4ΔV1-3 binding to VRC01 UCA IgG (Figure 2D). Moreover, heptamerization of TM4ΔV1-3 was sufficient to induce activation of VRC01 UCA IgM BCR-expressing B cells (peak response: 38.2%) (Figure S2).

Thus, multimerized eOD-GT8, GT1 trimer, TM1ΔV1-3, and TM4ΔV1-3 activated Ramos cells expressing VRC01 UCA IgM BCR. The different kinetics between VRC01 UCA and the GL mAbs highlighted the impact of CDR H3 on the recognition of VRC01 class naive precursors. Overall, the Env outer domain proteins (eOD-GT6, eOD-GT8) were less sensitive to differences in CDR H3. In fact, human naive B cells isolated with eOD-GT8 yielded mAbs with a variety of CDR H3 lengths and aa compositions (Jardine et al., 2016a) that substantially differed from VRC01 UCA, with the closest Ab reaching only 50% identity to the VRC01 UCA CDR H3 (Figure S3). Also, CDR H3 length has only a modest impact on naive VRC01-class B cells affinity for eOD-GT8 (Havenar-Daughton et al., 2018). Similarly, stepwise immunization with eOD-GT6, progressively glycosylated 426c core proteins, and 426c-WT SOSIP of ES cell mice in which V_H1-2*02 and precursor VRC01 IgL were knocked in (Tian et al., 2016) elicited mAbs with CDR H3 sequences substantially different from that of VRC01 UCA (Figure S3). Despite differences in murine and human D and J_H segment repertoires, on average, CDR H3 aa identity to VRC01 UCA was comparable among the two studies (21.5% for human B cells versus 20.5% for the murine B cells) (Figure S3). Thus, the Env outer domain proteins bound unequivocally well to VRC01 UCA. However, while eOD-GT8 engages VRC01-class naive B cells in humans, its reactivity is not exclusive to this class (Havenar-Daughton et al., 2018); hence, in the context of a stepwise immunization regimen, subsequent immunogens need be engineered to promote focusing

Clade	Subclade	CDR H3 sequence	mAb ID	CDR H3 length	Frequency in clade	
Clade 03+06		GG-YCSGGSCYNWDFQH	VRC01 UCA	16		
	subclade 03	R.-S.DYCGDF--PW.Y	VRC03, VRC03e, VRC03f, VRC03g	14	4/17	24%
	subclade 03	R.-S.DYCGGF--PW.Y	VRC03b, DH651.9	14	2/17	12%
	subclade 03	R.-P.DYCGDF--VW.Y	VRC03h, VRC03i	14	2/17	12%
	subclade 03	R.-P.DHCGDF--HW..	VRC03d	14	1/17	5.9%
	subclade 06	R.SS.PHCGDF--H.E.	VRC06, VRC06c, VRC06e, VRC06f, DH651.8	15	5/17	29%
	subclade 06	K.PS.PHCGDF--HW..	VRC06b	15	1/17	5.9%
	subclade 06	R.PS.PHCGDF--H.E.	VRC06d	15	1/17	5.9%
	subclade 06	R.SS.PHCGDF--H.EY	VRC06g	15	1/17	5.9%
Clade 08		GGYCSGG-------SCYNWDFQH	VRC01 UCA	16		
		.RS.C..RRHCNGAD.F......	VRC08	23	1/4	25%
		.KS.CN.RRYCNGAD.F....EY	VRC08c	23	1/4	25%
		.RS.CD.RRYCNGAD.FN...EN	VRC08d	23	1/4	25%
		.KS.CD.RRYCNGAD.F....ES	VRC08e	23	1/4	25%
Clade 01+07		GGYCSGGSCYNWDFQH	VRC01 UCA	16		
	subclade 07	.K..TARDY.....E.	VRC07b, VRC07c, VRC07d, VRC07f, NIH45-46, DH651.6, DH651.7	16	7/24	29%
	subclade 07	.K..TARDY.....EY	VRC07e	16	1/24	4.2%
	subclade 07	.KF.TESDY.....E.	DH651.5	16	1/24	4.2%
	subclade 01	.KN.----D.....E.	VRC01, VRC01d, VRC01f, VRC01g, VRC01h, VRC01j, VRC02	12	7/24	29%
	subclade 01	.KT.----D.....A.	VRC01c, DH651.2, DH651.4	12	3/24	13%
	subclade 01	.KD.----N.....E.	VRC01i, DH651.1	12	2/24	8.3%
	subclade 01	.KN.----DD....E.	DH651.3	12	1/24	4.2%
	subclade 01	.KN.----D.......	VRC01b	12	1/24	4.2%
	subclade 01	.KD.----N.....G.	VRC01e	12	1/24	4.2%

Clade	Subclade	CDR L1 sequence	mAb ID	CDR L1 length	Frequency in clade	
Clade 03+06		QSVSSSY	VRC01 UCA	7		
	subclade 03	.G--GNA	VRC03, VRC03b, VRC03g, DH651.9	5	4/17	24%
	subclade 03	.G--G.A	VRC03e, VRC03f	5	2/17	12%
	subclade 03	..--GNA	VRC03h	5	1/17	6%
	subclade 03	..--GNS	VRC03i	5	1/17	6%
	subclade 06	.G--GNS	VRC03d*, VRC06b, VRC06c, VRC06d, VRC06e, VRC06f, VRC06g,VRC06, DH651.8	5	9/17	53%
Clade 08		QSVSSSY	VRC01 UCA	7		
		.AI.K.H	VRC08e, VRC08c	7	2/4	50%
		.AI.KNH	VRC08d	7	1/4	25%
		.AITPRH	VRC08	7	1/4	25%
Clade 01+07		QSVSSSY	VRC01 UCA	7		
	subclade 07	..---GS	VRC07d, VRC07e, VRC07f, NIH45-46, DH651.6, DH651.7	4	6/24	25%
	subclade 01	.Y---GS	DH651.5*, VRC07c*, VRC07b*, VRC01, VRC01b,VRC01c, VRC01d, VRC01e, VRC01f, VRC01g, VRC01h, VRC01i, VRC01j, VRC02, DH651.1, DH651.2, DH651.3, DH651.4	4	18/24	75%

Figure 4. CDR H3 and CDR L1 Maturation Was Divergent across VRC01 Lineage Clades

Alignment of CDR H3 (left) and CDR L1 (right) aa sequences of the observed VRC01 lineage mAbs to the VRC01 UCA sequence (green). Sequences are grouped by clade and subclade membership. mAbs are listed by their respective sequences. mAbs with sequences that clustered outside their subclade membership are indicated with an asterisk.

of the B cell response and steer clonal evolution toward maturation pathways that will more likely result in the development of neutralization breadth.

Early Mutations Defined Maturation Pathways with Either Broad or Limited Neutralization

The reconstruction of the VRC01 genealogy from the UCA and the inference of the unobserved maturation intermediate antibodies (IA) confirmed that the VRC01 B cell lineage evolved into three divergent clades (clade 03+06, clade 08, and clade 01+07), as previously reported (Wu et al., 2015), and enabled to map the stages of clonal evolution at which clade-defining mutations occurred (Figure 3). The three clades diverged early during clonal maturation: clade 03+06 diverged from clades 01+07 and 08 at the first node (IA1), and clade 01+07 diverged from clade 08 at the subsequent node (IA2) (Figure 3). IA1 mutated 25 aa and IA2 further accumulated 9 aa mutations. Each clade further acquired distinct sets of indels in framework region (FR) H3, CDR H3, and CDR L1. Clade 03+06 inserted 21 nt at position 216 in FR H3 (IA5 node in Figure 3). Since FR H3 contacts the gp120 V1/V2 stem region, this insertion may render clade 03+06 mAbs more sensitive to variations in the Env V1/V2 loop (Zhou et al., 2010). The maturation of CDR H3 and CDR L1 are shown in Figure 4. CDR H3 underwent extensive modifications in all three clades and 80% of the mature mAbs introduced indels in this region. In clade 03+06, Asp100e and Trp100f were deleted. Trp100f is positioned 5 aa prior to the start of FR H4 and corresponds to Trp100b in the VRC01 bnAb aa sequence, which has been reported to be important for neutralization (West et al., 2012). Our reconstruction indicated that Trp100f was the result of the primary V-D-J recombination, not affinity maturation (Figure 4). Encouragingly, VRC01-class naive B cells with Trp100f can be sampled from the human naive B cell repertoire and enriched for using eOD-GT8 as a bait (Havenar-Daughton et al., 2018). Sub-clade 06 inserted an additional serine at position 96 in CDR H3, which further mutated to Pro96 within the sub-clade. mAbs in sub-clade 07 did not introduce indels in CDR H3. Conversely, sub-clade 01 mAbs deleted 12 nt resulting in the deletion of the ^{99}SGGS^{100b} aa motif and the C100cD mutation, which eliminated the inter-CDR H3 disulfide bond with Cys98 and formed the Env-contacting DYN motif (Zhou et al., 2010). Clade 08 mAbs introduced a 21-nt duplication which resulted in a 23-aa long CDR H3. Finally, clade-specific deletions developed in CDR L1, which interfaces with the Env N276 glycan: clade 03+06 mAbs deleted the aa ^{28}VS^{29} (IA5 node), and clade 01+07 mAbs similarly deleted ^{28}VSS^{30} at the IA3 node. CDR L1 aa sequences did not cluster within subclades as precisely as the CDR H3s. Notably, the CDR L1 of clade 08 mAbs did not acquire deletions (Figure 4).

The neutralizing activity of VRC01 UCA and the 45 mature mAbs was assessed on a panel of 12 HIV-1 strains representative of the global diversity (deCamp et al., 2014). VRC01 UCA did not neutralize any of the viruses tested (IC_{50} > 50 μg/mL). Clades 01+07 and 08 bnAbs displayed comparable breadth (geometric mean: 86% and 89%, respectively; median: 91.7% for both clades) whereas clade 03+06 mAbs were significantly narrower (geometric mean: 51%; median: 58.3%, $p < 0.01$), especially subclade 06 (geometric mean: 35%; median: 41.7%) (Figure 5A, Table S2). Only 26% of the strains were neutralized by clade 03+06 mAbs with IC_{80} < 50 μg/mL versus 79% and 73% for clade 01+07 and clade 08 mAbs, respectively (Table S3). Interclade differences in potency were less pronounced (geometric mean IC_{50} = 0.74 μg/mL, 0.68 μg/mL, and 2.4 μg/mL for clades 01+07, 08, and 03+06, respectively), with subclade 06 also comprising the least potent mAbs (geometric mean IC_{50} = 10.5 μg/mL) (Figure 5B).

Thus, we identified maturation pathways off-track (clade 03+06) and on-track (clades 01+07 and clade 08) toward broad

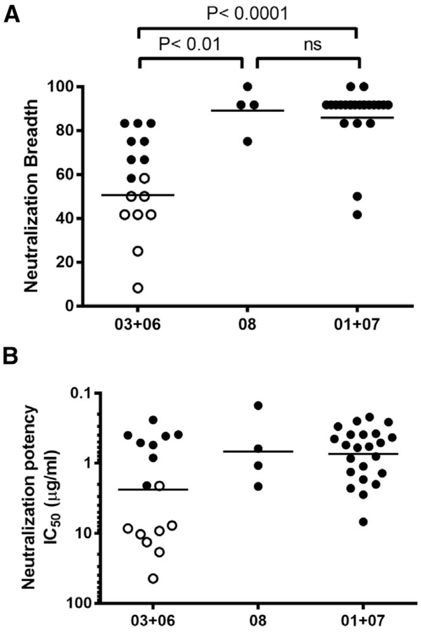

Figure 5. VRC01+07 and VRC08 Clade Antibodies Acquired Broad Neutralization whereas VRC03+06 Antibodies Displayed Limited Neutralization Breadth

(A) Neutralization was measured against the 12-virus global panel and expressed as percentage (y-axis). mAbs were grouped by clade membership: 16 in clade 03+06, 4 in clade 08, and 23 in clade 01+07. Subclade 06 mAbs are shown as clear dots. Lines indicate geometric mean. Results are representative of duplicate observations. Significance was evaluated using Kruskal-Wallis and Dunn's multiple comparisons tests at the alpha 0.05 level.

(B) Neutralization potency (IC_{50}) expressed in µg/mL (y-axis). Differences in potency across clades were not statistically significant.

See also Tables S2 and S3.

neutralization with clade 03+06 branching out at the earliest inferred intermediate (IA1). This finding suggested that a successful vaccine may need to focus early clonal maturation ("clade focusing") on track toward clades with the broadest neutralization. The VRC01 genealogy also informed the design of immunogens targeting early maturation IAs to select for mutations on-track toward the broadest clades (i.e., 01+07 and 08) and against mutations that define the 03+06 off-track VRC01 maturation pathway. These data also demonstrated that CDR L1 shortening alone was neither an absolute requirement (e.g., VRC08) nor sufficient (e.g., VRC01 lineage antibody DH651.8) to confer broad neutralization in the VRC01 lineage (Tables S2 and S3).

Heavy Chain, Not CDR L1, Is the Determinant to Accommodate the N276 Glycan in VRC08 Clade Antibodies

It is widely recognized that VRC01-class bnAbs evolved to accommodate the Env glycan at position N276 by either shortening or increasing the flexibility of their CDR L1 through SHM. The induction of robust VRC01-class antibody responses recognizing Envs with the N276 glycan remains, to date, elusive. Here we showed that, within the VRC01 lineage, clade 01+07 and clade 03+06 mAbs shortened their CDR L1 whereas clade 08 did not. To understand how VRC08 compensated for the suboptimal interaction between its CDR L1 and the HIV-1 Env N276 glycan, we superposed the VRC08 structure (PDB: 4XMP) (Wu et al., 2015) onto VRC01 in the JR-FL SOSIP complex (PDB: 5FYK) (Stewart-Jones et al., 2016) (Figures 6A and 6B). The VRC08 epitope size on the trimer was increased by ~50% (Figure 6A) compared to VRC01 (Figure 6B), largely due to the increased contact surface accounted for by the elongated CDR H3, as previously noted for the gp120 monomeric complex (Wu et al., 2015).

To determine the relative stability of the VRC08 CDR H3 conformation in the putative VRC08 SOSIP-bound state, we performed a 50 ns molecular dynamics simulation of VRC08 Fab bound to each protomer in a glycosylated JR-FL SOSIP trimer. The average backbone root-mean square deviation (RMSD) of the three individual CDR H3 loops were 1.4 ± 0.1 Å, 1.6 ± 0.2 Å, and 1.6 ± 0.2 Å, indicating that the loops remained stable in a state similar to that of the gp120-bound crystal structure configuration (PDB: 4XMP) (Wu et al., 2015) during the simulation (Figure S4 and Video S1). We investigated the interface between Env N276 glycan and CDR L1 for each protomer. Throughout the simulation, the average minimum and maximum distances between the N276 α-carbon and the CDR L1 α-carbon centers of geometry were ~10.0 Å and ~13.5 Å, respectively, with average distances of 12.3 ± 0.5 Å, 11.3 ± 0.7 Å, and 12.2 ± 0.5 Å for each protomer. Since CDR L1s were in close contact with the N276 residues, the conformation space available to the glycans was severely limited. However, the relatively narrow distance distribution between N276 and the CDR L1 in each protomer indicated that the CDR L1 loop conformation was stable throughout the simulation (Video S2). The N276 glycosylation site is predominantly occupied by minimally processed glycans (e.g., $Man_{5\text{-}9}GlcNAc_2$) in the context of trimeric SOSIP (Behrens et al., 2016); however, occupancy by complex glycans among naturally occurring virions may further reduce the solutions available to VRC01-class bnAbs with longer CDR L1, even though the ability of VRC08 to broadly and potently neutralize implies that unfavorable solutions on virions represent only a minority of cases.

The mature VRC08 CDR L1 was highly mutated (6 of 7 aa) and we sought to determine whether these mutations were responsible for accommodating the N276 glycan. We produced a chimeric antibody pairing VRC08 IgH with VRC01 UCA IgL ($VRC08_H/UCA_L$). $VRC08_H/UCA_L$ neutralized the fully glycosylated and the N276D mutant HIV-1 426c strain comparably (IC_{50} = 0.71 µg/mL and 0.11 µg/mL, respectively) (Figure 6C). Moreover, $VRC08_H/UCA_L$ retained 50% of neutralization breadth of the mature VRC08 bnAb (Figure 6C). Thus, the CDR L1 mutations in the mature VRC08 bnAb, while beneficial to breadth, were not essential to accommodate the N276 glycan. These data demonstrated that VRC01-class bnAb precursors can circumvent the barrier posed by the N276 glycan to initiate broad neutralization by using IgH chains with a long CDR H3 without the need of mutating or shortening the germline-encoded CDR L1. We hypothesize that the CDR H3 elongation in VRC08 leads to an increase in enthalpy due to expanded epitope contacts. This enthalpy increase then could compensate for the entropy penalty associated with trapping the N276 glycan in a limited number of accommodating conformations without requiring a deletion in CDR L1. Thus, the

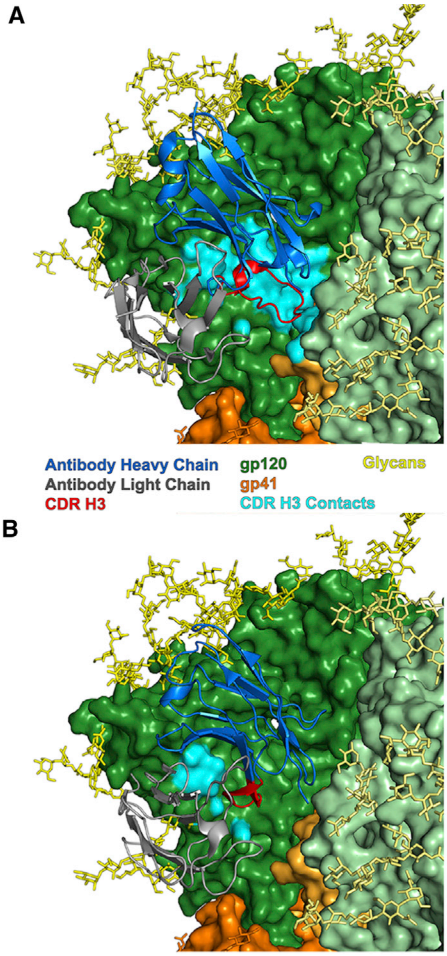

C

IC$_{50}$ (μg/ml)

	VRC01 UCA	VRC08$_H$/UCA$_L$	VRC08
426c WT	>50	0.71	0.10
426c N276D	>50	0.11	<0.023
X2278	>50	0.21	<0.023
CNE55	>50	0.44	0.14
TRO.11	>50	0.65	0.12
CNE8	>50	1.7	0.19
25710	>50	6.8	0.07
246-F3	>50	13	0.35
Ce0217	>50	>50	0.04
CH119	>50	>50	0.06
BJOX002	>50	>50	0.20
Ce1176	>50	>50	0.41
398-F1	>50	>50	0.42
X1632	>50	>50	14
MLV-SVA	>50	>50	>50

Figure 6. Heavy Chain, Not CDR L1, Is the Determinant to Accommodate the N276 Glycan in VRC08 bnAb and Initiate Breadth

(A) Superposition of the VRC08 structure onto the JRFL SOSIP structure in complex with VRC01. Coloring scheme is as follows: heavy chain (blue), light chain (gray), CDR H3 (red), CDR H3 contact surface (cyan), gp120s (green shades), and gp41s (orange shades). Glycans are shown in stick representation. The N301 glycan was removed from the view for clarity.

(B) Structure of VRC01 in complex with JR-FL SOSIP used to superpose VRC08, with same color scheme of (A).

(C) Heat map analysis of neutralization data of VRC01 UCA, VRC08$_H$/UCA$_L$ chimera, and mature VRC08 bnAb (columns) against the 426c wild-type HIV-1 strain and its N276D mutant (in which the potential N-linked glycosylation site at position 276 was abrogated) and the 12-virus global panel. MLV-SVA is shown as negative control. Neutralization potency IC$_{50}$ is expressed in μg/mL and coloring ranges from white (>50 μg/mL) to dark red (<0.023 μg/mL). Results are representative of at least duplicate experiments.

See also Figure S4, Videos S1 and S2.

preferential engagement of VRC01-class naive B cells with long CDR H3s may be an alternative strategy to overcome the N276 glycan barrier.

VRC01 Lineage mAbs Do Not Need to Be Auto- or Polyreactive to Broadly Neutralize

VRC01 lineage bnAbs can be either polyreactive or autorective with Ubiquitin Protein Ligase E3A (UBE3A) (Liu et al., 2015) and studies in GL VRC01-class 3BNC60 knock-in mice suggested that VRC01 class bnAb precursors may be regulated by tolerance mechanisms (McGuire et al., 2016). We measured auto- and polyreactivity of the VRC01 UCA, GL VRC01-class mAbs, and the 45 naturally paired IgH+IgL mature VRC01 lineage mAbs. VRC01 UCA was not auto- or polyreactive (Figure S5). In comparison, 4 of 9 GL VRC01-class mAbs were self-reactive: VRC01 V.Rev was at the threshold for polyreactivity; VRC18b V.Rev displayed a cytoskeleton pattern in HEp2 cell IFA staining; and VRC20 V.Rev and VRC23 V.Rev were polyreactive, displayed cytoplasmic IFA staining, and bound, respectively, to centromere B and all nine autoantigens (Figure S5). Notably, VRC01 V.Rev, VRC18b V.Rev, and VRC20 V.Rev were among the mAbs with the most favorable binding kinetics for the immunogens designed on GL mAbs. Of the 45 mature VRC01 lineage mAbs, 36 (80%) were self-reactive (Figure 7A). However, non-auto or polyreactive mAbs neutralized as broadly and potently as the auto-/polyreactive ones (Figures 7B and 7C). Non-auto or polyreactive mAbs were distributed across the three VRC01 lineage clades and included the VRC08 bnAb, which was as broad and potent as the VRC01 bnAb. There was no correlation between neutralization breadth and potency versus polyreactivity at the alpha 0.05 level (Spearman correlation: −0.2896 and 0.2003, respectively; $p > 0.05$) (Figures 7D and 7E).

UBE3A-reactive mAbs clustered exclusively in clade 01+07 and represented the majority of mAbs in this clade (19/24; 79.2%). We noted that the germline-encoded ^{33}YM^{34} motif in CDR H1 mutated in all 45 mature mAbs and that subclade 01 predominantly mutated to ^{33}TL^{34} (Figure S6A). mAbs with ^{33}TL^{34} were significantly more reactive with UBE3A ($p < 0.001$; Figure S6B). Reversion of ^{33}TL^{34} to the germline-encoded ^{33}YM^{34} in subclade 01 VRC01, VRC02, DH651.1, DH651.2, and DH651.4 bnAbs abrogated UBE3A reactivity (Figure S6C). mAbs with reversion mutations that restored the germline-encoded YM motif retained full breadth with a modest (1.4- to 2.7-fold) reduction in potency (Figure S6D). We introduced the T33Y and L34M reversion mutations separately in VRC01: their neutralization profiles were comparable to that of the double ^{33}YM^{34} mutation (Figure S6D) and, while the single L34M reversion mutation did not affect VRC01 binding to UBE3A, T33Y was sufficient to abrogate VRC01 bnAb UBE3A reactivity (Figure S6E). The Y33T mutation was estimated to be improbable (Y33T probability < 1% versus 28.5% of M34L; Figure S6F) (Wiehe et al., 2018), and since Tyr33 interacts with HIV-1 envelope protein gp120 (Zhou et al., 2010), the Y33T mutation was

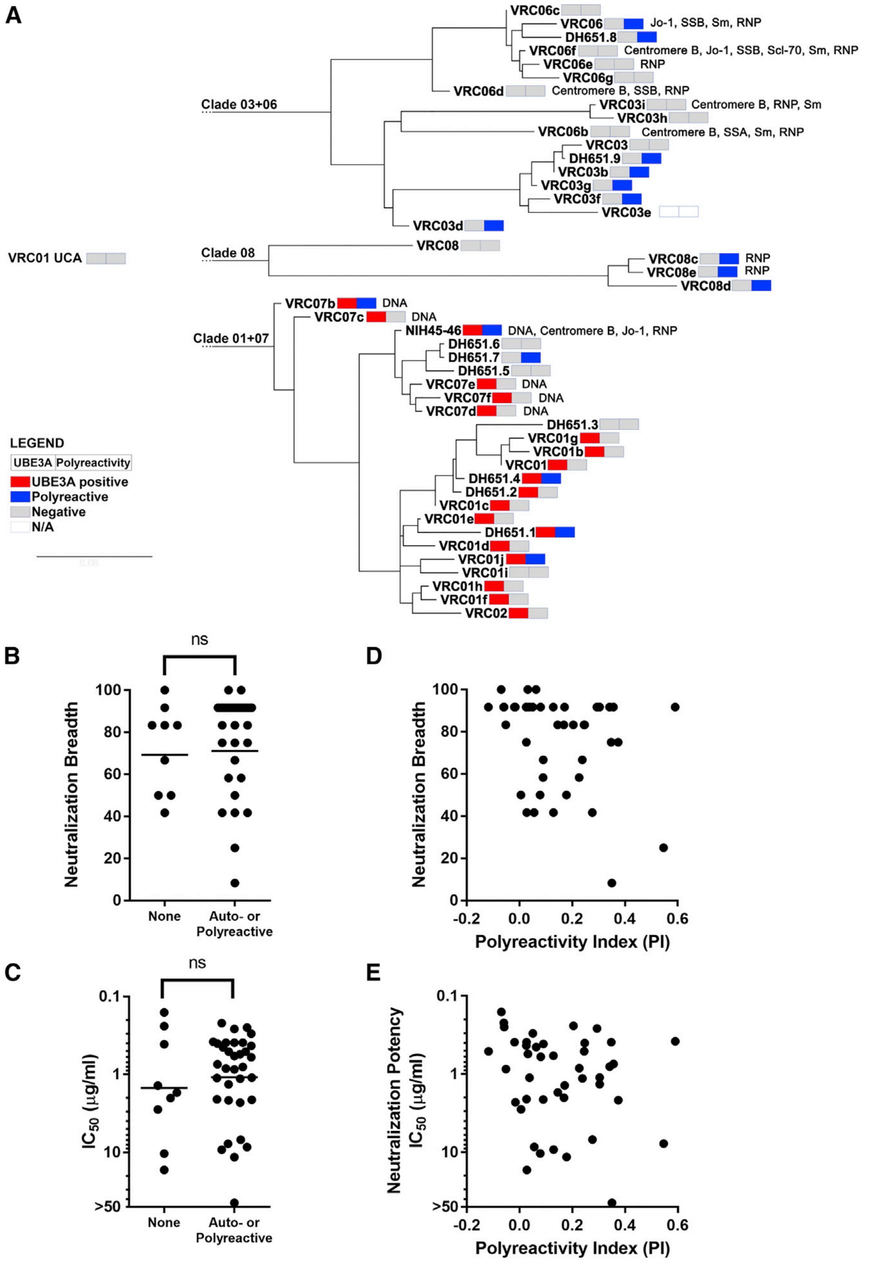

Figure 7. Lack of Relationship between Auto- and Polyreactivity and Neutralization Breadth in Mature VRC01 Lineage bnAbs

(A) UBE3A reactivity (red), polyreactivity (blue), and anti-nuclear antigens (ANA) autoreactivity are shown for each mAb.

(B and C) Neutralization breadth (B) and potency (C) of non-auto-/polyreactive (n = 9) and auto-polyreactive (n = 34) antibodies as defined in (A). Significance was evaluated with the Mann-Whitney U test at the alpha 0.05 level.

(D and E) Lack of correlation between neutralization breadth (D) or potency (E) and polyreactivity.

See also Figures S5 and S6.

likely the result of strong antigen-driven positive selection in subclade 01 mAbs.

Thus, these data demonstrated that VRC01 UCA is not auto- or polyreactive and that acquisition of auto-/polyreactivity by VRC01 lineage mAbs was not a requirement for development of neutralization breadth.

DISCUSSION

The lack of accurate reconstructions of the paired IgH and IgL genealogy of VRC01-class bnAb lineages from their UCA has posed substantial hurdles in devising strategies to identify and overcome roadblocks that impede the elicitation of VRC01-class bnAbs through vaccination. Here we have described the inference of the UCA of the VRC01 lineage and reconstructed the stages of the VRC01 lineage maturation.

We demonstrated that existing Env immunogens designed on GL VRC01-class bnAbs bound to VRC01 UCA with affinity sufficient to activate naive B cells; that early clade focusing led to maturation pathways on- and off-track to broad neutralization; and that solutions to breadth exist that did not require shortening or increased flexibility of the CDR L1 to accommodate the Env N276 glycan.

Reproducing consistently the complex evolution of the VRC01 lineage described here (including the acquisition of extensive SHM and indels) in its entirety through vaccination is likely to be challenging. In particular, if and how the frequency of indels can be manipulated through vaccination is unknown and future studies should explore strategies to address this issue. However, our observations indicated possible alternative maturation end points that may suffice to induce or initiate the development of VRC01-class bnAbs. Inducing the maturation of VRC01-class bnAbs from unmutated precursors that can neutralize HIV-1 strains with the N276 glycan site remains a critical barrier for vaccine development. While clade 01+07 circumvented this barrier by shortening CDR L1, clade 08 mAbs retained normal-length CDR L1s and mutated their IgH with a large insertion in CDR H3. This observation raised the hypothesis that, among VRC01-class bnAb precursors with longer CDR H3 in the naive B cell repertoire, at least some will naturally bypass the N276 glycan barrier and may more readily progress to breadth. Interestingly, Havenar-Daughton et al. (2018) have recently demonstrated a modest inverse correlation between CDR H3 length and affinity of naive VRC01-class B cells to eOD-GT8, which may result in a suboptimal engagement of this subpopulation of naive B cells in presence of naive VRC01-class B cells with shorter CDR H3s. Since long CDR H3s are typically the result of the original V_HDJ_H recombination and antibodies using

the V_H1 gene family are naturally skewed toward primary rearrangements with long CDR H3 (Briney et al., 2012), future studies should investigate whether this subpopulation of VRC01-class precursors can be preferentially targeted through immunogen design.

Three distinct maturation pathways in the VRC01 lineage diverged early during clonal development, leading to either relatively limited (clade 03+06) or broad (clades 01+07 and 08) neutralization. That most of clade 03+06 mAbs acquired SHM leading to limited neutralization breadth emphasizes that breadth is not an end point of lineage maturation in natural infection and we speculate that, due to the polyreactive nature of the VRC01 lineage, the process of antibody redemption may have also contributed to shape its evolution (Reed et al., 2016; Sabouri et al., 2014). Previous immunization strategies may have failed to effectively focus maturation to the desired potently neutralizing clades at the earliest stages of clonal maturation. This study identified limited sets of early, clade-defining mutations that provide a roadmap to design immunogens that will guide the selection of early intermediates on-track toward neutralization breadth (Wiehe et al., 2018). Possible end points of VRC01 maturation resulting in the acquisition of various levels of neutralization breadth with limited SHM have been described (Georgiev et al., 2014; Jardine et al., 2016b). Of them, a mAb referred to as minVRC01 reaches 84% breadth with 21 amino acid mutations, far fewer than the 69 aa mutations in VRC01 bnAb (excluding mutations in CDR H3 and the CDR L1 deletion) (Jardine et al., 2016b). However, this was achieved starting from a mature CDR H3 and a shortened CDR L1, which is a rate-limiting maturation event. In addition, 15 of the 21 aa mutations in minVRC01 are improbable (unpublished data), suggesting that the pathway to minVRC01 will be challenging to elicit. Together with new computational tools that assign the probability of specific mutations throughout clonal maturation (Wiehe et al., 2018), the VRC01 genealogy described here enables a more precise definition of the most efficient routes to neutralization breadth in the VRC01 lineage. Antibody mutations occur with varying frequencies prior to selection due to the stochastic nature of the SHM process (Betz et al., 1993; Pham et al., 2003; Victora and Nussenzweig, 2012) and, while the detailed genealogy provides a map of the mutational routes, information about the probabilities of mutations prior to immunogenic selection along those routes helps in defining how difficult (or easily) these mutations can be selected. Beneficial mutations that are probable should be readily available in germinal centers due to normal immune activation. Conversely, beneficial improbable mutations require strong selective pressure to arise during SHM and can act as bottlenecks in the development of bnAbs (Bonsignori et al., 2017). Mutations detrimental to neutralization can be barriers to bnAb lineage development as well and, if detrimental mutations are also highly probable, they can act as diverting forces for B cell maturation, directing a component of a clone off-track. The implications for vaccine development are that critical improbable mutations represent the highest value targets for selection in vaccine design strategies and, concurrently, detrimental probable mutations may need to be selected against.

In conclusion, the reconstruction of the VRC01 lineage UCA, the higher level of detail of the routes to neutralization breadth through the newly reconstructed VRC01 genealogy, and a better understanding of the probabilities of individual mutations in the lineage (Wiehe et al., 2018) provide a valuable dataset to design immunogens that will efficiently select for B cell maturation pathways leading to broad neutralization.

STAR★METHODS

Detailed methods are provided in the online version of this paper and include the following:

- KEY RESOURCES TABLE
- CONTACT FOR REAGENT AND RESOURCE SHARING
- EXPERIMENTAL MODEL AND SUBJECT DETAILS
  - Human specimens
  - Cell lines
- METHOD DETAILS
  - Isolation of VRC01 lineage antibodies
  - Antibody production
  - Inference of the VRC01 clonal history including UCAs and unobserved intermediates
  - Surface Plasmon Resonance Affinity and Kinetics Measurements
  - Biolayer Interferometry (BLI)
  - Calcium Flux Measurement
  - Assessment of virus neutralization
  - Immunogens
  - Molecular Dynamics Simulation
  - HEp-2 Cell Staining
  - Protein Array
  - Indirect-binding ELISA
  - Antibody site-directed mutagenesis
  - Logo plots
- QUANTIFICATION AND STATISTICAL ANALYSIS
- DATA AND SOFTWARE AVAILABILITY

SUPPLEMENTAL INFORMATION

Supplemental information includes six figures, three tables, and two videos and can be found with the article online at https://doi.org/10.1016/j.immuni.2018.10.015.

ACKNOWLEDGMENTS

We thank Kelly Soderberg and Ashley Trama for project management; Amanda Eaton, Morgan Gladden, Anthony Monroe, Kara Anasti, Andrew Foulger, Giovanna Hernandez, Maggie Barr, and Robert Parks for technical support; and Wes Rountree for statistical analysis. This study was supported by the Duke Center for HIV/AIDS Vaccine Immunology-Immunogen Discovery grant UM1AI100645 from the National Institutes of Health (NIH), National Institutes of Allergy and Infectious Diseases (NIAID) to B.F.H. Additional support to this study was provided by the NIH, NIAID grant R01AI081625 to L.S.; by the Ruth L. Kirschstein National Research Service Award (NRSA) grant T32AI007392 to E.S.; the Aids Fonds Netherlands grant 20160192 and the Collaboration for AIDS Vaccine Discovery (CAVD) grant OPP1132237 from the Bill & Melinda Gates Foundation to R.W.S.; and the intramural research programs of the NIAID Vaccine Research Center and the NIAID Division of Intramural Research to P.D.K., J.R.M and M. Connors.

AUTHOR CONTRIBUTIONS

Conceptualization, M.B. and B.F.H.; Methodology: M.B., K.W., D.E., and K.-K.H.; Software: K.W. and T.B.K.; Formal analysis: M.B., K.W., D.E., S.M.A., R.H., and T.B.K.; Investigation: E.S., K.W., D.E., K.-K.H., M. Cooper,

S.-M.X., R.Z., R.H., X.N., A.T.M., and T.B.K.; Resources: D.C.M., X.C., M.A.M., M.G.J., P.D.K., M. Connors, J.R.M., A.T.M., L.S., M.M.-R., R.W.S., and K.O.S.; Data curation: M.B.; Writing – Original Draft: M.B.; Writing – Review and Editing: all authors; Visualization: M.B., E.S., K.W., D.E., R.H., and A.T.M.; Supervision: M.B., G.K., P.D.K., M. Connors, J.R.M., L.S., K.O.S., and B.F.H.; Project Administration: M.B., K.O.S., and B.F.H.

DECLARATION OF INTEREST

The authors declare no competing interests.

Received: June 13, 2018
Revised: September 7, 2018
Accepted: October 19, 2018
Published: December 11, 2018

REFERENCES

Balazs, A.B., Ouyang, Y., Hong, C.M., Chen, J., Nguyen, S.M., Rao, D.S., An, D.S., and Baltimore, D. (2014). Vectored immunoprophylaxis protects humanized mice from mucosal HIV transmission. Nat. Med. *20*, 296–300.

Barouch, D.H., Whitney, J.B., Moldt, B., Klein, F., Oliveira, T.Y., Liu, J., Stephenson, K.E., Chang, H.W., Shekhar, K., Gupta, S., et al. (2013). Therapeutic efficacy of potent neutralizing HIV-1-specific monoclonal antibodies in SHIV-infected rhesus monkeys. Nature *503*, 224–228.

Behrens, A.J., Vasiljevic, S., Pritchard, L.K., Harvey, D.J., Andev, R.S., Krumm, S.A., Struwe, W.B., Cupo, A., Kumar, A., Zitzmann, N., et al. (2016). Composition and antigenic effects of individual glycan sites of a trimeric HIV-1 envelope glycoprotein. Cell Rep. *14*, 2695–2706.

Benjamin, D., Magrath, I.T., Maguire, R., Janus, C., Todd, H.D., and Parsons, R.G. (1982). Immunoglobulin secretion by cell lines derived from African and American undifferentiated lymphomas of Burkitt's and non-Burkitt's type. J. Immunol. *129*, 1336–1342.

Betz, A.G., Rada, C., Pannell, R., Milstein, C., and Neuberger, M.S. (1993). Passenger transgenes reveal intrinsic specificity of the antibody hypermutation mechanism: clustering, polarity, and specific hot spots. Proc. Natl. Acad. Sci. USA *90*, 2385–2388.

Bonsignori, M., Hwang, K.K., Chen, X., Tsao, C.Y., Morris, L., Gray, E., Marshall, D.J., Crump, J.A., Kapiga, S.H., Sam, N.E., et al. (2011). Analysis of a clonal lineage of HIV-1 envelope V2/V3 conformational epitope-specific broadly neutralizing antibodies and their inferred unmutated common ancestors. J. Virol. *85*, 9998–10009.

Bonsignori, M., Wiehe, K., Grimm, S.K., Lynch, R., Yang, G., Kozink, D.M., Perrin, F., Cooper, A.J., Hwang, K.K., Chen, X., et al. (2014). An autoreactive antibody from an SLE/HIV-1 individual broadly neutralizes HIV-1. J. Clin. Invest. *124*, 1835–1843.

Bonsignori, M., Zhou, T., Sheng, Z., Chen, L., Gao, F., Joyce, M.G., Ozorowski, G., Chuang, G.Y., Schramm, C.A., Wiehe, K., et al.; NISC Comparative Sequencing Program (2016). Maturation pathway from germline to broad HIV-1 neutralizer of a CD4-mimic antibody. Cell *165*, 449–463.

Bonsignori, M., Kreider, E.F., Fera, D., Meyerhoff, R.R., Bradley, T., Wiehe, K., Alam, S.M., Aussedat, B., Walkowicz, W.E., Hwang, K.K., et al. (2017). Staged induction of HIV-1 glycan-dependent broadly neutralizing antibodies. Sci. Transl. Med. *9*, 9.

Briney, B.S., Willis, J.R., and Crowe, J.E., Jr. (2012). Human peripheral blood antibodies with long HCDR3s are established primarily at original recombination using a limited subset of germline genes. PLoS ONE *7*, e36750.

Briney, B., Sok, D., Jardine, J.G., Kulp, D.W., Skog, P., Menis, S., Jacak, R., Kalyuzhniy, O., de Val, N., Sesterhenn, F., et al. (2016). Tailored immunogens direct affinity maturation toward HIV neutralizing antibodies. Cell *166*, 1459–1470.e11.

Caskey, M., Klein, F., Lorenzi, J.C., Seaman, M.S., West, A.P., Jr., Buckley, N., Kremer, G., Nogueira, L., Braunschweig, M., Scheid, J.F., et al. (2015). Viraemia suppressed in HIV-1-infected humans by broadly neutralizing antibody 3BNC117. Nature *522*, 487–491.

Davenport, T.M., Gorman, J., Joyce, M.G., Zhou, T., Soto, C., Guttman, M., Moquin, S., Yang, Y., Zhang, B., Doria-Rose, N.A., et al. (2016). Somatic hypermutation-induced changes in the structure and dynamics of HIV-1 broadly neutralizing antibodies. Structure *24*, 1346–1357.

deCamp, A., Hraber, P., Bailer, R.T., Seaman, M.S., Ochsenbauer, C., Kappes, J., Gottardo, R., Edlefsen, P., Self, S., Tang, H., et al. (2014). Global panel of HIV-1 Env reference strains for standardized assessments of vaccine-elicited neutralizing antibodies. J. Virol. *88*, 2489–2507.

Diskin, R., Scheid, J.F., Marcovecchio, P.M., West, A.P., Jr., Klein, F., Gao, H., Gnanapragasam, P.N., Abadir, A., Seaman, M.S., Nussenzweig, M.C., and Bjorkman, P.J. (2011). Increasing the potency and breadth of an HIV antibody by using structure-based rational design. Science *334*, 1289–1293.

Dosenovic, P., von Boehmer, L., Escolano, A., Jardine, J., Freund, N.T., Gitlin, A.D., McGuire, A.T., Kulp, D.W., Oliveira, T., Scharf, L., et al. (2015). Immunization for HIV-1 broadly neutralizing antibodies in human Ig knockin mice. Cell *161*, 1505–1515.

Essmann, U., Perera, L., Berkowitz, M.L., Darden, T., Lee, H., and Pedersen, L.G. (1995). A smooth particle mesh Ewald method. J. Chem. Phys. *103*, 8577–8593.

Felsenstein, J. (2005). PHYLIP (Phylogeny Inference Package), 3.6a3 edn (Seattle, WA: distributed by the author: Department of Genome Sciences, University of Washington).

Gao, F., Bonsignori, M., Liao, H.X., Kumar, A., Xia, S.M., Lu, X., Cai, F., Hwang, K.K., Song, H., Zhou, T., et al. (2014). Cooperation of B cell lineages in induction of HIV-1-broadly neutralizing antibodies. Cell *158*, 481–491.

Georgiev, I.S., Doria-Rose, N.A., Zhou, T., Kwon, Y.D., Staupe, R.P., Moquin, S., Chuang, G.Y., Louder, M.K., Schmidt, S.D., Altae-Tran, H.R., et al. (2013). Delineating antibody recognition in polyclonal sera from patterns of HIV-1 isolate neutralization. Science *340*, 751–756.

Georgiev, I.S., Rudicell, R.S., Saunders, K.O., Shi, W., Kirys, T., McKee, K., O'Dell, S., Chuang, G.Y., Yang, Z.Y., Ofek, G., et al. (2014). Antibodies VRC01 and 10E8 neutralize HIV-1 with high breadth and potency even with Ig-framework regions substantially reverted to germline. J. Immunol. *192*, 1100–1106.

Gray, E.S., Moody, M.A., Wibmer, C.K., Chen, X., Marshall, D., Amos, J., Moore, P.L., Foulger, A., Yu, J.S., Lambson, B., et al. (2011). Isolation of a monoclonal antibody that targets the alpha-2 helix of gp120 and represents the initial autologous neutralizing-antibody response in an HIV-1 subtype C-infected individual. J. Virol. *85*, 7719–7729.

Guindon, S., Dufayard, J.-F., Lefort, V., Anisimova, M., Hordijk, W., and Gascuel, O. (2010). New algorithms and methods to estimate maximum-likelihood phylogenies: assessing the performance of PhyML 3.0. Syst. Biol. *59*, 307–321.

Hall, T.A. (1999). BioEdit: a user-friendly biological sequence alignment editor and analysis program for Windows 95/98/NT. Nucl. Acids Symp. Ser *41*, 95–98.

Havenar-Daughton, C., Sarkar, A., Kulp, D.W., Toy, L., Hu, X., Deresa, I., Kalyuzhniy, O., Kaushik, K., Upadhyay, A.A., Menis, S., et al. (2018). The human naive B cell repertoire contains distinct subclasses for a germline-targeting HIV-1 vaccine immunogen. Sci. Transl. Med. *10*, 10.

Haynes, B.F., Fleming, J., St Clair, E.W., Katinger, H., Stiegler, G., Kunert, R., Robinson, J., Scearce, R.M., Plonk, K., Staats, H.F., et al. (2005). Cardiolipin polyspecific autoreactivity in two broadly neutralizing HIV-1 antibodies. Science *308*, 1906–1908.

Haynes, B.F., Kelsoe, G., Harrison, S.C., and Kepler, T.B. (2012). B-cell-lineage immunogen design in vaccine development with HIV-1 as a case study. Nat. Biotechnol. *30*, 423–433.

Humphrey, W., Dalke, A., and Schulten, K. (1996). VMD: visual molecular dynamics. J. Mol. Graph. *14*, 33–38, 27–28.

Jardine, J., Julien, J.P., Menis, S., Ota, T., Kalyuzhniy, O., McGuire, A., Sok, D., Huang, P.S., MacPherson, S., Jones, M., et al. (2013). Rational HIV immunogen design to target specific germline B cell receptors. Science *340*, 711–716.

Jardine, J.G., Ota, T., Sok, D., Pauthner, M., Kulp, D.W., Kalyuzhniy, O., Skog, P.D., Thinnes, T.C., Bhullar, D., Briney, B., et al. (2015). HIV-1 VACCINES. Priming a broadly neutralizing antibody response to HIV-1 using a germline-targeting immunogen. Science *349*, 156–161.

Jardine, J.G., Kulp, D.W., Havenar-Daughton, C., Sarkar, A., Briney, B., Sok, D., Sesterhenn, F., Ereño-Orbea, J., Kalyuzhniy, O., Deresa, I., et al. (2016a). HIV-1 broadly neutralizing antibody precursor B cells revealed by germline-targeting immunogen. Science *351*, 1458–1463.

Jardine, J.G., Sok, D., Julien, J.P., Briney, B., Sarkar, A., Liang, C.H., Scherer, E.A., Henry Dunand, C.J., Adachi, Y., Diwanji, D., et al. (2016b). Minimally mutated HIV-1 broadly neutralizing antibodies to guide reductionist vaccine design. PLoS Pathog. *12*, e1005815.

Jorgensen, W.L., Chandrasekhar, J., Madura, J.D., Impey, R.W., and Klein, M.L. (1983). Comparison of simple potential functions for simulating liquid water. J. Chem. Phys. *79*, 926–935.

Kabat, E.A., Wu, T.T., Perry, H., Gottesman, K., and Foeller, C. (1991). Sequences of Proteins of Immunological Interest. NIH Publication No. 91-3242, Fifth Edition.

Kepler, T.B. (2013). Reconstructing a B-cell clonal lineage. I. Statistical inference of unobserved ancestors. F1000Res. *2*, 103.

Kepler, T.B., Munshaw, S., Wiehe, K., Zhang, R., Yu, J.S., Woods, C.W., Denny, T.N., Tomaras, G.D., Alam, S.M., Moody, M.A., et al. (2014). Reconstructing a B-cell clonal lineage. II. Mutation, selection, and affinity maturation. Front. Immunol. *5*, 170.

Klein, F., Diskin, R., Scheid, J.F., Gaebler, C., Mouquet, H., Georgiev, I.S., Pancera, M., Zhou, T., Incesu, R.B., Fu, B.Z., et al. (2013). Somatic mutations of the immunoglobulin framework are generally required for broad and potent HIV-1 neutralization. Cell *153*, 126–138.

Li, Y., O'Dell, S., Wilson, R., Wu, X., Schmidt, S.D., Hogerkorp, C.M., Louder, M.K., Longo, N.S., Poulsen, C., Guenaga, J., et al. (2012). HIV-1 neutralizing antibodies display dual recognition of the primary and coreceptor binding sites and preferential binding to fully cleaved envelope glycoproteins. J. Virol. *86*, 11231–11241.

Liao, H.X., Levesque, M.C., Nagel, A., Dixon, A., Zhang, R., Walter, E., Parks, R., Whitesides, J., Marshall, D.J., Hwang, K.K., et al. (2009). High-throughput isolation of immunoglobulin genes from single human B cells and expression as monoclonal antibodies. J. Virol. Methods *158*, 171–179.

Liao, H.X., Lynch, R., Zhou, T., Gao, F., Alam, S.M., Boyd, S.D., Fire, A.Z., Roskin, K.M., Schramm, C.A., Zhang, Z., et al.; NISC Comparative Sequencing Program (2013). Co-evolution of a broadly neutralizing HIV-1 antibody and founder virus. Nature *496*, 469–476.

Liu, M., Yang, G., Wiehe, K., Nicely, N.I., Vandergrift, N.A., Rountree, W., Bonsignori, M., Alam, S.M., Gao, J., Haynes, B.F., and Kelsoe, G. (2015). Polyreactivity and autoreactivity among HIV-1 antibodies. J. Virol. *89*, 784–798.

Luo, X.M., Maarschalk, E., O'Connell, R.M., Wang, P., Yang, L., and Baltimore, D. (2009). Engineering human hematopoietic stem/progenitor cells to produce a broadly neutralizing anti-HIV antibody after in vitro maturation to human B lymphocytes. Blood *113*, 1422–1431.

Lynch, R.M., Boritz, E., Coates, E.E., DeZure, A., Madden, P., Costner, P., Enama, M.E., Plummer, S., Holman, L., Hendel, C.S., et al.; VRC 601 Study Team (2015). Virologic effects of broadly neutralizing antibody VRC01 administration during chronic HIV-1 infection. Sci. Transl. Med. *7*, 319ra206.

Martyna, G.J., Tobias, D.J., and Klein, M.L. (1994). Constant pressure molecular dynamics algorithms. J. Chem. Phys. *101*, 4177–4189.

McGuire, A.T., Hoot, S., Dreyer, A.M., Lippy, A., Stuart, A., Cohen, K.W., Jardine, J., Menis, S., Scheid, J.F., West, A.P., et al. (2013). Engineering HIV envelope protein to activate germline B cell receptors of broadly neutralizing anti-CD4 binding site antibodies. J. Exp. Med. *210*, 655–663.

McGuire, A.T., Gray, M.D., Dosenovic, P., Gitlin, A.D., Freund, N.T., Petersen, J., Correnti, C., Johnsen, W., Kegel, R., Stuart, A.B., et al. (2016). Specifically modified Env immunogens activate B-cell precursors of broadly neutralizing HIV-1 antibodies in transgenic mice. Nat. Commun. *7*, 10618.

Medina-Ramírez, M., Garces, F., Escolano, A., Skog, P., de Taeye, S.W., Del Moral-Sanchez, I., McGuire, A.T., Yasmeen, A., Behrens, A.J., Ozorowski, G., et al. (2017). Design and crystal structure of a native-like HIV-1 envelope trimer that engages multiple broadly neutralizing antibody precursors in vivo. J. Exp. Med. *214*, 2573–2590.

Montefiori, D.C. (2005). Evaluating neutralizing antibodies against HIV, SIV, and SHIV in luciferase reporter gene assays. Curr. Protoco. Immunol. Unit 12 11.

Ota, T., Doyle-Cooper, C., Cooper, A.B., Huber, M., Falkowska, E., Doores, K.J., Hangartner, L., Le, K., Sok, D., Jardine, J., et al. (2012). Anti-HIV B Cell lines as candidate vaccine biosensors. J. Immunol. *189*, 4816–4824.

Pegu, A., Yang, Z.Y., Boyington, J.C., Wu, L., Ko, S.Y., Schmidt, S.D., McKee, K., Kong, W.P., Shi, W., Chen, X., et al. (2014). Neutralizing antibodies to HIV-1 envelope protect more effectively in vivo than those to the CD4 receptor. Sci. Transl. Med. *6*, 243ra88.

Pham, P., Bransteitter, R., Petruska, J., and Goodman, M.F. (2003). Processive AID-catalysed cytosine deamination on single-stranded DNA simulates somatic hypermutation. Nature *424*, 103–107.

Phillips, J.C., Braun, R., Wang, W., Gumbart, J., Tajkhorshid, E., Villa, E., Chipot, C., Skeel, R.D., Kalé, L., and Schulten, K. (2005). Scalable molecular dynamics with NAMD. J. Comput. Chem. *26*, 1781–1802.

Pietzsch, J., Gruell, H., Bournazos, S., Donovan, B.M., Klein, F., Diskin, R., Seaman, M.S., Bjorkman, P.J., Ravetch, J.V., Ploss, A., and Nussenzweig, M.C. (2012). A mouse model for HIV-1 entry. Proc. Natl. Acad. Sci. USA *109*, 15859–15864.

Reed, J.H., Jackson, J., Christ, D., and Goodnow, C.C. (2016). Clonal redemption of autoantibodies by somatic hypermutation away from self-reactivity during human immunization. J. Exp. Med. *213*, 1255–1265.

Rudicell, R.S., Kwon, Y.D., Ko, S.Y., Pegu, A., Louder, M.K., Georgiev, I.S., Wu, X., Zhu, J., Boyington, J.C., Chen, X., et al.; NISC Comparative Sequencing Program (2014). Enhanced potency of a broadly neutralizing HIV-1 antibody in vitro improves protection against lentiviral infection in vivo. J. Virol. *88*, 12669–12682.

Ryckaert, J.-P., Ciccotti, G., and Berendsen, H.J.C. (1977). Numerical integration of the cartesian equations of motion of a system with constraints: molecular dynamics of n-alkanes. J. Comput. Phys. *23*, 327–341.

Sabouri, Z., Schofield, P., Horikawa, K., Spierings, E., Kipling, D., Randall, K.L., Langley, D., Roome, B., Vazquez-Lombardi, R., Rouet, R., et al. (2014). Redemption of autoantibodies on anergic B cells by variable-region glycosylation and mutation away from self-reactivity. Proc. Natl. Acad. Sci. USA *111*, E2567–E2575.

Sali, A., and Blundell, T.L. (1993). Comparative protein modelling by satisfaction of spatial restraints. J. Mol. Biol. *234*, 779–815.

Scharf, L., West, A.P., Jr., Gao, H., Lee, T., Scheid, J.F., Nussenzweig, M.C., Bjorkman, P.J., and Diskin, R. (2013). Structural basis for HIV-1 gp120 recognition by a germ-line version of a broadly neutralizing antibody. Proc. Natl. Acad. Sci. USA *110*, 6049–6054.

Scheid, J.F., Mouquet, H., Ueberheide, B., Diskin, R., Klein, F., Oliveira, T.Y., Pietzsch, J., Fenyo, D., Abadir, A., Velinzon, K., et al. (2011). Sequence and structural convergence of broad and potent HIV antibodies that mimic CD4 binding. Science *333*, 1633–1637.

Shingai, M., Donau, O.K., Plishka, R.J., Buckler-White, A., Mascola, J.R., Nabel, G.J., Nason, M.C., Montefiori, D., Moldt, B., Poignard, P., et al. (2014). Passive transfer of modest titers of potent and broadly neutralizing anti-HIV monoclonal antibodies block SHIV infection in macaques. J. Exp. Med. *211*, 2061–2074.

Sievers, F., and Higgins, D.G. (2014). Clustal omega, accurate alignment of very large numbers of sequences. In Multiple Sequence Alignment Methods, D.J. Russell, ed. (Totowa, NJ: Humana Press), pp. 105–116.

Sok, D., Briney, B., Jardine, J.G., Kulp, D.W., Menis, S., Pauthner, M., Wood, A., Lee, E.C., Le, K.M., Jones, M., et al. (2016). Priming HIV-1 broadly neutralizing antibody precursors in human Ig loci transgenic mice. Science *353*, 1557–1560.

Stewart-Jones, G.B., Soto, C., Lemmin, T., Chuang, G.Y., Druz, A., Kong, R., Thomas, P.V., Wagh, K., Zhou, T., Behrens, A.J., et al. (2016). Trimeric HIV-1-Env structures define glycan shields from clades A, B, and G. Cell *165*, 813–826.

Tian, M., Cheng, C., Chen, X., Duan, H., Cheng, H.L., Dao, M., Sheng, Z., Kimble, M., Wang, L., Lin, S., et al. (2016). Induction of HIV neutralizing antibody lineages in mice with diverse precursor repertoires. Cell *166*, 1471–1484.e18.

Victora, G.D., and Nussenzweig, M.C. (2012). Germinal centers. Annu. Rev. Immunol. *30*, 429–457.

Weaver, G.C., Villar, R.F., Kanekiyo, M., Nabel, G.J., Mascola, J.R., and Lingwood, D. (2016). In vitro reconstitution of B cell receptor-antigen interactions to evaluate potential vaccine candidates. Nat. Protoc. *11*, 193–213.

West, A.P., Jr., Diskin, R., Nussenzweig, M.C., and Bjorkman, P.J. (2012). Structural basis for germ-line gene usage of a potent class of antibodies targeting the CD4-binding site of HIV-1 gp120. Proc. Natl. Acad. Sci. USA *109*, E2083–E2090.

Wiehe, K., Bradley, T., Meyerhoff, R.R., Hart, C., Williams, W.B., Easterhoff, D., Faison, W.J., Kepler, T.B., Saunders, K.O., Alam, S.M., et al. (2018). Functional relevance of improbable antibody mutations for HIV broadly neutralizing antibody development. Cell Host Microbe *23*, 759–765.e6.

Wu, X., Yang, Z.Y., Li, Y., Hogerkorp, C.M., Schief, W.R., Seaman, M.S., Zhou, T., Schmidt, S.D., Wu, L., Xu, L., et al. (2010). Rational design of envelope identifies broadly neutralizing human monoclonal antibodies to HIV-1. Science *329*, 856–861.

Wu, X., Zhou, T., Zhu, J., Zhang, B., Georgiev, I., Wang, C., Chen, X., Longo, N.S., Louder, M., McKee, K., et al.; NISC Comparative Sequencing Program (2011). Focused evolution of HIV-1 neutralizing antibodies revealed by structures and deep sequencing. Science *333*, 1593–1602.

Wu, X., Zhang, Z., Schramm, C.A., Joyce, M.G., Kwon, Y.D., Zhou, T., Sheng, Z., Zhang, B., O'Dell, S., McKee, K., et al.; NISC Comparative Sequencing Program (2015). Maturation and diversity of the VRC01-antibody lineage over 15 years of chronic HIV-1 infection. Cell *161*, 470–485.

Yacoob, C., Pancera, M., Vigdorovich, V., Oliver, B.G., Glenn, J.A., Feng, J., Sather, D.N., McGuire, A.T., and Stamatatos, L. (2016). Differences in allelic frequency and CDRH3 region limit the engagement of HIV Env immunogens by putative VRC01 neutralizing antibody precursors. Cell Rep. *17*, 1560–1570.

Zhou, T., Georgiev, I., Wu, X., Yang, Z.Y., Dai, K., Finzi, A., Kwon, Y.D., Scheid, J.F., Shi, W., Xu, L., et al. (2010). Structural basis for broad and potent neutralization of HIV-1 by antibody VRC01. Science *329*, 811–817.

Zhou, T., Zhu, J., Wu, X., Moquin, S., Zhang, B., Acharya, P., Georgiev, I.S., Altae-Tran, H.R., Chuang, G.Y., Joyce, M.G., et al.; NISC Comparative Sequencing Program (2013). Multidonor analysis reveals structural elements, genetic determinants, and maturation pathway for HIV-1 neutralization by VRC01-class antibodies. Immunity *39*, 245–258.

Zhou, T., Lynch, R.M., Chen, L., Acharya, P., Wu, X., Doria-Rose, N.A., Joyce, M.G., Lingwood, D., Soto, C., Bailer, R.T., et al.; NISC Comparative Sequencing Program (2015). Structural repertoire of HIV-1-neutralizing antibodies targeting the CD4 supersite in 14 donors. Cell *161*, 1280–1292.

STAR★METHODS

KEY RESOURCES TABLE

REAGENT or RESOURCE	SOURCE	IDENTIFIER
Antibodies		
VRC03 g (VRC01 lineage mAb isolated from individual NIH45)	Produced in house (Wu et al., 2015)	GenBank: KP840675, KP840706
VRC03 (VRC01 lineage mAb isolated from individual NIH45)	Produced in house (Wu et al., 2015)	GenBank: GU980706, GU980707; RRID: AB_2491021
VRC03b (VRC01 lineage mAb isolated from individual NIH45)	Produced in house (Wu et al., 2015)	GenBank: KP840671, KP840702
VRC03f (VRC01 lineage mAb isolated from individual NIH45)	Produced in house (Wu et al., 2015)	GenBank: KP840674, KP840705
VRC03e (VRC01 lineage mAb isolated from individual NIH45)	Produced in house (Wu et al., 2015)	GenBank: KP840673, KP840704
VRC03i (VRC01 lineage mAb isolated from individual NIH45)	Produced in house (Wu et al., 2015)	GenBank: KP840677, KP840708
VRC03h (VRC01 lineage mAb isolated from individual NIH45)	Produced in house (Wu et al., 2015)	GenBank: KP840676, KP840707
VRC03d (VRC01 lineage mAb isolated from individual NIH45)	Produced in house (Wu et al., 2015)	GenBank: KP840672, KP840703
VRC06b (VRC01 lineage mAb isolated from individual NIH45)	Produced in house (Li et al., 2012)	GenBank: JX466925, JX466926
VRC06d (VRC01 lineage mAb isolated from individual NIH45)	Produced in house (Wu et al., 2015)	GenBank: KP840679, KP840710
VRC06 g (VRC01 lineage mAb isolated from individual NIH45)	Produced in house (Wu et al., 2015)	GenBank: KP840682, KP840713
VRC06e (VRC01 lineage mAb isolated from individual NIH45)	Produced in house (Wu et al., 2015)	GenBank: KP840680, KP840711
VRC06f (VRC01 lineage mAb isolated from individual NIH45)	Produced in house (Wu et al., 2015)	GenBank: KP840681, KP840712
VRC06 (VRC01 lineage mAb isolated from individual NIH45)	Produced in house (Li et al., 2012)	GenBank: JX466923, JX466924
VRC06c (VRC01 lineage mAb isolated from individual NIH45)	Produced in house (Wu et al., 2015)	GenBank: KP840678, KP840709
VRC08e (VRC01 lineage mAb isolated from individual NIH45)	Produced in house (Wu et al., 2015)	GenBank: KP840687, KP840718
VRC08c (VRC01 lineage mAb isolated from individual NIH45)	Produced in house (Wu et al., 2015)	GenBank: KP840685, KP840716
VRC08d (VRC01 lineage mAb isolated from individual NIH45)	Produced in house (Wu et al., 2015)	GenBank: KP840686, KP840717
VRC08 (VRC01 lineage mAb isolated from individual NIH45)	Produced in house (Wu et al., 2015)	GenBank: KP840684, KP840715
VRC02 (VRC01 lineage mAb isolated from individual NIH45)	Produced in house (Wu et al., 2015)	GenBank: GU980704, GU980705; RRID: AB_2491020
VRC01c (VRC01 lineage mAb isolated from individual NIH45)	Produced in house (Wu et al., 2015)	GenBank: KP840658, KP840689
VRC01g (VRC01 lineage mAb isolated from individual NIH45)	Produced in house (Wu et al., 2015)	GenBank: KP840662, KP840693
VRC01b (VRC01 lineage mAb isolated from individual NIH45)	Produced in house (Wu et al., 2015)	GenBank: KP840657, KP840688
VRC01 (VRC01 lineage mAb isolated from individual NIH45)	Produced in house (Wu et al., 2015)	GenBank: GU980702, GU980703; RRID: AB_2491019

(*Continued on next page*)

Continued

REAGENT or RESOURCE	SOURCE	IDENTIFIER
VRC01i (VRC01 lineage mAb isolated from individual NIH45)	Produced in house (Wu et al., 2015)	GenBank: KP840664, KP840695
VRC01h (VRC01 lineage mAb isolated from individual NIH45)	Produced in house (Wu et al., 2015)	GenBank: KP840663, KP840694
VRC01f (VRC01 lineage mAb isolated from individual NIH45)	Produced in house (Wu et al., 2015)	GenBank: KP840661, KP840692
VRC01e (VRC01 lineage mAb isolated from individual NIH45)	Produced in house (Wu et al., 2015)	GenBank: KP840660, KP840691
VRC01j (VRC01 lineage mAb isolated from individual NIH45)	Produced in house (Wu et al., 2015)	GenBank: KP840665, KP840696
VRC01d (VRC01 lineage mAb isolated from individual NIH45)	Produced in house (Wu et al., 2015)	GenBank: KP840659, KP840690
VRC07e (VRC01 lineage mAb isolated from individual NIH45)	Produced in house (Wu et al., 2015)	GenBank: KP840669, KP840700
VRC07f (VRC01 lineage mAb isolated from individual NIH45)	Produced in house (Wu et al., 2015)	GenBank: KP840670, KP840701
VRC07d (VRC01 lineage mAb isolated from individual NIH45)	Produced in house (Wu et al., 2015)	GenBank: KP840668, KP840699
NIH45-46 (VRC01 lineage mAb isolated from individual NIH45)	Produced in house (Scheid et al., 2011)	GenBank: HE584543, HE584544; RRID: AB_2491035
VRC07c (VRC01 lineage mAb isolated from individual NIH45)	Produced in house (Wu et al., 2015)	GenBank: KP840667, KP840698
VRC07b (VRC01 lineage mAb isolated from individual NIH45)	Produced in house (Wu et al., 2015)	GenBank: KP840666, KP840697
VRC01 V.Rev	Wu et al., 2010	See Table S1
VRC03 V.Rev	This paper	See Table S1
VRC18b V.Rev	This paper	See Table S1
3BNC60 V.Rev	This paper	See Table S1
3BNC117 V.Rev	This paper	See Table S1
12A12 V.Rev	This paper	See Table S1
VRC20 V.Rev	This paper	See Table S1
VRC23 V.Rev	This paper	See Table S1
VRC01 VJ.Rev (aka VRC01 GL.Rev)	Jardine et al., 2016a	See Table S1
VRC03 VJ.Rev (aka VRC03 GL.Rev)	Jardine et al., 2016a	N/A
VRC06 VJ.Rev (aka VRC06 GL.Rev)	Jardine et al., 2016a	N/A
NIH-45-46 VJ.Rev (aka NIH45-46 GL.Rev)	Jardine et al., 2016a	N/A
Bacterial and Virus Strains		
HIV-1 MN.3 pseudovirus	Produced in house.	Los Alamos Databases. HIV sequence database Accession number HM215430
HIV-1 strain BJOX2000 pseudovirus	Produced in house (deCamp et al., 2014)	NIH AIDS Reagent Program Cat. No. 12670
HIV-1 strain CE1176 pseudovirus	Produced in house (deCamp et al., 2014)	NIH AIDS Reagent Program Cat. No. 12670
HIV-1 strain X1632 pseudovirus	Produced in house (deCamp et al., 2014)	NIH AIDS Reagent Program Cat. No. 12670
HIV-1 strain X2278 pseudovirus	Produced in house (deCamp et al., 2014)	NIH AIDS Reagent Program Cat. No. 12670
HIV-1 strain 398F1 pseudovirus	Produced in house (deCamp et al., 2014)	NIH AIDS Reagent Program Cat. No. 12670
HIV-1 strain 25710 pseudovirus	Produced in house (deCamp et al., 2014)	NIH AIDS Reagent Program Cat. No. 12670
HIV-1 strain CNE8 pseudovirus	Produced in house (deCamp et al., 2014)	NIH AIDS Reagent Program Cat. No. 12670
HIV-1 strain TRO11 pseudovirus	Produced in house (deCamp et al., 2014)	NIH AIDS Reagent Program Cat. No. 12670
HIV-1 strain 246F3 pseudovirus	Produced in house (deCamp et al., 2014)	NIH AIDS Reagent Program Cat. No. 12670
HIV-1 strain CE0217 pseudovirus	Produced in house (deCamp et al., 2014)	NIH AIDS Reagent Program Cat. No. 12670
HIV-1 strain CH119 pseudovirus	Produced in house (deCamp et al., 2014)	NIH AIDS Reagent Program Cat. No. 12670
HIV-1 strain CNE55 pseudovirus	Produced in house (deCamp et al., 2014)	NIH AIDS Reagent Program Cat. No. 12670

(*Continued on next page*)

Continued		
REAGENT or RESOURCE	SOURCE	IDENTIFIER
Biological Samples		
PBMCs from patient NIH45	VRC/NIAID	N/A
Chemicals, Peptides, and Recombinant Proteins		
Synthetic construct eOD-GT6-Avi-3C-His (eOD-GT6)	Produced in house (Tian et al., 2016)	GenBank: KX527852
Synthetic construct delta_eOD-GT6-Avi-3C-His gene (eOD-GT6 KO)	Produced in house (Tian et al., 2016)	GenBank: KX527853
Synthetic construct eOD-GT8-Avi-3C-His gene (eOD-GT8) (monomer)	Produced in house (Tian et al., 2016)	GenBank: KX527855
Synthetic construct delta_eOD-GT8-Avi-3C-His (eOD-GT8 KO) (monomer)	Produced in house (Tian et al., 2016)	GenBank: KX527856
Synthetic construct clone 426c-degly3.coreE-AviHis mutant envelope glycoprotein (TM1ΔV1-3)	Produced in house (Tian et al., 2016)	GenBank: KX518319
Synthetic construct clone 426c-degly3.D279K/D368R.coreE-AviHis mutant envelope glycoprotein (TM1ΔV1-3 KO)	Produced in house (Tian et al., 2016)	GenBank: KX518320
Synthetic construct chimeric gp120 core C13.G3 precursor (C13)	Produced in house (Tian et al., 2016)	GenBank: KX462845
Synthetic construct chimeric gp120 core C13.G3 D279K precursor (C13 KO)	Produced in house (Tian et al., 2016)	N/A
eOD-GT8 (heptamer)	Produced in house.	N/A
eOD-GT8 (24-mer)	Produced in house.	N/A
TM4ΔV1-3 (monomer)	Produced in house (McGuire et al., 2016)	N/A
TM4ΔV1-3 KO (monomer)	Produced in house (McGuire et al., 2016)	N/A
TM4ΔV1-3 (heptamer)	Produced in house (McGuire et al., 2016)	N/A
TM4ΔV1-3 D368R/E370A (heptamer)	Produced in house (McGuire et al., 2016)	N/A
TM4ΔV1-3 (24-mer)	Produced in house (McGuire et al., 2016)	N/A
TM4ΔV1-3 D368R/E370A (24-mer)	Produced in house (McGuire et al., 2016)	N/A
BG505 SOSIP v4.1-GT1	Produced in house (Medina-Ramírez et al., 2017)	N/A
BG505 SOSIP	Produced in house (Medina-Ramírez et al., 2017)	N/A
Resurfaced Core Protein-3 (RSC3)	Produced in house (Wu et al., 2010)	N/A
Resurfaced Core Protein-3 delta 371I/P363N	Produced in house (Wu et al., 2010)	N/A
UBE3A	UBPBio	Cat No. K1410
SureBlue Reserve TMB	KPL	Cat. No. 53-00-03
Jo-1 antigen	ImmunoVision	Cat. No. JO1-3000
nRNP Complex (Sm/RNP)	ImmunoVision	Cat. No. SRC-3000
Scl-70 antigen	ImmunoVision	Cat. No. SCL-3000
Smith (Sm) antigen	ImmunoVision	Cat. No. SMA-3000
SSA (Ro) antigen	ImmunoVision	Cat. No. SSA-3000
SSB (La) antigen	ImmunoVision	Cat. No. SSB-3000
Centromere protein B	Prospec	Cat. No. PRO-390
Deoxyribonucleic Acid, Calf Thymus Source	Worthington	Cat. No. LS002105
Poly-lysine	Sigma Aldrich	Cat. No. P6285
Critical Commercial Assays		
ExpiFectamine 293 transfection kit	ThermoFisher Scientific	Cat. No. A14525
FLIPR Calcium 6 Assay Kit	Molecular Devices	Cat. No. R8190

(*Continued on next page*)

Continued

REAGENT or RESOURCE	SOURCE	IDENTIFIER
ANA HEp-2 test system	Zeuss Scientific	Cat. No. FA2400
ProtoArray Human Protein Microarray	Invitrogen	Cat. No. PAH0525101
Deposited Data		
VRC01 UCA	GenBank	MK032222, MK032237
DH651.1 (VRC01 lineage mAb isolated from individual NIH45)	GenBank	MK032223, MK032238
DH651.2 (VRC01 lineage mAb isolated from individual NIH45)	GenBank	MK032224, MK032239
DH651.3 (VRC01 lineage mAb isolated from individual NIH45)	GenBank	MK032225, MK032240
DH651.4 (VRC01 lineage mAb isolated from individual NIH45)	GenBank	MK032226, MK032241
DH651.5 (VRC01 lineage mAb isolated from individual NIH45)	GenBank	MK032227, MK032242
DH651.6 (VRC01 lineage mAb isolated from individual NIH45)	GenBank	MK032228, MK032243
DH651.7 (VRC01 lineage mAb isolated from individual NIH45)	GenBank	MK032229, MK032244
DH651.8 (VRC01 lineage mAb isolated from individual NIH45)	GenBank	MK032230, MK032245
DH651.9 (VRC01 lineage mAb isolated from individual NIH45)	GenBank	MK032231, MK032246
Experimental Models: Cell Lines		
MS40L cells	Luo et al., 2009	N/A
Ramos cells	Benjamin et al., 1982	ATCC CRL-1596
TZM-bl cells	Dr. John C. Kappes and Dr. Xiaoyun Wu	NIH AIDS Reagent Program Cat. No. 8129
Oligonucleotides		
gggcttctggatatgaatttattgattgttatctaaa ttggattcgtctggcccc	ThermoFisher	N/A
ggatatgaatttattgattgtacgatgaattggattc gtctggcccccc	ThermoFisher	N/A
Software and Algorithms		
Cloanalyst	Kepler, 2013	https://www.bu.edu/computationalimmunology/research/software/
Clustal Omega	Sievers and Higgins, 2014	https://www.ebi.ac.uk/Tools/msa/clustalo/
PhyML	Guindon et al., 2010	www.phylogeny.fr
DNAML	Felsenstein, 2005	http://evolution.genetics.washington.edu/phylip/doc/dnaml.html
BioEdit v 7.1.3.0	Hall, 1999	http://www.mbio.ncsu.edu/BioEdit/bioedit.html
EMBOSS Water	EMBL-EBI	https://www.ebi.ac.uk/Tools/psa/emboss_water/
Biacore S200 Evaluation Software	GE Healthcare	https://www.biacore.com
Modeler	Sali and Blundell, 1993	https://salilab.org/modeller
Rosetta	RosettaCommons	https://rosettacommons.org
NAMD 2.12	Phillips et al., 2005	http://www.ks.uiuc.edu/Research/namd/
GenePix Pro 5.0	Molecular Devices	https://www.moleculardevices.com
SoftMax Pro 5.4.1	Molecular Devices	https://www.moleculardevices.com
ARMADiLLO	Wiehe et al., 2018	Inquiries to K. Wiehe of the DHVI

(*Continued on next page*)

Continued		
REAGENT or RESOURCE	SOURCE	IDENTIFIER
Prism v 7.03	GraphPad	https://www.graphpad.com
Analyze Align Tool	Los Alamos HIV database web interface	https://www.hiv.lanl.gov/content/sequence/ANALYZEALIGN/analyze_align.html
Other		
Biacore S200	GE Healthcare	N/A
FlexStation 3	Molecular Devices	N/A
Olympus AX70 fluorescent microscope	Olympus	N/A
GenePix 4000B scanner	Molecular Devices	N/A
SpectraMax 384PLUS	Molecular Devices	N/A
Biomek FX	Beckman Coulter	N/A

CONTACT FOR REAGENT AND RESOURCE SHARING

Further information and requests for resources and reagents should be directed to and will be fulfilled by the Lead Contact, Mattia Bonsignori (mattia.bonsignori@duke.edu) and the Senior Author, Barton F. Haynes (barton.haynes@duke.edu).

EXPERIMENTAL MODEL AND SUBJECT DETAILS

Human specimens

The peripheral blood mononuclear cells (PBMCs) used to isolate mAbs DH651.1 through DH651.9 were collected from NIH donor 45, from whom the VRC01 lineage was isolated (Wu et al., 2010). Donor 45 is a male diagnosed with a clade B virus infection in 1990. He enrolled in a clinical protocol approved by the Institutional Review Board at the National Institute of Allergy and Infectious Diseases in 1995. PBMCs used in this study were collected in 2008, 18 years after diagnosis. Donor 45 is a slow progressor who, from 1995 to 2009, maintained $CD4^{+}$ T cell counts over 500 cells/μl and plasma HIV-1 RNA values less than 17,000 copies/ml without ever receiving antiretroviral treatment (Wu et al., 2010; Wu et al., 2015).

Cell lines

MS40L is a cell line derived from a murine stem cell line (MS5) to express low levels of cell surface human CD40L. MS40L cells have been widely used to support robust B cell growth *in vitro* in presence of additional stimulants. Information on the sex of the mouse from which the MS5 cell line was derived is not available.

TZM-bl is a HeLa cell line that expresses CD4 receptor and both CXCR4 and CCR5 chemokine co-receptors; TZM-bl cells also express luciferase and β-galactosidase under the control of the HIV-1 promoter, hence are useful to assay *in vitro* HIV-1 infection. The HeLa cell line was derived from cancerous epithelial cells isolated from the cervix of a patient with cervical adenocarcinoma.

Expi293F cells (ThermoFisher) are human cells derived from the 293F cell line, which were, in turn, derived from transformed human embryonal kidney (HEK) cells. HEK293 is a hypotriploid cell line containing three copies of X chromosomes and no evidence of Y chromosome-derived sequences, suggesting that the fetus from which they were derived was female. Expi293F cells were transfected with IgH and IgL-encoding plasmids to produce recombinant monoclonal antibodies.

Ramos is a cell line derived from human lymphoblasts of a young male with Burkitt's lymphoma: Ramos cells were stably transfected to express IgM BCRs of interest.

The human epithelial type 2 (HEp-2) cell line, initially considered to originate from a human laryngeal carcinoma, has been later authenticated to derive from HeLa cells: HEp-2 cells enable the identification of antinuclear antibodies.

METHOD DETAILS

Isolation of VRC01 lineage antibodies

The HIV-1 Resurfaced Core Protein-3 (RSC3) (Wu et al., 2010) was produced and used in flow cytometry on 39 million PBMCs collected from donor 45 using a two-color technique as described (Gray et al., 2011). A total of 1,917 RSC3-positive memory B cells were cultured as described (Bonsignori et al., 2016) with the following modifications: After overnight incubation in bulk in presence with EBV, memory B cells were plated at limiting dilution (0.8 cells/well) in culture wells containing MS40L feeder cells (Luo et al., 2009) irradiated (9,000 cGy) and plated at a concentration of 5,000 cells/well. After 2 weeks, cell culture supernatants were screened for neutralization of the MN.3 HIV-1 strain using the tzm-bl cell-based neutralization assay (Bonsignori et al., 2011; Montefiori, 2005), and binding to consensus S gp140 Env, UBE3A, RSC3 and RSC3Δ371I/P363N (Liu et al., 2015; Wu et al., 2010). VRC01 lineage mAbs

DH651.1 through DH651.9 were isolated from culture supernatants that displayed differential binding of RSC3 and RSC3Δ371I/P363N; of them, UBE3A reactivity was detected in 3 cultures, and 8 cultures neutralized > 75% MN.3 infectivity.

Antibody production

Immunoglobulin genes of mAbs DH651.1 through DH651.9 were amplified from RNA from isolated cells, expression cassettes made, and mAbs expressed as described (Gao et al., 2014; Liao et al., 2009). Heavy chain plasmids were co-transfected with appropriate light chain plasmids at an equal ratio in Expi293 cells using ExpiFectamine 293 transfection reagents (Thermo Fisher Scientific) according to the manufacturer's protocols and using the enhancer provided with the kit. Transfected cultures were incubated at 37°C 8% CO_2 for 2-6 days, harvested, concentrated and incubated overnight with Protein A beads at 4°C on a rotating shaker before loading the bead mixture in columns for purification; following PBS/NaCl wash, eluate was neutralized with trizma hydrochloride and antibody concentration was determined by Nanodrop. Purified antibodies were tested in SDS-Page Coomassie and western blots, and stored at 4°C. Thirty-six additional monoclonal antibodies in the VRC01 lineage for which naturally paired IgH and IgL sequences were previously described (Li et al., 2012; Scheid et al., 2011; Wu et al., 2010; Wu et al., 2015) were produced using the same method. Amino acid positions are expressed using the Kabat numbering system (Kabat et al., 1991). Alignments were performed using Bioedit and EMBOSS Water. (https://www.ebi.ac.uk/Tools/psa/emboss_water/).

Inference of the VRC01 clonal history including UCAs and unobserved intermediates

The hybrid method used to infer the VRC01 UCA and the unobserved IAs is as follows. First, we use our software Cloanalyst (Kepler, 2013; Liao et al., 2013) to verify the clonal relatedness of the candidate members. Briefly, Cloanalyst computes the summed log-marginal likehoods over subsets of the candidate sequences, each subset treated as an independent clone, and identifies that subset that maximizes that quantity. That subset, in this case, was the complete set, indicating that all observed members belong to the same clone.

We then performed a multiple sequence alignment using Clustal Omega (Sievers and Higgins, 2014), and after manual adjustment of the resulting gaps to minimize the number of columns containing gaps, we removed all columns containing gaps. We then used PhyML (Guindon et al., 2010), provided online by the server at http://www.phylogeny.fr/, using the HKY85 substitution model, to infer the maximum likelihood trees for the heavy- and light-chain collections separately. We performed 100 bootstrap replicates to identify weakly supported branches. We then concatenated the heavy- and light-chain sequences from each observed antibody, leaving the inferred gaps intact, and used Phylip's DNAML (Felsenstein, 2005) to estimate the maximum likelihood tree common to both heavy and light chains. We used five classes of mutation rates in the discrete-gamma model of rate variation, and a base rate in which the heavy chain mutates at twice the rate of the light chain. We then computed the UCA and the unobserved IAs using Cloanalyst, performing a Bayesian average over all viable combinations of rearrangement parameters with prior distributions on recombination parameters obtained using data from an independent study. We then manually readjusted the precise locations of the indels to account for the locations of the recombination points in the most-probable gene segments involved in the UCA. We recomputed the joint maximum-likelihood tree and the heavy- and light-chain UCAs.

Cloanalyst estimates the expected error in each base of the inferred UCAs conditional on the phylogenetic tree and on the multiple sequence alignment, i.e., the positions of the indels. Since in this case, both the phylogenetic tree and the multiple sequence alignment are subject to non-negligible uncertainty, the estimates of the expected errors should be regarded as minimum estimates. If we were able to average over trees and alignments as well, these expected errors might be substantially larger.

Surface Plasmon Resonance Affinity and Kinetics Measurements

Dissociation (K_D) and rate constants (k_{on}, k_{off}) were measured using the Biacore S200 (GE Healthcare). CM5 sensor chips (or CM3 for SOSIP proteins) were used to directly immobilize antibodies to a level of approximately 2000-3000RU. Proteins were diluted from 0.5nM-4000nM (0.1 μg/mL-150 μg/mL) in HBS-EP+ 1 × buffer and then injected over the antibody immobilized surfaces for 5 min at 50 μL/min. The 5 min analyte injection was followed by a 10 min dissociation period with buffer wash and then a 20 s injection pulse of Glycine pH2.0 for regeneration. Kinetics results were analyzed using the Biacore S200 Evaluation Software (GE Healthcare). A negative control antibody (Ab82) and buffer binding were used for double reference subtraction to account for non-specific binding and signal drift. Subsequent curve fitting analysis was performed using a 1:1 Langmuir model with a local R_{max} and the reported rate constants are representative of 2 measurements. Exceptions to the above curve fitting model included some BG505 trimeric protein interactions, which were analyzed using the bivalent analyte model and for some core protein interactions, which were analyzed using the heterogeneous ligand model.

Biolayer Interferometry (BLI)

BLI assays were performed on the Octet Red instrument at 30°C with shaking at 1,000 RPM. Anti-Human IgG Fc capture (AHC) biosensors (Fortebio) were immersed into PBS containing 20μg/ml VRC01 UCA IgG for 240 s. A baseline signal was recorded for 1 min in kinetics buffer (KB: 1X PBS, 0.01% BSA, 0.02% Tween 20, and 0.005% NaN_3, at pH 7.4). Sensors were then immersed into solutions containing 2μM of monomeric, heptameric, or icositetrameric eOD-GT8, eODGT8 D279K/D368R, 426c TM4ΔV1-3 or 426c TM4ΔV1-3 D368R/E370A glycoprotein for 300 s to measure association, followed by immersion in KB for 300 s to measure dissociation. All measurements of antibody binding were corrected by subtracting the signal obtained from simultaneous traces performed with the corresponding envelopes in the absence of antibody, using PBS only.

Calcium Flux Measurement

Proteins expressed with a C-terminal avidin tag sequence (GLNDIFEAQKIEWHE) were biotinylated with the BirA biotin-protein ligation kit (Avidity) and agitated at 900 rpm 30°C for 5 h. The biotinylated protein was then transferred to a 0.5mL 3kDa MWCO spin column (Amicon) and excess biotin was removed with five washes of PBS (GIBCO). Protein tetramers were formed at a 4:1 molar ratio of protein to streptavidin (Invitrogen). To maximize streptavidin site occupancy 1/5 of the streptavidin volume was added 5 × every 15 min. Molarity was calculated based upon the number of moles of protein added to the tetramer reaction. Cultured stably transfected Ramos cell lines (Benjamin et al., 1982; Weaver et al., 2016) were passaged 1:10 four days preceding calcium flux experiments. On the day of the experiment cells with > 95% viability were resuspended at 1x10^6 cells/mL in 2:1 ratio of RPMI media (GIBCO) + FLIPR Calcium 6 dye (Molecular Devices). Cells were plated in a U-bottom 96 well tissue culture plate (Costar) and incubated at 37°C 5% CO_2 for 2 h. In a black clear bottom 96 well plate (Costar) containing 50 μLs RPMI media (GIBCO) + FLIPR Calcium 6 dye (Molecular Devices) (2:1 ratio) either 0.1nMoles of proteins or 50 μg/mL of Anti-human IgM F(ab')$_2$ (Jackson Immuno) were added (based on a 100 μL volume). Using a FlexStation 3 multi-mode microplate reader (Molecular Devices) 50 μL of supernatant containing cells were transferred into the 50 μL of media containing protein or Anti-human IgM F(ab')$_2$ (Jackson Immuno) and continuously read for 5 min. Relative fluorescent value units were background subtracted and the data expressed as percentage of the IgM maximum signal (% IgM_{max}).

Assessment of virus neutralization

Antibody neutralization was measured in TZM-bl cell-based assays (Montefiori, 2005). Neutralization breadth was assessed using a 12-virus panel that recapitulates global HIV-1 diversity (deCamp et al., 2014). Data were calculated as a reduction in luminescence units compared with control wells, and reported as IC_{50} or IC_{80} in μg/ml.

Immunogens

The following immunogens were produced at the Duke Human Vaccine Institute and at the VRC/NIAID with plasmids prepared at the VRC/NIAID: eOD-GT6 (KX527852) (Tian et al., 2016), eOD-GT6 KO (KX527854) (Tian et al., 2016), eOD-GT8 (KX527855) (Tian et al., 2016), eOD-GT8 KO (KX527856) (Tian et al., 2016), TM1ΔV1-3 (KX518319) (Tian et al., 2016), TM1ΔV1-3 KO (KX518320) (Tian et al., 2016), C13 (KX462845) (Tian et al., 2016) and C13 KO was made by introducing the D279K mutation in C13. TM4ΔV1-3 and TM4ΔV1-3 KO were produced at the Vaccine and Infectious Disease Division of the Fred Hutchinson Cancer Research Center as described (McGuire et al., 2016). BG505 SOSIP v4.1-GT1 and wild-type BG505 SOSIP were produced at the Department of Medical Microbiology of the Academic Medical Center (University of Amsterdam) as described (Medina-Ramírez et al., 2017).

Molecular Dynamics Simulation

A gp120 portion from the crystal structure of the VRC08 Fab structure in complex with HIV-1 strain Q842.d12 gp120 (PDB: 4XMP) was aligned with each gp120 of the crystal structure of JR-FL SOSIP trimer in complex with PGT122, 35O22 and VRC01 (PDB: 5FYK) after addition of missing loops in the JR-FL structure using Modeler (Sali and Blundell, 1993; Stewart-Jones et al., 2016; Wu et al., 2015). The Q842.d12 gp120 portions were removed along with all antibodies excluding VRC08. The JR-FL structure was then glycosylated with Man5 using Rosetta according to glycan positions determined using the LANL N-GlycoSite web server. The glycosylated timer-antibody complex was then minimized in vacuum using the CHARMM36 force field for 25,000 steps with the protein backbone atoms fixed using NAMD 2.12 (Phillips et al., 2005). The minimized complex was then solvated in TIP3P water molecules with the addition of neutralizing NaCl brought to an effective concentration of 0.150M using VMD to give a total system size of 890,399 atoms (Humphrey et al., 1996; Jorgensen et al., 1983). The solvent protein system was then minimized for 1,500 steps followed by 50 ps of dynamics using a 1 fs time step with constraints on the protein backbone atoms using a constraint exponent of two. The system was then minimized for 1,500 steps followed by heating from 50 K to 300 K with the constraints removed followed by 250 ps of dynamics at 300K. The protein-solvent system was then simulated unconstrained for a total of 50 ns (ns) using a 2fs time step with hydrogens constrained using the SHAKE algorithm (Ryckaert et al., 1977). The temperature was maintained using Langevin dynamics with a damping coefficient of 1/ps with the pressure maintained at 1 atm using the Nosé-Hoover Langevin position method with a period of 100 fs and decay of 50 fs (Martyna et al., 1994). Electrostatic and van der Waals interaction calculations were cut off at 12 Å using switching functions beginning at 10 Å with long range electrostatic calculations handled using the particle mesh Ewald method with periodic boundary conditions (Essmann et al., 1995). Visualization and analysis of the resulting trajectories was performed using VMD and plugins therein. Specifically, the RMSD of the CDR H3, sequence GRSCCGGRRHCNGADCFNWDFQH, was determined for each of the three bound VRC08 Fabs using the RMSD trajectory tool (Humphrey et al., 1996).

HEp-2 Cell Staining

Indirect immunofluorescence binding of mAbs to HEp-2 cells (Zeuss Scientific) was performed as previously described (Bonsignori et al., 2014; Haynes et al., 2005). Briefly, 20μl of antibody at 50μg/ml was aliquoted onto a predetermined spot on the surface of a slide (ANA HEp-2 kit). After incubation for 20 min at room temperature and washes, 20μl of secondary antibody (goat anti-human Ig FITC at 30μg/ml; Southern Biotech) was added to each spot and incubated in a humid chamber for 20 min in the dark. After washing and drying, a drop of 50% glycerol was added to each spot, and the slide was covered with a 24- by 60-mm coverslip. Images were taken on an Olympus AX70 instrument with a SpotFlex FX1520 CCD and with a UPlanFL 40× 0.75-NA objective at 25°C in the FITC channel using SPOT software. All images were acquired for 12s. Image layout and scaling were performed with Adobe Photoshop without image manipulation.

Protein Array

MAbs were screened for binding on protein microarrays (ProtoArray) (PAH0525101; Invitrogen) pre-coated with 9,400 human proteins in duplicate and screened following manufacturer's instructions and as previously described (Liu et al., 2015). Briefly, after blocking, the microarray was incubated on ice with 2 μg/ml of mAbs or isotype control 151K for 90 min. Ab binding to array protein was detected with 1 μg/ml of Alexa Fluor 647-labeled anti-human IgG (Invitrogen) secondary Ab. Microarrays were scanned using a GenePix 4000B scanner (Molecular Devices) at a wavelength of 635 nm, with 10-μm resolution, using 100% power and 600 gain. Fluorescence intensities were quantified with GenePix Pro 5.0 program (Molecular Devices) using lot-specific protein location information provided by the microarray manufacturer.

Indirect-binding ELISA

ELISAs were performed as previously described (Bonsignori et al., 2017; Bonsignori et al., 2016). Briefly, for biotinylated proteins (i.e., biotinylated avi-tagged RSC3, RSC3Δ371I/P363N and consensus S gp140 Env), plates were coated with 2 μg streptavidin, incubated at RT for 2h and blocked either for 1h at RT or overnight at 4°C. Biotinylated proteins were added (2 μg/ml) for 30 mins at RT or overnight at 4°C. The autoantigen UBE3A (UBPBio catalog # K1410) was directly coated on the plates at 6ug/ml, incubated at RT for 2h and blocked overnight at 4°C. Culture supernatants were added at a 1:3 dilution in assay diluent, whereas purified antibodies were titrated starting at 100 μg/ml (1:3 dilutions, 11 steps), incubated for 1h and 45 mins at RT. After washing, HRP-conjugated goat anti-human IgG antibody (Jackson ImmunoResearch) was added at lot-specific predetermined optimal concentration for 1h; after washing, plates were developed using SureBlue Reserve TMB (KPL, Gaithersburg, MD) equilibrated at RT. Development was stopped after 10 min and plates were read at 450nm and 650nm (for background subtraction) wavelengths in a SpectraMax 384PLUS reader (MolecularDevices, Sunnyvale, CA).

Purified histones (whole), Jo-1, RNP/Sm, Scl-70, Sm, SSA (Ro), SSB (all from ImmunoVision) and centromere B (Prospec) were coated in sodium bicarbonate solution overnight at 4°C at optimal concentrations determined by lot-specific checkerboard with human-derived positive controls (ImmunoVision). For DNA, plates were pre-coated with 10μg/ml poly-lysine (Sigma-Aldrich) overnight at 4°C, washed 1 × with wash buffer (PBS/0.05% tween 20) and followed with DNA (LS002195, Worthington) at 20 μg/ml in saline sodium citrate buffer for 1h as determined in optimization assays. All plates were washed 1 × , blocked with 3%BSA/PBS for 1 h at RT and flicked/tapped dry. Antibodies, serially diluted in assay diluent (1% BSA/PBS/0.05% Tween-20) were incubated for 45 min followed by 2 × wash. Secondary antibody was added for 30 min, washed 4 × followed by TMB substrate (Sera Care Life Sciences). Reactions were stopped after 10 min.

Antibody site-directed mutagenesis

Site-directed mutagenesis of antibody genes was performed using the Quikchange II lightening multi-site-directed mutagenesis kit following manufacturer's protocol (Agilent). Mutant plasmid products were confirmed by single-colony sequencing. Primers used for introducing mutations were: VRC01 T33Y: ggcttctggatatgaatttattgattgttatctaaattggattcgtctggcccc; VRC01 L34M: ggatatgaatttatt gattgtacgatgaattggattcgtctggcccccc. VRC01, VRC02, DH651.2 and DH651.4 T33L/L34M double mutants were expressed directly from plasmids containing the double mutations, without site-directed mutagenesis. The probability of the Y33T and M34M mutations were determined using the computational program Antigen Receptor Mutation Analyzer for Detection of Low Likelihood Occurrences (ARMADiLLO) (Wiehe et al., 2018).

Logo plots

Logo plots were generated using the Los Alamos HIV database web interfaces (https://www.hiv.lanl.gov, version Dec. 2015, HEATMAP and Analyze Align).

QUANTIFICATION AND STATISTICAL ANALYSIS

For associations between neutralization breadth or potency and clade membership of VRC01 lineage mAbs (Figure 5), the Kruskal-Wallis and Dunn's multiple comparison tests were applied (GraphPad Prism version 7.03). Significance was evaluated at the alpha 0.05 level.

For associations between neutralization breadth or potency and auto-/polyreactivity (Figures 7B and 7C) and between UBE3A binding and presence of the ^{33}TL^{34} motif in IgH (Figure S6A), the Mann-Whitney U-test method was applied (GraphPad Prism version 7.03). Significance was evaluated at the alpha 0.05 level.

Correlations between levels of neutralization breadth or potency and polyreactivity (Figures 7D and 7E) (n = 43) were analyzed by Spearman correlation and significance was evaluated at the alpha 0.05 level.

DATA AND SOFTWARE AVAILABILITY

The IgH and IgL variable region sequences of VRC01 UCA and DH651.1 through DH651.9 monoclonal antibodies have been deposited in GenBank with accession numbers MK032222 through MK032231 and MK032237 through MK032246.

Immunity, Volume *49*

Supplemental Information

Inference of the HIV-1 VRC01 Antibody Lineage Unmutated Common Ancestor Reveals Alternative Pathways to Overcome a Key Glycan Barrier

Mattia Bonsignori, Eric Scott, Kevin Wiehe, David Easterhoff, S. Munir Alam, Kwan-Ki Hwang, Melissa Cooper, Shi-Mao Xia, Ruijun Zhang, David C. Montefiori, Rory Henderson, Xiaoyan Nie, Garnett Kelsoe, M. Anthony Moody, Xuejun Chen, M. Gordon Joyce, Peter D. Kwong, Mark Connors, John R. Mascola, Andrew T. McGuire, Leonidas Stamatatos, Max Medina-Ramírez, Rogier W. Sanders, Kevin O. Saunders, Thomas B. Kepler, and Barton F. Haynes

Table S1. IgH and IgL chain sequences of GL VRC01-class mAbs produced in this study. Related to Figure 2.

VRC01 VJ.Rev	VH/JH reverted	QVQLVQSGAEVKKPGASVKVSCKASGYTFTGYYMHWVRQAPGQGLEWMGWINPNSGGTNYAQKFQGRVTMTRDTSISTAY<br>MELSRLRSDDTAVYYCARGKNSDYNWDFQHWGQGTLVTVSS
	VL/JL reverted	EIVLTQSPATLSLSPGERATLSCRASQSVSSYLAWYQQKPGQAPRLLIYDASNRATGIPARFSGSGSGTDFTLTISSLEP<br>EDFAVYYCQQYEFFGQGTKLEIK
VRC01 V.Rev	VH reverted	QVQLVQSGAEVKKPGASVKVSCKASGYTFTGYYMHWVRQAPGQGLEWMGWINPNSGGTNYAQKFQGRVTMTRDTSISTAY<br>MELSRLRSDDTAVYYCARGKNCDYNWDFEHWGRGTPVIVSS
	VL reverted	EIVLTQSPGTLSLSPGERATLSCRASQSVSSSYLAWYQQKPGQAPRLLIYGASSRATGIPDRFSGSGSGTDFTLTISRLE<br>PEDFAVYYCQQYEFFGQGTKVQVD
VRC03 V.Rev	VH reverted	QVQLVQSGAEVKKPGASVKVSCKASGYTFTGYYMHWVRQAPGQGLEWMGWINPNSGGTNYAQKFQGRVTMTRDTSISTAY<br>MELSRLRSDDTAVYYCARRGSCDYCGDFPWQYWGQGTVVVVSS
	VL reverted	EIVLTQSPGTLSLSPGERATLSCRASQSVSSSYLAWYQQKPGQAPRLLIYGASSRATGIPDRFSGSGSGTDFTLTISRLE<br>PEDFAVYYCQQFEFFGLGSELEVH
3BNC60 V.Rev	VH reverted	QVQLVQSGAEVKKPGASVKVSCKASGYTFTGYYMHWVRQAPGQGLEWMGWINPNSGGTNYAQKFQGRVTMTRDTSISTAY<br>MELSRLRSDDTAVYYCARQRSDFWDFDVWGSGTQVTVSS
	VL reverted	DIQMTQSPSSLSASVGDRVTITCQASQDISNYLNWYQQKPGKAPKLLIYDASNLETGVPSRFSGSGSGTDFTFTISSLQP<br>EDIATYYCQVYEFIVPGTRLDLK
3BNC117 V.Rev	VH reverted	QVQLVQSGAEVKKPGASVKVSCKASGYTFTGYYMHWVRQAPGQGLEWMGWINPNSGGTNYAQKFQGRVTMTRDTSISTAY<br>MELSRLRSDDTAVYYCARQRSDYWDFDVWGSGTQVTVSS
	VL reverted	DIQMTQSPSSLSASVGDRVTITCQASQDISNYLNWYQQKPGKAPKLLIYDASNLETGVPSRFSGSGSGTDFTFTISSLQP<br>EDIATYYCQVYEFVVPGTRLDLK
12A12 V.Rev	VH reverted	QVQLVQSGAEVKKPGASVKVSCKASGYTFTGYYMHWVRQAPGQGLEWMGWINPNSGGTNYAQKFQGRVTMTRDTSISTAY<br>MELSRLRSDDTAVYYCARDGSGDDTSWHLDPWGQGTLVIVSS
	VL reverted	DIQMTQSPSSLSASVGDRVTITCQASQDISNYLNWYQQKPGKAPKLLIYDASNLETGVPSRFSGSGSGTDFTFTISSLQP<br>EDIATYYCAVLEFFGPGTKVEIK
VRC20 V.Rev	VH reverted	QVQLVQSGAEVKKPGASVKVSCKASGYTFTGYYMHWVRQAPGQGLEWMGWINPNSGGTNYAQKFQGRVTMTRDTSISTAY<br>MELSRLRSDDTAVYYCARRMRSQDREWDFQHWGQGTRIIVSS
	VL reverted	QSALTQPASVSGSPGQSITISCTGTSSDVGGYNYVSWYQQHPGKAPKLMIYEVSNRPSGVSNRFSGSKSGNTASLTISGL<br>QAEDEADYYCNAFEFFGGGTKLTVL
VRC23 V.Rev	VH reverted	QVQLVQSGAEVKKPGASVKVSCKASGYTFTGYYMHWVRQAPGQGLEWMGWINPNSGGTNYAQKFQGRVTMTRDTSISTAY<br>MELSRLRSDDTAVYYCARGVRRDASWWLQFWGQGTLVTVSS
	VL reverted	EIVMTQSPATLSVSPGERATLSCRASQSVSSNLAWYQQKPGQAPRLLIYGASTRATGIPARFSGSGSGTEFTLTISSLQS<br>EDFAVYYCQQYETFGQGTKVEIK
VRC18b V.Rev	VH reverted	QVQLVQSGAEVKKPGASVKVSCKASGYTFTGYYMHWVRQAPGQGLEWMGWINPNSGGTNYAQKFQGRVTMTRDTSISTAY<br>MELSRLRSDDTAVYYCARFAGYEWSFIWGQGTLVIVSS
	VL reverted	EIVLTQSPGTLSLSPGERATLSCRASQSVSSSYLAWYQQKPGQAPRLLIYGASSRATGIPDRFSGSGSGTDFTLTISRLE<br>PEDFAVYYCQILEFFGRGTRVEMN

Table S2. Neutralization breadth and potency (IC_{50}, μg/ml) of VRC01 lineage antibodies. Related to Figure 5.

		IC50 ug/ml															
**Clade**	**Ab ID**	**BJOX2000**	**CE1176**	**X1632**	**X2278**	**398F1**	**25710**	**CNE8**	**TRO11**	**246F3**	**CE0217**	**CH119**	**CNE55**	**MLV-SVA**	**Positives**	**% neut**	**GeoMean**
3+6	VRC03g	>50	>50	0.023	0.023	0.113	0.234	7.333	0.064	34.983	10.777	1.695	0.311	>50	10	83.3	0.51
3+6	VRC03	>50	>50	0.036	0.023	0.163	0.146	47.416	0.162	>50	>50	5.942	0.847	>50	8	66.7	0.41
3+6	VRC03b	>50	>50	0.168	0.023	0.783	1.071	3.167	2.699	28.487	>50	>50	3.316	>50	8	66.7	1.13
3+6	DH651.9	>50	>50	0.411	0.023	0.674	0.787	2.6	2.175	>50	>50	>50	10.797	>50	7	58.3	0.84
3+6	VRC03f	>50	>50	0.023	0.023	0.067	0.099	4.685	0.036	41.524	31.667	1.48	0.092	>50	10	83.3	0.40
3+6	VRC03e	n/a	n/a	n/a	n/a	n/a	n/a	n/a	n/a	n/a	n/a	n/a	n/a	n/a	n/a	n/a	n/a
3+6	VRC03i	>50	3.606	5.292	0.023	1.231	0.092	42.641	2.605	>50	>50	19.746	6.551	>50	9	75.0	2.08
3+6	VRC03h	>50	6.837	0.023	0.023	0.028	0.027	12.122	0.023	>50	7.799	1.22	0.105	>50	10	83.3	0.24
3+6	VRC03d	>50	>50	0.028	0.023	0.07	0.085	18.503	0.044	>50	25.903	1.824	1.297	>50	9	75.0	0.39
3+6	VRC06b	>50	49.799	1.531	0.023	45.013	0.087	>50	2.992	>50	>50	9.031	>50	>50	7	58.3	2.11
3+6	VRC06d	>50	>50	15.649	0.376	>50	21.1	43.15	8.436	>50	>50	>50	>50	>50	5	41.7	8.53
3+6	VRC06g	>50	>50	28.897	2.767	>50	30.834	26.925	20.072	>50	>50	>50	>50	>50	5	41.7	16.79
3+6	VRC06e	>50	>50	37.006	0.436	>50	17.704	34.013	6.907	>50	>50	>50	>50	>50	5	41.7	9.23
3+6	VRC06f	>50	>50	23.891	0.466	>50	15.085	25.7	11.089	47.14	>50	>50	>50	>50	6	50.0	11.45
3+6	VRC06	>50	>50	36.969	1.118	>50	11.193	>50	>50	>50	>50	>50	>50	>50	3	25.0	7.73
3+6	DH651.8	>50	>50	>50	44.239	>50	>50	>50	>50	>50	>50	>50	>50	>50	1	8.3	44.24
3+6	VRC06c	>50	>50	18.239	0.506	>50	12.371	26.828	8.851	>50	>50	>50	44.475	>50	6	50.0	10.32
8	VRC08e	31.58	1.773	>50	0.137	2.064	0.344	1.932	0.37	1.336	0.394	1.365	1.061	>50	11	91.7	1.10
8	VRC08c	>50	11.651	>50	0.275	18.772	1.312	0.819	1.041	2.704	5.74	>50	0.927	>50	9	75.0	2.15
8	VRC08d	40.754	0.835	>50	0.088	0.57	0.21	0.438	0.255	0.371	0.227	36.206	0.315	>50	11	91.7	0.74
8	VRC08	0.413	0.416	16.408	0.028	0.359	0.023	0.248	0.088	0.316	0.049	0.044	0.032	>50	12	100.0	0.16
1+7	DH651.3	>50	4.223	>50	0.512	1.185	2.514	7.767	1.261	1.193	0.636	3.681	1.197	>50	10	83.3	1.71
1+7	VRC02	>50	2.971	0.082	0.188	0.336	0.726	0.749	0.67	0.524	0.778	2.474	0.687	>50	11	91.7	0.60
1+7	DH651.4	>50	3.316	0.209	0.284	0.451	1.557	1	1.079	0.546	0.566	3.54	0.558	>50	11	91.7	0.80
1+7	VRC01c	>50	2.24	0.093	0.09	0.24	0.663	0.284	0.53	0.494	0.746	1.372	0.408	>50	11	91.7	0.43
1+7	DH651.2	>50	7.528	0.2	0.397	0.558	1.525	1.314	1.35	0.997	0.851	4.168	0.941	>50	11	91.7	1.11
1+7	VRC01g	>50	2.795	0.137	0.211	0.218	0.754	1.561	0.684	0.361	0.29	0.754	0.545	>50	11	91.7	0.51
1+7	VRC01b	>50	10.813	0.886	0.528	1.254	2.617	2.254	2.305	1.309	2.227	12.337	2.879	>50	11	91.7	2.29
1+7	VRC01	>50	1.261	0.035	0.069	0.121	0.326	0.144	0.287	0.205	0.288	0.562	0.359	>50	11	91.7	0.22
1+7	DH651.1	>50	6.771	0.279	0.66	0.547	2.295	2.997	2.071	1.014	0.83	3.754	0.831	>50	11	91.7	1.34
1+7	VRC01i	>50	>50	>50	0.842	0.561	>50	45.556	>50	3.823	4.352	>50	1.361	>50	6	50.0	2.81
1+7	VRC01h	>50	1.709	0.133	0.164	0.2	0.585	0.238	0.386	0.391	0.409	1.482	0.382	>50	11	91.7	0.39
1+7	VRC01f	>50	2.396	0.104	0.119	0.236	0.515	0.341	0.408	0.373	0.289	1.331	0.484	>50	11	91.7	0.39
1+7	VRC01e	19.175	2.365	0.079	0.126	0.118	0.54	0.744	0.445	0.261	0.209	0.468	0.251	>50	12	100.0	0.45
1+7	VRC01j	n/a	n/a	n/a	n/a	n/a	n/a	n/a	n/a	n/a	n/a	n/a	n/a	n/a	n/a	n/a	n/a
1+7	VRC01d	40.241	2.325	0.157	0.138	0.161	0.541	0.772	0.535	0.302	0.22	0.478	0.365	>50	12	100.0	0.55
1+7	VRC07e	>50	2.921	0.156	0.099	0.225	0.681	0.554	5.526	1.576	0.204	0.996	0.373	>50	11	91.7	0.58
1+7	VRC07f	>50	9.689	0.634	0.09	0.402	1.835	1.402	>50	2.145	0.25	1.131	0.634	>50	10	83.3	0.86
1+7	VRC07d	>50	1.801	0.142	0.051	0.189	0.507	0.487	1.998	0.581	0.097	0.177	0.149	>50	11	91.7	0.30
1+7	NIH45-46	>50	2.687	0.086	0.06	0.337	0.554	0.33	9.089	0.761	0.135	0.206	0.139	>50	11	91.7	0.38
1+7	DH651.7	>50	>50	>50	0.201	2.34	>50	>50	>50	36.307	25.933	>50	34.339	>50	5	41.7	6.86
1+7	DH651.5	>50	11.672	2.521	0.243	0.521	1.101	1.592	6.515	0.806	0.336	3.101	1.009	>50	11	91.7	1.39
1+7	DH651.6	>50	35.161	1.027	0.262	0.309	2.688	3.805	>50	4.583	0.733	5.672	1.838	>50	10	83.3	2.00
1+7	VRC07c	>50	0.945	0.023	0.088	0.222	0.351	0.275	0.51	0.469	0.32	0.399	0.178	>50	11	91.7	0.25
1+7	VRC07b	>50	1.276	0.023	0.063	0.259	0.376	0.243	0.574	0.428	0.398	0.442	0.223	>50	11	91.7	0.26
neg ctrl	Ab82	>50	>50	>50	>50	>50	>50	>50	>50	>50	>50	>50	>50	>50	0	0.0	n/a
post ctrl	CH01+CH31	8.303	0.191	0.058	0.062	0.122	0.375	0.498	0.061	0.145	0.058	1.152	0.062	>50	12	100.0	0.20

Table S3. Neutralization breadth and potency (IC_{80}, μg/ml) of VRC01 lineage antibodies. Related to Figure 5.

		IC80 ug/ml															
Clade	Ab ID	BJOX2000	CE1176	X1632	X2278	398F1	25710	CNE8	TRO11	246F3	CE0217	CH119	CNE55	MLV-SVA	Positives	% neut	GeoMean
3+6	VRC03g	>50	>50	0.111	0.023	0.505	0.711	25.185	0.338	>50	>50	10.55	1.597	>50	8	66.7	0.78
3+6	VRC03	>50	>50	0.164	0.023	0.61	0.562	>50	0.653	>50	>50	>50	3.114	>50	6	50.0	0.37
3+6	VRC03b	>50	>50	1.467	0.075	3.282	7.338	20.092	19.492	>50	>50	>50	20.876	>50	7	58.3	4.16
3+6	DH651.9	>50	>50	2.77	0.061	2.406	3.176	8.376	11.439	>50	>50	>50	48.574	>50	7	58.3	3.47
3+6	VRC03f	>50	>50	0.04	0.023	0.301	0.401	20.35	0.149	>50	>50	7.991	0.44	>50	8	66.7	0.43
3+6	VRC03e	n/a	n/a	n/a	n/a	n/a	n/a	n/a	n/a	n/a	n/a	n/a	n/a	n/a	n/a	n/a	n/a
3+6	VRC03i	>50	14.432	>50	0.076	7.9	0.436	>50	11.269	>50	>50	>50	42.864	>50	6	50.0	3.50
3+6	VRC03h	>50	24.802	0.083	0.023	0.133	0.129	44.493	0.08	>50	>50	26.99	0.291	>50	9	75.0	0.66
3+6	VRC03d	>50	>50	0.222	0.023	0.635	0.308	>50	0.181	>50	>50	>50	8.891	>50	6	50.0	0.34
3+6	VRC06b	>50	>50	>50	0.029	>50	0.345	>50	15.846	>50	>50	>50	>50	>50	3	25.0	0.54
3+6	VRC06d	>50	>50	>50	1.989	>50	>50	>50	49.759	>50	>50	>50	>50	>50	2	16.7	9.95
3+6	VRC06g	>50	>50	>50	10.896	>50	>50	>50	>50	>50	>50	>50	>50	>50	1	8.3	10.90
3+6	VRC06e	>50	>50	>50	1.511	>50	>50	>50	31.173	>50	>50	>50	>50	>50	2	16.7	6.86
3+6	VRC06f	>50	>50	>50	2.598	>50	>50	>50	47.161	>50	>50	>50	>50	>50	2	16.7	11.07
3+6	VRC06	>50	>50	>50	3.648	>50	45.597	>50	>50	>50	>50	>50	>50	>50	2	16.7	12.90
3+6	DH651.8	>50	>50	>50	>50	>50	>50	>50	>50	>50	>50	>50	>50	>50	0	0.0	n/a
3+6	VRC06c	>50	>50	>50	2.056	>50	47.322	>50	33.803	>50	>50	>50	>50	>50	3	25.0	14.87
8	VRC08e	>50	6.805	>50	0.376	10.916	1.024	6.434	1.801	4.843	1.631	7.469	3.875	>50	10	83.3	3.08
8	VRC08c	>50	>50	>50	0.693	>50	6.51	3.59	2.816	14.352	>50	>50	3.353	>50	6	50.0	3.60
8	VRC08d	>50	3.75	>50	0.273	2.254	0.804	1.596	0.85	1.145	0.798	>50	1.012	>50	9	75.0	1.10
8	VRC08	1.049	1.156	>50	0.096	1.685	0.207	0.581	0.256	0.902	0.201	0.182	0.274	>50	11	91.7	0.41
1+7	DH651.3	>50	>50	>50	1.672	4.264	9.26	43.453	4.234	3.26	2.686	10.942	2.647	>50	9	75.0	5.26
1+7	VRC02	>50	15.474	0.64	0.658	1.109	2.568	2.106	1.969	1.511	2.707	9.287	2.032	>50	11	91.7	2.20
1+7	DH651.4	>50	18.768	3.156	0.956	1.441	6.861	3.829	3.014	1.615	1.874	10.139	1.522	>50	11	91.7	3.15
1+7	VRC01c	>50	8.63	1.286	0.358	0.785	2.242	1.147	1.507	1.318	1.731	3.811	1.205	>50	11	91.7	1.55
1+7	DH651.2	>50	23.543	6.633	1.192	1.853	5.132	4.428	3.774	2.331	2.867	15.198	2.671	>50	11	91.7	4.24
1+7	VRC01g	>50	7.576	0.814	0.487	0.771	2.142	3.868	1.513	0.977	0.9	2.229	1.287	>50	11	91.7	1.48
1+7	VRC01b	>50	>50	26.282	1.933	4.466	9.419	9.171	6.368	3.594	7.456	36.239	7.342	>50	10	83.3	7.85
1+7	VRC01	>50	3.879	0.246	0.211	0.46	1.155	0.55	0.918	0.62	0.825	1.602	0.995	>50	11	91.7	0.75
1+7	DH651.1	>50	21.744	2.94	1.465	1.616	6.133	9.539	5.275	2.779	2.425	13.632	2.395	>50	11	91.7	4.34
1+7	VRC01i	>50	>50	>50	2.808	2.266	>50	>50	>50	1.385	12.322	>50	4.967	>50	5	41.7	3.52
1+7	VRC01h	>50	8.512	1.502	0.413	0.744	2.096	1.449	1.402	1.297	0.991	4.259	1.179	>50	11	91.7	1.53
1+7	VRC01f	>50	8.889	1.16	0.437	0.809	1.817	1.731	1.488	1.017	1.307	3.831	1.129	>50	11	91.7	1.52
1+7	VRC01e	>50	6.56	0.62	0.32	0.346	1.624	2.091	1.23	0.633	0.61	1.215	0.744	>50	11	91.7	0.96
1+7	VRC01j	n/a	n/a	n/a	n/a	n/a	n/a	n/a	n/a	n/a	n/a	n/a	n/a	n/a	n/a	n/a	n/a
1+7	VRC01d	>50	6.549	1.746	0.407	0.658	2.053	2.214	1.224	0.897	0.632	1.517	0.89	>50	11	91.7	1.26
1+7	VRC07e	>50	18.219	28.882	0.343	1.128	3.272	1.947	>50	5.99	1.184	3.037	1.076	>50	10	83.3	2.80
1+7	VRC07f	>50	>50	>50	0.247	1.218	5.74	10.47	>50	6.805	1.256	3.27	2.349	>50	8	66.7	2.42
1+7	VRC07d	>50	4.614	15.461	0.138	0.598	1.538	1.288	7.608	2.01	0.386	0.442	0.496	>50	11	91.7	1.28
1+7	NIH45-46	>50	6.846	31.99	0.212	0.914	2.65	1.571	>50	1.902	0.46	0.535	0.346	>50	10	83.3	1.40
1+7	DH651.7	>50	11.315	>50	0.956	13.828	>50	>50	>50	>50	>50	>50	>50	>50	3	25.0	5.31
1+7	DH651.5	>50	>50	>50	0.833	2.031	3.752	6.059	39.12	2.314	2.015	8.357	2.995	>50	9	75.0	3.83
1+7	DH651.6	>50	>50	>50	0.691	1.444	11.09	37.341	>50	19.969	2.423	17.832	6.56	>50	8	66.7	6.25
1+7	VRC07c	>50	3.804	0.14	0.262	0.964	1.331	0.795	1.445	1.371	0.62	1.175	0.652	>50	11	91.7	0.83
1+7	VRC07b	>50	4.815	0.131	0.215	0.873	1.12	0.978	2.066	1.252	1.458	1.319	0.801	>50	11	91.7	0.94
neg ctrl	Ab82	>50	>50	>50	>50	>50	>50	>50	>50	>50	>50	>50	>50	>50	0	0.0	n/a
post ctrl	CH01+CH31	>50	1.01	0.202	0.32	0.345	1.274	1.633	0.69	0.403	0.226	4.137	0.38	>50	11	91.7	0.61

a

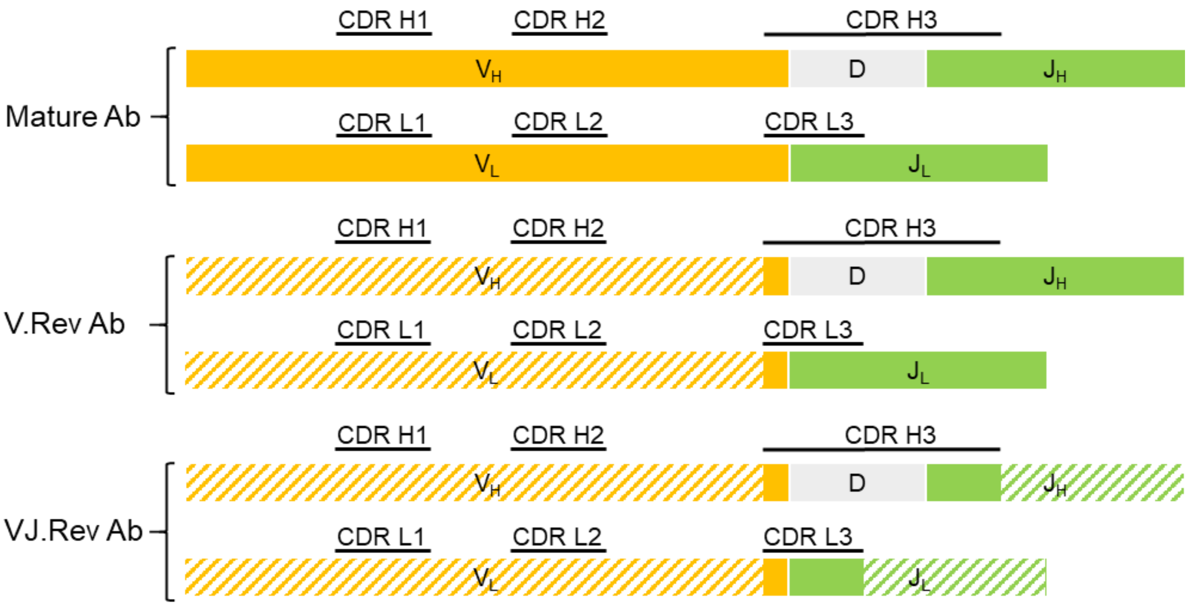

b

```
VRC01 bnAb VHDJH rearrangement
--------------FR1-------------_CDR1_------FR2-----______CDR2______-----------FR3------
QVQLVQSGGQMKKPGESMRISCRASGYEFIDCTLNWIRLAPGKRPEWMGWLKPRGGAVNYARPLQGRVTMTRDVYSDTAFLELRSL
                             •  •             •  • • ••••••••••• •   • • ••

------------____CDR3____----FR4----
TVDDTAVYFCTRGKNCDYNWDFEHWGRGTPVIVSS
               ••••


VRC01 bnAb VLJL rearrangement
----------FR1----------___CDR1__------FR2------__CDR2_--------------FR3---------------
EIVLTQSPGTLSLSPGETAIISCRTSQYGSLAWYQQRPGQAPRLVIYSGSTRAAGIPDRFSGSRWGPDYNLTISNLESGDFGVYYC
  •                       ••••

CDR3_-----FR4-----
QQYEFFGQGTKVQVDIKR
  •••
```

Figure S1. Schematic representation of the reversions introduced in "germline-reverted" antibodies related to the nomenclature used in this paper. Related to Figure 1. (a) The scheme shows the V(D)J rearrangements on the mature antibodies in solid color. Previously published "germline-reverted" antibodies comprise different designs in which parts of the V(D)J sequences were reverted to the unmutated sequence of the gene segment of reference (called V.Rev and VJ.Rev in this paper). The reverted parts are shown with patterned lines. (b) The V(D)J rearrangement sequences of the mature VRC01 bnAb heavy (top) and light (bottom) chains is color coded using the same scheme of panel a. Amino acids shown in black are encoded by n-nucleotide insertions. Contact sites with gp120 as described in Wu et al. 2010 are shown with solid circles as in Figure 1.

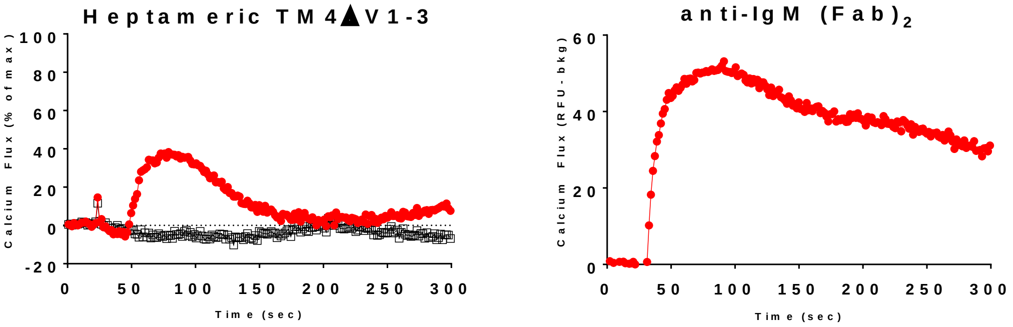

Figure S2. Effect of multimerization on TM4ΔV1-3 binding to VRC01 UCA. Related to Figure 2. Activation of Ramos B cell lines expressing the VRC01 UCA IgM BCR on cell surface was determined by measuring calcium flux upon binding. TM4ΔV1-3 core was heptamerized (left). Calcium flux is reported as % of maximum flux induced by anti-IgM $(Fab)_2$ (right). All data are representative of duplicate experiments.

a

mAb ID	CDR H3 Amino Acid Sequence	Length	Identity
VRC01 UCA	CAR--------GGYCSGGSCY-NWD---FQHW	16	
VRC01c-HuGL1	CAR---------IY-SGYDL---WW---FDPW	12	31%
VRC01c-HuGL2	CAK-----------ISG-----SYS---FDYW	9	19%
VRC01c-HuGL3	CAR----------------MY-NWNDVWFDPW	11	21%
VRC01c-HuGL4	CAR----ASRLGGY--------------FQHW	10	30%
VRC01c-HuGL5	CAK-------------------HHIRGWFDPW	8	5%
VRC01c-HuGL6	CAR--------VDY---GDYYGSWY---FDLW	14	29%
VRC01c-HuGL7	CAR-------SDGYNLG------WY---FDLW	12	29%
VRC01c-HuGL8	CAL-------SPYYDS--SGY-------FD-W	11	29%
VRC01c-HuGL9	CAR-----------DS------NWW---FDPW	8	25%
VRC01c-HuGL10	CAR--------RQYCSGGSCL--YL---FDPW	15	50%
VRC01c-HuGL11	CAS--------KVAAAGTLAK---DA--FDIW	15	18%
VRC01c-HuGL12	CAR-----------AAIAAAY------FRDPW	11	6%
VRC01c-HuGL13	CAR--------DKAVAGT----N-----FDYW	11	19%
VRC01c-HuGL14	CARRGLLGRGYSGYDRMGYYY-YYG---MDVW	24	17%
VRC01c-HuGL15	CAR--------PTEYS--SSW-YW----FDPW	13	25%
VRC01c-HuGL16	CARDHQ-----GH--S--SSW-SKR---FDYW	15	21%
VRC01c-HuGL17	CAR---------VIRS--SSS--WR---YDYW	12	19%
VRC01c-HuGL18	CAR--------VRYGSWTGYY-------FDYW	13	25%
VRC01c-HuGL19	CAR--------VPYDFWSGYY-VLS--HFDYW	17	18%
VRC01c-HuGL20	CAR--------LVGATGTS---------EDYW	11	13%
VRC01c-HuGL21	CAR--------EGRGYSTGAY-------FDYW	13	19%
VRC01c-HuGL22	CAR--------PPGPAVAGRY-NWW---FDPW	16	25%
VRC01c-HuGL23	CAR--------------GSRA-TWI---QLHW	10	25%
VRC01c-HuGL24	CAR--------VGEQLVL----N-DA--FDIW	13	19%
VRC01c-HuGL25	CAR--------DLTEVTT----------PPPW	10	0%

b

mAb ID	CDR H3 Amino Acid Sequence	Length	Identity
VRC01 UCA	CAR--GGYCSGGSCYNWDFQHW	16	
1539-B10_H	CAR--RVY------GNYDWAYW	10	19%
1539-B9_H	CAR--RRY-----NYDWEFVYW	11	25%
1540-E9_H	CAR--K--------TTMVFDYW	8	7%
1536-hvk-E	CAR--PGYGN----YGWYFDVW	12	31%
1539-A1_H	CAA--AYYNYDAESFDWYFDVW	16	19%
1539-B3_H	CAI---YYVYG----DWYFDVW	11	19%
1539-B5_H	CAV---YYGYG----DWYFDVW	11	25%
1539-C8_H	CAR-RGDYDEG----DWYFHVW	13	29%
1539-E6_H	CAR--HIFDS-----HWYFDVW	11	19%
1539-G2_H	CAR--P---STYDDYDWYFDVW	13	25%
1539-G5_H	CAK--D--MSG---TDWHLDVW	11	19%
1539-G6_H	CAR--PSYDYDH---DWYFDVW	13	19%
1538-1_H	CAR--YDNDVDG----WYFDVW	12	19%
1538-4_H	CAR--YDHDGPG----WYFDVW	12	19%
1538-12_H	CARYRNSYDDDG----WKFDIW	14	22%
1538-17_H	CAR--YDNDDDG----WSFDVW	12	19%
1538-19_H	CAR--YDNDGDG----WSFDVW	12	19%
1538-20_H	CAR--YDNDADG----WFFDVW	12	19%
1538-86_H	CAR--YDNDADG----WYFDVW	12	19%
1538-26_H	CAR--YNNEEDG----WYFDVW	12	19%
1538-65_H	CAR--YDSDGEG----WYFDVW	12	19%
1538-76_H	CAR--YDNDGEG----WYFDVW	12	19%
1538-67_H	CAR--VDYDYDGRDY-YAMDYW	15	19%
1538-69_H	CAR--DR--TGN---DWNFDVW	11	19%
1538-79_H	CAR--DR--TGN---DWYFDVW	11	19%
1538-91_H	CARELGLA--------W-FAYW	9	17%
1538-93_H	CAR--D---SSGSA--W-FAYW	10	31%

Figure S3. CDR H3 aa alignments of VRC01-class putative precursors isolated using immunogens optimized for binding to GL VRC01-class bnAbs. Related to Figure 2. CDR H3 sequences from (a) human naïve B cell-derived antibodies elicited by eOD-GT8 (Jardine et al., 2016a) or (b) induced by a step-wise immunization in V_H1-2*02/precursor VRC01 IgL knock-in mice (Tian et al., 2016) were independently aligned to VRC01 UCA using EMBOSS Water and the final alignment was manually assembled. The CAR motif preceding CDR H3 and the constant tryptophan at the end of CDR H3 are shown in gray. CDR H3 lengths are reported in aa their identity to VRC01 UCA was calculated on pairwise alignments.

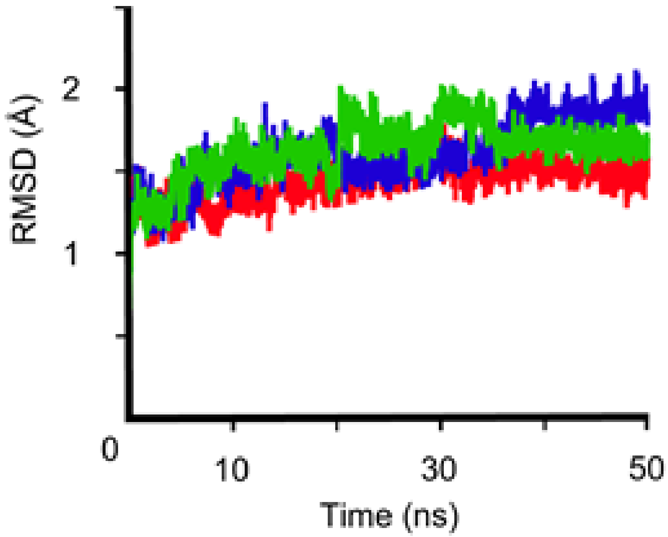

Figure S4. RMSD plot for each VRC08 CDR H3 loop during the molecular dynamics simulation. Related to Figure 6. Protein backbone RMSD of each JR-FL bound VRC08 Fab CDR H3 loop (each depicted individually in red, green, or blue) relative to the initial loop configuration during the 50 ns molecular dynamics simulation.

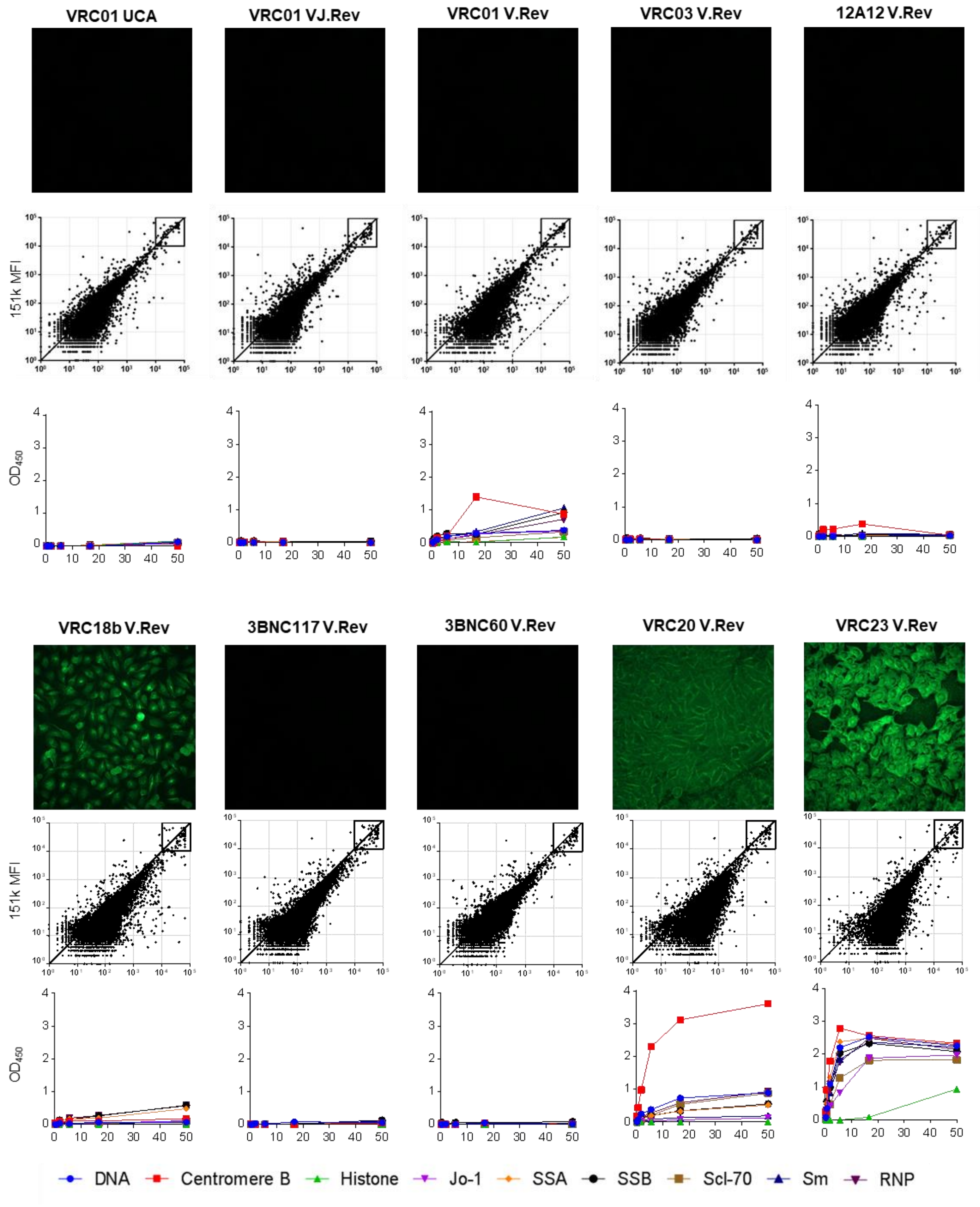

Figure S5. Auto- and polyreactivity of VRC01 UCA and VRC01-class germline-reverted antibodies. Related to Figure 7. Top: HEp-2 cell IFA staining. Middle: measurement of polyreactivity against 9,400 human antigens using the ProtoArray 5 microchip: VRC01 UCA and VRC01-class reverted mAb binding (x-axis) were compared to non-polyreactive control mAb 151k (y-axis). Significant polyreactivity is defined as 1 log more avid binding than the 151k mAb to more than 90% of the test proteins (Liu et al., 2015) and it is visualized by displacement towards the x-axis. Bottom: VRC01 UCA and VRC01-class reverted mAb binding to an ANA panel comprising 9 antigens, measured in ELISA. Results are representative of duplicate experiments and are shown background subtracted. Positivity is defined by two consecutive dilutions with $OD_{450}>0.2$.

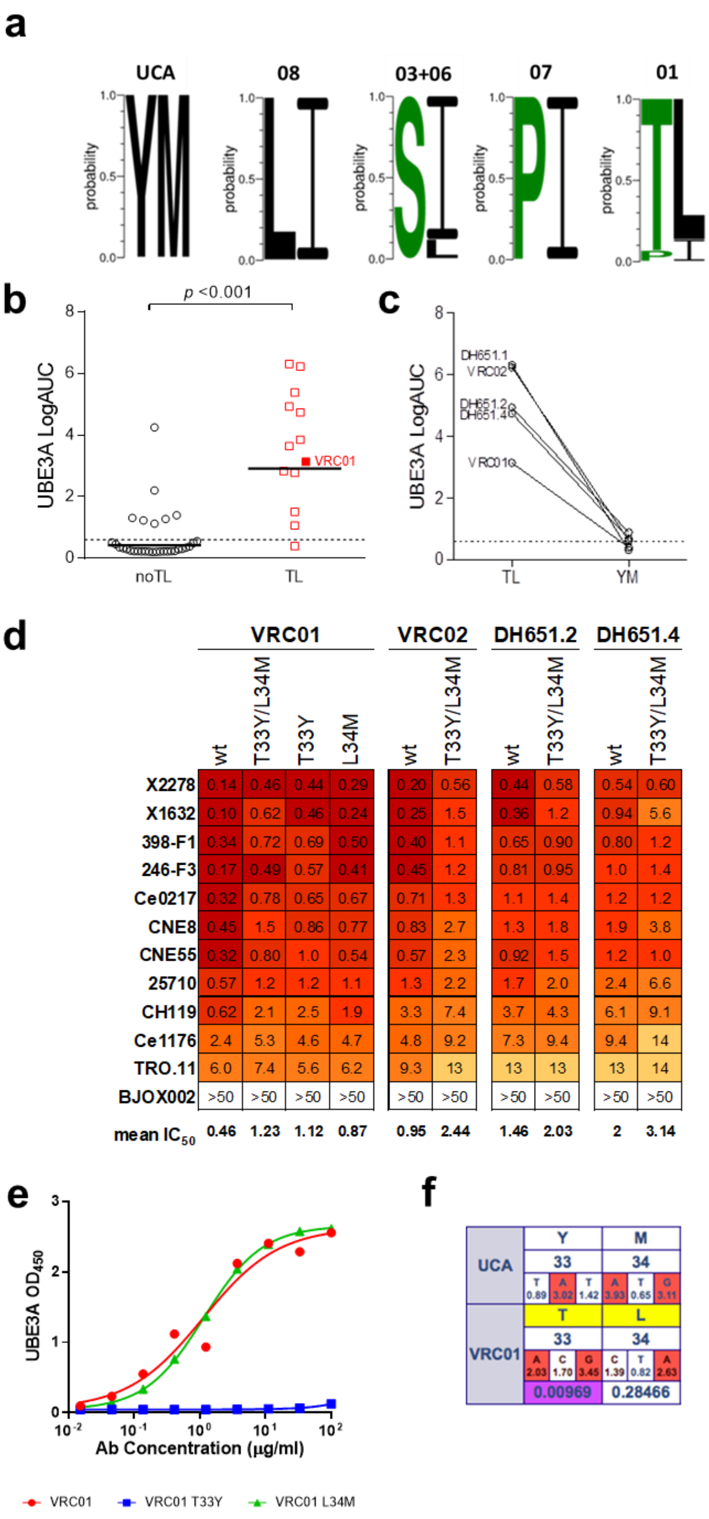

Figure S6. Reversion mutation T33Y in CDR H1 abrogates VRC01 UBE3A reactivity. Related to Figure 7. (a) Logo plot showing the frequency of mutations at positions 34 and 35 in the CDR H1 of mAbs in each clade of the VRC01 lineage. Subclades 01 and 07 are shown separately as they display distinct profiles. (b) UBE3A binding expressed as LogAUC (y-axis) of the observed VRC01 lineage mAbs is significantly associated with presence of the ^{33}TL^{34} motif. Mabs with TL mutations (n=13) are shown in clear red squares and the VRC01 bnAb is shown in solid red. Mabs without the TL mutation (n=31) are shown in clear black circles. Lines at geometric mean. Significance was evaluated with the Mann-Whitney U-test at the alpha 0.05 level. (c) Double reversion of TL to germline YM abrogates UBE3A binding of subclade 01 bnAbs VRC01, VRC02, DH651.1, DH651.2 and DH651.4. Dotted line at limit of detection. (d) Heat map analysis of neutralization of VRC01, VRC02, DH651.2 and DH651.4 bnAbs and respective single and/or double mutants against the 12-virus global panel. Neutralization potency IC_{50} is expressed in µg/ml and coloring ranges from white (>50µg/ml) to dark red (<0.023µg/ml). (e) Binding to UBE3A measured in ELISA by VRC01 bnAb (red) and single mutants VRC01 T33Y (blue) and VRC01 L34M (green). (f) Probability of the TL mutation in VRC01 bnAb CDR H1: ARMADiLLO output for VRC01 bnAb amino acids T33 and L34. Rows 1-3: VRC01 UCA aa, position and codon mutability score; Rows 4-6: VRC01 bnAb aa, position and codon mutability score; Row 7: probability of aa mutation. Colors: Blue: cold spot; Red: hot spot; Yellow: aa change; Magenta: improbable mutation (Wiehe et al., 2018).
